# Evolutionary Dynamics of Chromatin Structure and Duplicate Gene Expression in Diploid and Allopolyploid Cotton

**DOI:** 10.1093/molbev/msae095

**Published:** 2024-05-17

**Authors:** Guanjing Hu, Corrinne E Grover, Daniel L Vera, Pei-Yau Lung, Senthil B Girimurugan, Emma R Miller, Justin L Conover, Shujun Ou, Xianpeng Xiong, De Zhu, Dongming Li, Joseph P Gallagher, Joshua A Udall, Xin Sui, Jinfeng Zhang, Hank W Bass, Jonathan F Wendel

**Affiliations:** State Key Laboratory of Cotton Bio-breeding and Integrated, Chinese Academy of Agricultural Sciences, Institute of Cotton Research, Anyang 455000, China; Shenzhen Branch, Guangdong Laboratory of Lingnan Modern Agriculture, Key Laboratory of Synthetic Biology, Ministry of Agriculture and Rural Affairs, Chinese Academy of Agricultural Sciences, Agricultural Genomics Institute at Shenzhen, Shenzhen 518120, China; Department of Ecology, Evolution and Organismal Biology, Iowa State University, Ames, IA 50011, USA; Department of Biological Science, Florida State University, Tallahassee, FL 32306, USA; Department of Statistics, Florida State University, Tallahassee, FL 32306, USA; Department of Mathematics, Florida Gulf Coast University, Fort Myers, FL 33965, USA; Department of Ecology, Evolution and Organismal Biology, Iowa State University, Ames, IA 50011, USA; Department of Ecology, Evolution and Organismal Biology, Iowa State University, Ames, IA 50011, USA; Department of Ecology and Evolutionary Biology, University of Arizona, Tucson, AZ 85721, USA; Department of Molecular and Cellular Biology, University of Arizona, Tucson, AZ 85721, USA; Department of Molecular Genetics, Ohio State University, Columbus, OH 43210, USA; Shenzhen Branch, Guangdong Laboratory of Lingnan Modern Agriculture, Key Laboratory of Synthetic Biology, Ministry of Agriculture and Rural Affairs, Chinese Academy of Agricultural Sciences, Agricultural Genomics Institute at Shenzhen, Shenzhen 518120, China; Shenzhen Branch, Guangdong Laboratory of Lingnan Modern Agriculture, Key Laboratory of Synthetic Biology, Ministry of Agriculture and Rural Affairs, Chinese Academy of Agricultural Sciences, Agricultural Genomics Institute at Shenzhen, Shenzhen 518120, China; Shenzhen Branch, Guangdong Laboratory of Lingnan Modern Agriculture, Key Laboratory of Synthetic Biology, Ministry of Agriculture and Rural Affairs, Chinese Academy of Agricultural Sciences, Agricultural Genomics Institute at Shenzhen, Shenzhen 518120, China; Zhengzhou Research Base, State Key Laboratory of Cotton Biology, School of Agricultural Sciences, Zhengzhou University, Zhengzhou 450000, China; Forage Seed and Cereal Research Unit, USDA/Agricultural Research Service, Corvallis, OR 97331, USA; Crop Germplasm Research Unit, USDA/Agricultural Research Service, College Station, TX 77845, USA; Department of Statistics, Florida State University, Tallahassee, FL 32306, USA; Department of Statistics, Florida State University, Tallahassee, FL 32306, USA; Department of Biological Science, Florida State University, Tallahassee, FL 32306, USA; Department of Ecology, Evolution and Organismal Biology, Iowa State University, Ames, IA 50011, USA

**Keywords:** allopolyploidy, chromatin accessibility, nucleosome organization, cotton, genome dominance, homoeolog expression bias

## Abstract

Polyploidy is a prominent mechanism of plant speciation and adaptation, yet the mechanistic understandings of duplicated gene regulation remain elusive. Chromatin structure dynamics are suggested to govern gene regulatory control. Here, we characterized genome-wide nucleosome organization and chromatin accessibility in allotetraploid cotton, *Gossypium hirsutum* (AADD, 2*n* = 4*X* = 52), relative to its two diploid parents (AA or DD genome) and their synthetic diploid hybrid (AD), using DNS-seq. The larger A-genome exhibited wider average nucleosome spacing in diploids, and this intergenomic difference diminished in the allopolyploid but not hybrid. Allopolyploidization also exhibited increased accessibility at promoters genome-wide and synchronized *cis*-regulatory motifs between subgenomes. A prominent *cis*-acting control was inferred for chromatin dynamics and demonstrated by transposable element removal from promoters. Linking accessibility to gene expression patterns, we found distinct regulatory effects for hybridization and later allopolyploid stages, including nuanced establishment of homoeolog expression bias and expression level dominance. Histone gene expression and nucleosome organization are coordinated through chromatin accessibility. Our study demonstrates the capability to track high-resolution chromatin structure dynamics and reveals their role in the evolution of *cis*-regulatory landscapes and duplicate gene expression in polyploids, illuminating regulatory ties to subgenomic asymmetry and dominance.

## Introduction

Polyploidy is a widespread biological phenomenon in eukaryotes and is important in all levels of biological organization (Fox et al. 2020). Being exceptionally prevalent in ferns and flowering plants (Jiao et al. 2011; Ruprecht et al. 2017; Initiative & One Thousand Plant Transcriptomes Initiative 2019), whole-genome duplications resulting from polyploidy have significant implications for plant physiology, ecology, and evolution (Stebbins 1940; Levin 1983; Ramsey and Schemske 2002; Leitch and Leitch 2008; Wendel 2015; Soltis and Soltis 2016; Van de Peer et al. 2017, 2021; Wendel et al. 2018; Heslop-Harrison et al. 2022). Polyploidy may be associated with expanded ecological ranges (Arrigo et al. 2016; Coughlan et al. 2017; Baniaga et al. 2020; Wang et al. 2021; Parshuram et al. 2022; Zhao et al. 2022; Elliott et al. 2023; Mata et al. 2023), enhanced tolerance to biotic and abiotic stresses (reviewed in Van de Peer et al. 2021), physiological changes (Mishra 1997; Sugiyama 2005; Otto 2007; Knight and Beaulieu 2008; Coate et al. 2012; Orr-Weaver 2015), and altered biosynthetic pathways (Combes et al. 2022). These changes may confer economically or ecologically important traits (Heslop-Harrison et al. 2022). Unsurprisingly, numerous vital crop species are relatively young polyploids (Olsen and Wendel 2013; Renny-Byfield and Wendel 2014; Zhang et al. 2019; Heslop-Harrison et al. 2022).

Increases in whole-genome content resulting from polyploidy are often associated with changes in nucleotypic characters, such as cell size, nuclear volume, and cell cycle duration (Wendel et al. 2018; Doyle and Coate 2019). These genomic changes may also alter epigenetic dynamics, gene expression, the proteome, and molecular networks. One extensively demonstrated effect is the profound rewiring of transcriptomes in response to genomic merger and doubling during allopolyploidization (Grover et al. 2012; Hu and Wendel 2019; Visger et al. 2019; Shan et al. 2020; Giraud et al. 2021). This genome-wide rewiring encompasses a diversity of phenomena, including unequal expression of homoeologs at the genic level (referred to as “homoeolog expression bias”) (Flagel et al. 2008; Grover et al. 2012) or the genomic level (genome dominance) (Schnable et al. 2011), inconsistency in homoeolog biases across tissues or conditions (expression subfunctionalization and neofunctionalization) (Adams et al. 2003) even at the single-cell level (Zhang et al. 2023), apparent *trans*-control of duplicate expression (expression level dominance) (Rapp et al. 2009; Grover et al. 2012; Yoo and Wendel 2014; Yoo et al. 2014), and altered coexpression gene networks (Gallagher et al. 2016; Hu et al. 2016). While these studies shed light on the evolutionary dynamics of polyploid transcriptomes, the mechanistic underpinnings of these phenomena remain elusive, limiting our understanding of duplicate gene expression evolution, and hence the origin of evolutionary innovation accompanying polyploidy.

The study of chromatin structure has emerged as a field that may bridge the gap between genome evolution and transcriptome evolution, providing insights into the dynamics of gene expression regulation. The chromatin structure landscape reflects multiple and complex regulatory layers that fine-tune gene expression (Talbert et al. 2019; Ahmad et al. 2022). Nucleosomes, the fundamental structural units of chromatin, consist of 147 bases of DNA wrapped around a core histone octamer (Luger et al. 1997). Facilitating the compaction of genomic DNA into chromatin, nucleosomes play a crucial role in controlling DNA accessibility for processes such as gene transcription, DNA replication, repair, and recombination (Kornberg 1974; Andrews and Luger 2011). During transcriptional activation, nucleosomes can be moved to expose or conceal *cis*-regulatory DNA sites, or transiently destabilized (referred to as “fragile” nucleosomes) at promoter regions (Zlatanova et al. 2008; Mieczkowski et al. 2016; Klemm et al. 2019). Thus, nucleosomes act as regulators of chromatin accessibility, which inherently manifests the myriad epigenetic modifications of histones and DNA that collectively control gene expression (Schmitz et al. 2011; Jordan and Schmitz 2016; Kawakatsu et al. 2016; Niederhuth et al. 2016; Hofmeister et al. 2017; Jackson 2017; Song et al. 2017; Springer and Schmitz 2017; Giles and Taberlay 2019; Klein and Hainer 2020). Understanding the factors that determine nucleosome properties and their impact on chromatin accessibility and gene activity is a central biological challenge.

Over the past decade, high-throughput techniques have been employed in plants to map nucleosome occupancy and chromatin accessibility at a genome-wide scale (Tsompana and Buck 2014; Liu et al. 2015; Zhang et al. 2015; Voong et al. 2017; Zhang and Jiang 2018; Baldi et al. 2020; Galli et al. 2020; Jordan et al. 2020; Zhao et al. 2020; Barbier et al. 2021). These methods, including micrococcal nuclease sequencing (MNase-seq), DNase I hypersensitive site sequencing (DNase-seq), and assay for transposase accessible chromatin sequencing (ATAC-seq), are based on the physical accessibility of chromatin to nucleases. The nuclease cleavage patterns are used to distinguish accessible DNA regions from nucleosome-protected or transcription factor (TF)-protected regions through fragmentation, tagmentation, or elimination. Since the 1970s, DNase I hypersensitive sites (DHSs) have been considered a hallmark of active regulatory regions in eukaryotic genomes (Weintraub and Groudine 1976; Wu et al. 1979a, 1979b). High-throughput DHS mapping has provided genome-wide insight into *cis*-regulatory DNA elements (CREs) and TF binding sites (TFBSs) in various plant species (Zhang et al. 2012; Jiang 2015; Sullivan et al. 2015; Qiu et al. 2016; Zhao et al. 2018; Han et al. 2020, 2022). ATAC-seq, a more efficient alternative to DNase-seq, enables fast and low-input profiling of chromatin accessibility (Lu et al. 2017), even at the single-cell level (Dorrity et al. 2021). These techniques, along with their variants, have provided insights into *cis*-regulatory landscapes and gene regulatory networks in plant species (Lu et al. 2019; Ricci et al. 2019; Reynoso et al. 2022).

MNase-seq, on the other hand, is historically used for profiling nucleosome occupancy and has been demonstrated in plants such as *Arabidopsis* (Chodavarapu et al. 2010; Li et al. 2014; Liu et al. 2015) and rice (Wu et al. 2014; Zhang et al. 2015). Recent applications of this technique utilize two micrococcal nuclease (MNase) digest conditions, light and heavy, which provide both nucleosome positioning data and chromatin accessibility/sensitivity profiling (Vera et al. 2014; Rodgers-Melnick et al. 2016). That is, differential nuclease sensitivity (DNS) profiling of nucleosome occupancy leads to identifying various levels of chromatin accessibility; this approach was first established in maize based on DNA microarray (Vera et al. 2014), and next employed high-throughput sequencing for genome-wide profiling (Rodgers-Melnick et al. 2016). Like DHS identified by DNase-seq and ATAC-seq, the MNase sensitive footprints (MSFs) from differential sensitivity MNase-seq (DNS-seq) are enriched at the 5′ and 3′ boundaries of genes, and are positively associated with gene expression levels, DNA hypomethylation, conserved noncoding sequences, and known TFBSs. In maize, MNase hypersensitive regions account for <1% of the genome, but are linked to genotypic variants that explain ∼40% of variation in phenotypic traits, on a par with coding regions (∼48%) (Rodgers-Melnick et al. 2016). Additionally, MNase-profiled *cis*-regulatory landscapes have been linked to tissue-specific transcription and environmental responses, highlighting their roles in shaping phenotypic variation (Pass et al. 2017; Parvathaneni et al. 2020). A related assay based on small DNA fragments from light MNase digestion, MOA-seq, was recently developed to map small particles that delineate likely TF occupancies at *cis*-regulatory elements within accessible chromatin regions (Savadel et al. 2021; Liang et al. 2022). Overall, the properties of MNase as a probe for chromatin structure have proven highly informative for characterizing chromatin landscapes, nucleosome positioning, nucleosome stability, and the identification of functional CREs.

The cotton genus, *Gossypium*, is well-established as a model for the study of evolutionary genomics of polyploidy. More than 50 species are known (Wendel and Grover 2015; Hu et al. 2021; Viot and Wendel 2023), and new cotton species continue to be discovered (Stewart et al. 2015; Gallagher et al. 2017). Phylogenetic analyses (Wendel and Cronn 2003; Wendel et al. 2010; Chen et al. 2017) and genome sequence data (Huang et al. 2021) indicate that the genus originated ∼5 to 10 million years ago (mya). Allopolyploid cottons (AD genome) originated in the Pleistocene following *trans*-oceanic dispersal of an A-genome progenitor to the New World, where it hybridized with a native D-genome diploid. Allopolyploids subsequently diversified into lineages now represented by seven species, including the commercially important *Gossypium hirsutum* (Upland cotton) and *G. barbadense* (Sea Island cotton), each domesticated within the last 7,000 years (Wendel and Grover 2015). The closest extant species related to the D-genome progenitor is *Gossypium raimondii*, whereas the two A-genome species, *G. arboreum* and *G. herbaceum*, are equally good models of the female (seed) parent in the initial hybridization (Wendel et al. 1989). This well-understood evolutionary history of *Gossypium* renders it an excellent model for studying allopolyploidy.

Previous studies have highlighted several aspects of duplicate gene expression evolution in *Gossypium*, including “homoeolog expression bias” (HEB), whereby one of the two homoeologs is more highly expressed than the other, and “expression level dominance” (ELD), an enigmatic phenomenon whereby the total expression of both homoeologs is statistically indistinguishable from the expression level of only one of the two parents (Rapp et al. 2009; Grover et al. 2012; Hu et al. 2013, 2014, 2015; Yoo et al. 2013; Gallagher et al. 2020). *Cis-* and *trans*-regulatory control of expression have also been studied in allopolyploid cotton, with *trans-*regulatory variants preferentially accumulating during about 5000 to 8000 years of domestication (Bao et al. 2019). These and other regulatory changes in cotton are associated with or causally connected to aspects of the chromatin landscape, including DNA methylation (Song et al. 2017), histone modification (Zheng et al. 2016), chromatin accessibility (Han et al. 2022), and 3D genomic topology (Wang et al. 2018), but to date, the molecular mechanisms underlying chromatin remodeling and its impact on duplicate gene expression remains largely unknown.

Here, we applied DNS-seq to comprehensively profile genome-wide chromatin accessibility and nucleosome organization in allopolyploid cotton *G. hirsutum*, relative to its model diploid progenitors and a synthetic, diploid F_1_ hybrid that mimics the natural hybridization that occurred 1 to 2 mya. In addition to characterizing the dynamics of chromatin structure change accompanying genomic merger and doubling, we also examined duplicated gene expression patterns to unravel the connections between chromatin remodeling and gene regulation in allopolyploid cotton. Taken together, our study provides a detailed view of the evolutionary dynamics of chromatin structure and *cis*-regulatory landscapes, highlights how these are altered by genome merger and doubling, and sheds light on their regulatory roles in duplicated gene expression evolution.

## Materials and Methods

### Plant Materials

Four *Gossypium* genotypes were used, including a natural allopolyploid (AD genome), *G. hirsutum* cultivar Acala Maxxa (AD_1_), and its model (A- and D-genome) diploid progenitors, i.e. *G. arboreum* accession A_2_-101 (A_2_) and *G. raimondii* (D_5_). The two diploid genome groups, A and D, last shared a common ancestor 5 to 10 mya (Wendel and Albert 1992), and have diverged to the extent that genome sizes (GSs) differ 2-fold. Thus, the corresponding interspecific diploid F_1_ hybrid (A_2_ × D_5_) was included to study the immediate consequences of the merger to two diverged genomes (in the absence of genome doubling and evolutionary time since polyploidization). Four to five plants per genotype were grown in the Bessey Hall Greenhouse at Iowa State University (Ames, IA, USA) under controlled short-day conditions (10 h photoperiod with darkness from 5 PM to 7 AM; 22/28°C, night/day). Mature leaf tissue was harvested from flowering branches at 5 PM, and immediately flash frozen in liquid nitrogen and stored at −80 °C.

### DNS-seq Experiment and Data Preprocessing

#### Nuclei Isolation

Nuclei were isolated using a modified protocol from Vera et al (2014). Briefly, four grams of frozen tissue were ground together with 10% (w/w) of polyvinylpolypyrrolidone under liquid nitrogen using a mortar and pestle, immediately followed by formaldehyde cross-linking for 10 min (min) in 40 mL fixation buffer (1.0 M 2-methyl-2,4-pentanediol, 10 mM PIPES⋅NaOH at pH 7.0, 10 mM MgCl_2_, 2% polyvinylpyrrolidone, 10 mM sodium metabisulfite, 5 mM β-mercaptoethanol, 0.5% sodium diethyldithiocarbamate trihydrate, 200 mM L-lysine, and 6 mM EGTA at pH 7.0) containing 1% formaldehyde. Fixation was stopped by adding 2 mL of 2.5 M glycine and stirring for 5 min. To degrade and solubilize organelles, 4 mL of 10% Triton X-100 was added to suspension, followed by stirring for 10 min. The suspension was filtered through one layer of Miracloth (Calbiochem) twice and placed in 50 mL centrifuge tubes. Nuclei were pelleted by centrifugation at 2,000 × *g* for 15 min at 4 °C and subsequently washed three times in 40 mL wash buffer (0.5 M 2-methyl-2,4-pentanediol, 10 mM PIPES⋅NaOH at pH 7.0, 10 mM MgCl_2_, 0.5% Triton X-100, 10 mM sodium metabisulfite, 5 mM β-mercaptoethanol, 200 mM L-lysine, and 6 mM EGTA at pH 7.0).

#### MNase Digestion and DNA Extraction

Nuclei pellets were resuspended in 2 mL MNase digestion buffer (50 mM HEPES at pH 7.6, 12.5% glycerol, 25 mM KCl, 4 mM MgCl_2_, and 1 mM CaCl_2_) and distributed into 500 μL aliquots. Different levels of nuclei digestion were conducted using either 5.6 U/mL (heavy) or 0.4 U/mL (light) MNase, both of which were incubated at 37 °C for 10 min. Digestion was stopped by adding 50 mM EGTA on ice for 5 min. Digested nuclei were de-cross-linked at 65 °C overnight in the presence of 1% SDS and 100 μg/mL proteinase K, and then treated with 40 μg/mL DNase-free RNaseA at 37 °C for an hour. DNA was extracted by phenol–chloroform extraction and precipitated with ethanol. Extracted DNA was electrophoresed on a 2% agarose gel to inspect the MNase digestion ladders. DNA fragments smaller than 200 bp were purified with the Axygen AxyPrep Mag PCR Clean-up Kit (Fisher Scientific), following a double-sided SPRI bead size selection (0.9× followed by 1.1×).

#### Library Preparation and Sequencing

DNA concentration was measured using the Qubit DNA Assay Kit with a Qubit 2.0 Fluorometer (Life Technology). Sixteen DNA sequencing libraries were prepared using the NEBNext Ultra DNA Library Prep Kit for Illumina (NEB), according to manufacturer instructions. Indexed libraries were pooled and sequenced on ten Illumina HiSeq 2500 lanes with paired-end 150-cycle sequencing.

#### Data Processing

After quality filtering and trimming of adaptor sequences using CutAdapt (Martin 2011), paired-end reads generated from the different *Gossypium* species were mapped against their corresponding reference genomes downloaded from CottonGen (Yu et al. 2014), including *G. hirsutum* cv. TM1 UTX v2.1 (Chen et al. 2020), *G. arboreum* cv. SXY1 WHU-updated v1.0 (Huang et al. 2020) and *G. raimondii* JGI v2.0 (Paterson et al. 2012). The F_1_ hybrid was mapped against a combined reference of *G. arboreum* and *G. raimondii.* Following Bowtie2 (v2.5.1) mapping with options “no-mixed,” “no-discordant,” “no-unal,” and “dovetail” (Langmead and Salzberg 2012), alignments of quality score ≥20 were retained for following analyses. Based on mapping read coverage, the deepTools (v2.5.2) (Ramírez et al. 2014) commands *plotCorrelation* and *plotPCA* were used to assess the reproducibility between replicates and the clustering of different MNase experiments; *computeMatrix* and *plotHeatmap* were used to visualize signal aggregation over genomic regions of interest, e.g. transcription start sites (TSSs) and transcription termination sites (TTSs). Read coverage data were converted to bigWig files using the UCSC Genome Bioinformatics utility (https://github.com/ucscGenomeBrowser/kent) code “bedGraphToBigWig,” and visualized on the Broad Institute Integrative Genomics Viewer (IGV) (Robinson et al. 2011).

### Nucleosome Calling Classification and Prediction

From the heavy MNase digestion, filtered MNase-seq read alignments were imported in R/Bioconductor framework version 3.5.0 and analyzed using the package nucleR (Flores and Orozco 2011). Paired-end reads under 260 bp were trimmed to 50 bp around the DNA fragment center. Genome-wide coverage in reads per million (RPM) was computed and normalized using the total number of read alignments from each sample. Noise filtering and peak calling were performed using the following nucleR parameters: pcKeepComp = 0.02, peak width = 147 bp, peak detection threshold = 35%, minimal overlap = 50 bp. If the identified peak width is above 150 bp, this peak is considered to contain more than two overlapped nucleosome dyads. Among the nonoverlapped nucleosome calls with peak width below 150 bp, well-positioned (W) nucleosomes were defined with peak height score above 0.6 and peak width score above 0.4, while the rest were classified as weakly positioned, or fuzzy (F) nucleosomes. Nucleosome coverage (NC) is defined as the percentage of genomic regions being occupied by nucleosomes. Nucleosome repeat length (NRL) is defined as the length of DNA wrapped around the histone octamer plus linker DNA, or the center-to-center distance between consecutive nucleosomes, which were estimated using NucTools scripts “nucleosome_repeat_length.pl” and “plotNRL.R” (Vainshtein et al. 2017). The R package NuPoP (Xi et al. 2010) was used for nucleosome positioning prediction from genomic DNA sequence, which explicitly models the linker DNA length with either a fourth-order or first-order hidden Markov chain. NuPoP outputs the Viterbi prediction of optimal nucleosome position map, based on which the predicted NC and NRL values were calculated.

### Mapping Accessible Chromatin Regions by DNS-seq

#### MNase Sensitive Footprints

Given the high level of reproducibility (Pearson's *r* > 0.9), mapping results from the two biological and technical replicates per MNase digestion and per genotype were pooled to generate the DNS profile for each genotype. Using a differential MNase-seq data processing pipeline previously established (Turpin et al. 2018), sequential computation steps were performed to (i) normalize the mapping read coverage in RPM between light and heavy MNase digestions, (ii) calculate DNS scores as the difference from light minus heavy read coverages, (iii) produce genome-browser-ready data tracks, and (iv) identify positive (MNase sensitive) and negative (MNase resistant) peaks using the genomic segmentation algorithm, iSeg (v1.3.4) (Girimurugan et al. 2018). To enable comparisons between species and (sub)genomes, an additional step of quantile normalization was performed before iSeg, normalizing the genome-wide DNS scores across diploid genomes (A_2_ and D_5_) and subgenomes (At and Dt) in hybrid and tetraploid cottons. A range of biological cutoff (BC) stringencies were tested in calling the MSFs and MNase resistant footprints (MRFs), represented by positive and negative DNS peaks, respectively, as previously termed (Vera et al. 2014). An optimized stringency BC = 6.0 was used (supplementary text 1, Supplementary Material online) to generate the final list of MSFs.

#### Subnucleosomal Particle Occupancy

As previously reported (Grossman et al. 2018; Savadel et al. 2021), small sequence fragments (0 to 130 bp) from the light MNase digestion can also be used to directly profile the occupancy of subnucleosome sized particles involved in transcriptional control. Using awk and BEDTools (v2.27.1) (Quinlan 2014), the geometric center of each small alignment (0 to 130 bp) from the light digestion was extracted and intersected with 21 bp sliding genomic windows with a step size of 5 bp. The smoothed profile of small fragment centers was normalized in RPM as the genome-wide subnucleosomal particle occupancy (SPO) scores. Different from the relative scores of DNS, quantile normalization of SPO scores across genomes would lead to substantial signal loss, so the resulting BedGraph files per genome were subjected to iSeg (v1.3.4) separately using optimized stringencies (supplementary text 1, Supplementary Material online). The resulting list of segments represents accessible chromatin regions (ACRs) identified by SPO.

### Mapping ACRs by ATAC-seq and DNase-seq

#### ATAC-seq

Two replicated ATAC-seq experiments were conducted using the young leaf tissue of *G. raimondii*, following a protocol described previously (Lu et al. 2017). For each replicate, approximately 200 mg freshly collected leaves or flash-frozen leaves were immediately chopped with a razor blade in 1 mL of prechilled lysis buffer (15 mM Tris–HCl pH 7.5, 20 mM NaCl, 80 mM KCl, 0.5 mM spermine, 5 mM 2-mercaptoethanol, 0.2% Triton X-100). The chopped slurry was filtered twice through miracloth and once through a 40 μm filter. The crude nuclei were stained with DAPI and loaded into a flow cytometer (Beckman Coulter MoFlo XDP). Nuclei were purified by flow sorting and washed in accordance with Lu et al. (2017). Sorted nuclei were incubated with 2 μL Tn5 transposase in a 40 μL tagmentation buffer (10 mM TAPS-NaOH pH 8.0, 5 mM MgCl_2_) at 37 °C for 30 min without rotation. Integration products were purified using a Qiagen MinElute PCR Purification Kit or NEB Monarch DNA Clean-up Kit and then amplified using Phusion DNA polymerase for 10 to 13 cycles. PCR cycles were determined as described previously (Buenrostro et al. 2013). Amplified libraries were purified with AMPure beads to remove primers. ATAC-seq libraries were sequenced in paired-end 35 bp at the University of Georgia Genomics & Bioinformatics Core using an Illumina NextSeq 500 instrument.

#### DNase-seq

Public data from cotton young leaves were previously reported (Wang et al. 2017, 2018; Han et al. 2022) and downloaded from NCBI (supplementary table S1, Supplementary Material online).

#### Data Processing

Raw ATAC-seq and DNase-seq reads were adapter and quality trimmed, and then filtered using “Trim Galore” (v0.4.5) (Krueger 2012). Clean reads were subsequently aligned to corresponding reference genomes using Bowtie2 (v2.3.4) (Langmead and Salzberg 2012) with the parameters “--no-mixed --no-discordant --no-unal --dovetail”. Three different sets of peak calling methods were tested for ATAC-seq as follows (supplementary text 2, Supplementary Material online), and the MACS2 method was used for DNase-seq.

#### HOMER and MACS2 Peak Calling

Duplicate reads were removed using Picard (v2.17.0) with default parameters (http://broadinstitute.github.io/picard/). Only uniquely mapped read pairs with a quality score of at least 20 were kept for peak calling. Phantompeakqualtools (v1.14) (Landt et al. 2012) was used to calculate the strand cross-correlation, and deepTools (v2.5.2) (Ramírez et al. 2016) was used to calculate correlation between replicates. The peak calling tool from HOMER (v4.10) (Heinz et al. 2010), i.e. *findpeaks*, was run in “region” mode and with the minimal distance between peaks set to 150 bp. MACS2 (v2.1.1) (Zhang et al. 2008) *callpeak*, a second peak calling algorithm, was run with the parameter “-f BAMPE” to analyze only properly paired alignments, and putative peaks were filtered using default settings and false discovery rate (FDR) < 0.05. Due to the high level of mapping reproducibility by deepTools (Pearson's correlation *r* = 0.99 and Spearman correlation *r* = 0.77), peaks were combined and merged between replicates for each tool using BEDTools (v2.27.1) (Quinlan 2014). BEDTools was also used to intersect HOMER peaks and MACS2 peaks to only retain peak regions identified by both tools as ATAC ACRs for subsequent analyses.

#### Genrich Peak Calling

Postalignment steps and peak calling for multiple replicates collectively were performed with one command using Genrich (v0.6.1) (https://github.com/jsh58/Genrich), which was developed and extensively tested in the Harvard FAS Informatics group. The alignment files from both replicates were collectively analyzed by Genrich with the options to remove PCR duplicates (-r), keep unpaired alignments by extending to the average fragment length (-x), exclude problematic genomic regions (-E blacklist.bed), and call peaks using a maximum *q*-value of 0.05 (-q 0.05) and a minimum AUC of 20.0 (-a 20.0). The output file produced by Genrich is in ENCODE narrowPeak format, listing the genomic coordinates, peak summit, and various statistics for each identified peak.

### ACR Characterization

#### Genomic Annotation

Various sources of ACRs were identified as described above, including MSFs, SPO regions, and ATAC-seq peaks. An additional filtering step was applied to remove a blacklisted region in *G. raimondii* (supplementary text 3, Supplementary Material online). According to proximity to the nearest genes, these ACRs were categorized as genic (gACRs; overlapping a gene), proximal (pACRs; within 2 kb of a gene), or distal (dACRs; >2 kb from a gene). To compare GC content between ACRs and nonaccessible genomic regions, the BEDTools *shuffle* command was used to generate the distal (by excluding genic and 2 kb flanking regions) and genic/proximal control regions (by including genic and 2 kb flanking regions), and the *nuc* command was used to calculate GC content for each ACR and permuted control regions. Using R package ChIPseeker (v1.18.0) (Yu et al. 2015), gACRs and pACRs were combined and further annotated into the following subcategories: promoter (<1 kb, 1 to 2 kb, 2 to 3 kb), exon, intron, downstream (<1 kb, 1 to 2 kb, 2 to 3 kb), and intergenic regions (>3 kb upstream from TSS and >3 kb downstream from TTS).

#### Relative to Transposable Elements

Whole-genome transposable element (TE) annotation was performed for all reference genomes using the EDTA (v1.9.5) (Ou et al. 2019) pipeline. The proportion of ACR within various TE superfamilies was calculated when the ACR coordinates intersect with a TE interval. Random control regions (of the same number, interval width, and composition of distal and genic/proximal regions as ACRs) were simulated using the BEDTools *shuffle* command to represent background noise, and the enrichment of ACR within each TE superfamily was assessed against the null distribution of control proportions based on permutation tests (*n* = 1000). Enrichment scores were calculated as the log_2_-transformed fold changes of observed versus the permutation-derived mean ACR proportions within TE superfamilies.

#### Differential Accessibility Analysis

Differences in chromatin accessibility attributable to hybridization and allopolyploidization were detected following an established differential accessibility (DA) workflow (Reske et al. 2020) using the R package *csaw* (v1.16.1) (Lun and Smyth 2016). For direct comparison between different cotton species, all MNase-seq data were aligned to the same reference genome, either the AD_1_ reference genome or a concatenated reference of A_2_ and D_5_ genomes; DA results derived from both references were examined to mitigate bias. Mapped and quality-filtered read pairs were counted into sliding windows or a given peak set to quantify MNase signals across the genome, followed by normalization based on the TMM or Loess method; multiple analytic approaches were evaluated to identify the most suitable DA workflow (supplementary text 4, Supplementary Material online). The resulting count matrices were then subject to the *edgeR* (Robinson et al. 2010) statistical framework of estimating dispersions by empirical Bayes and quasi-likelihood GLM fitting for hypothesis testing, according to the following designs: (i) light versus heavy in diploids; (ii) light versus heavy in F_1_; (iii) light versus heavy in AD_1_; (iv) F_1_:light–heavy versus diploids:light–heavy, representing hybridization effect; and (v) AD_1_:light–heavy versus F_1_:light–heavy, representing polyploidization effect.

### Motif Discovery and Enrichment Analysis

Using the MEME Suite (v5.4.1) (Bailey et al. 2015) with default settings, scanning for known motif occurrences in the 1 kb promoter regions was conducted with FIMO (Grant et al. 2011), and combined motif discovery and enrichment analysis was performed using both XSTREME (Grant and Bailey 2021) and AME (McLeay and Bailey 2010). XSTREME conducts two types of de novo motif discovery using MEME and STREME followed by enrichment analysis using SEA (Bailey and Grant 2021), and AME identifies known motifs that are relatively enriched in given sequences compared with control sequences. The promoter (<1 kb) ACRs per (sub)genome and corresponding promoter sequences were used as input and control sequences, respectively. The JASPAR core nonredundant plant motifs v2018 and Arabidopsis motifs from plantTFDB v5.0 (Jin et al. 2017) were used as known functional motifs. For clustering enriched motifs, the RSAT matrix-clustering tool (Castro-Mondragon et al. 2017) was used with the following parameters: *-hclust_method average -calc sum -metric_build_tree Ncor -lth w 5 -lth cor 0.6 -lth Ncor 0.4 -quick*. Heatmaps and hierarchical clustering were generated with Euclidean distance using the R package pheatmap (Kolde 2019).

### RNA-seq Analysis

Total RNA extractions were performed using the Sigma spectrum plant total RNA kit (Cat No. STRN50), and quantified on a BioAnalyzer (Agilent, Palo Alto, CA). mRNA libraries were prepared using the Illumina TruSeq RNA Library Prep Kit (Illumina, San Diego, CA, USA) and sequenced on three Hiseq 4000 lanes with paired-end 150-cycle sequencing. A total of 12 libraries from A_2_, D_5_, F_1_, and AD_1_ samples were generated with an average of 11 million read pairs per sample (supplementary table S1, Supplementary Material online). After quality filtering and trimming of adaptor sequences with TrimGalore (Krueger 2012), paired-end reads were pseudo-aligned to the reference transcriptomes using Kallisto (Bray et al. 2016). Under the R environment version 3.5.0, differential gene expression analysis was conducted using DESeq2 (Love et al. 2014), with an FDR *α* < 0.05 required to identify significant changes.

To optimize the method to infer duplicated gene expression patterns, we tested the following mapping strategies. (i) *D_5_-ref*: The *G. raimondii* (D_5_) reference genome (Paterson et al. 2012) and a previously generated species-diagnostic SNP index (Page et al. 2013) were used to construct the reference transcript sequences for Kallisto mapping of RNA-seq data from each genotype. For this reference, D_5_ reads were mapped to the D_5_ transcripts; A_2_ reads were mapped to the “pseudo-A_2_” transcripts, which were generated by replacing species-diagnostic SNPs on the D_5_ gene models with A_2_-specific SNPs; F_1_ reads were mapped against a concatenation of the pseudo-A_2_ and D_5_ transcripts; and *G. hirsutum* (AD_1_) reads were mapped against a concatenation of pseudo-AD_1_-A_t_ and pseudo-AD_1_-D_t_ transcripts, which were similarly generated using AD_1_-specific SNPs. (ii) *AD_1_-ref*: reads from all species were individually mapped against the *G. hirsutum* (AD_1_) transcript sequences (Chen et al. 2020). (iii) *individual-ref*: F_1_ reads were mapped to the concatenated A_2_ (Huang et al. 2020) and D_5_ (Paterson et al. 2012) transcripts, while A_2_, D_5_, and AD_1_ reads were each mapped to transcripts from their individual reference genomes. The resulting read counts from different references were compared based on syntenic ortholog/homoeolog relationships within the allopolyploid genome and between different references (i.e. A_2_, D_5_, F_1_:At, F_1_:Dt, AD_1_:At, and AD_1_:Dt), which were inferred using the pSONIC pipeline (Conover et al. 2021) as previously described (Conover and Wendel 2022).

Based on the *total* (summed) expression of At and Dt homoeologs, F_1_ and AD_1_ gene expression was compared to expression in A_2_ and D_5_ and subsequently classified into following categories (Rapp et al. 2009): (i) additivity, whereby the *total* expression (in the hybrid or allopolyploid) is statistically equivalent to the mid-parent value of the parental diploids; (ii) A-genome ELD, whereby the *total* expression is statistically equivalent to the A_2_ parent but different from the D_5_ parent and mid-parent expression; (iii) D-genome ELD, whereby the *total* expression is statistically equivalent to the D_5_ parent but different from the A_2_ parent and mid-parent expression; (iv) transgressive up-regulation, whereby the *total* expression is greater than both A_2_ and D_5_; (v) transgressive down-regulation, whereby the *total* expression is less than both A_2_ and D_5_.

Based on the partitioned expression of At and Dt homoeologs (separately), HEB was assessed in the F_1_ and AD_1_ by evaluating differential expression between homoeologs (At and Dt). Categorization of *cis-* and *trans*-regulatory divergence was performed as reported previously (Bao et al. 2019), which measured the overall contributions of *cis* and *trans* variants by log_2_ ratios of A_2_ and D_5_ [***A*** = log_2_(A_2_/D_5_)], the *cis* effects by log_2_ ratios of their corresponding homoeologs [***B*** = log_2_(At/Dt)], and then obtained the *trans* effects by ***A*** minus ***B***. Based on the statistical significance of ***A***, ***B***, and ***A*** minus ***B***, six categories of regulatory evolution were characterized as illustrated in Fig. 6c. The evolutionary impact of hybridization (***Hr***), allopolyploidization (***Pr***), and genome doubling (***Wr***) was determined according to Hu and Wendel (2019) and as illustrated in Fig. 6c.

### Histone Gene Family Analysis

Histone protein sequences of *Arabidopsis thaliana* were retrieved from HistoneDB 2.0 (Draizen et al. 2016) and Probst et al. (2020), which were used as queries to search against cotton coding genes by BLASTP with e^−5^ as cutoff. Using the built-in functions of the Seaview version 5 software (Gouy et al. 2021), multiple sequences alignment was conducted using MUSCLE (v3.8.31) (Edgar 2004), and phylogenetic analyses were performed using neighbor joining (NJ) and maximal likelihood (ML) methods. NJ trees were constructed with the “Poisson correction” model and a bootstrap test of 1,000 replicates. ML trees were constructed using PhyML (v3.0) (Guindon et al. 2010) with the default “LG” model and 100 nonparametric bootstrap replicates. For each histone family, the average evolutionary divergence among family members was calculated in MEGA11 (Tamura et al. 2021) as the number of amino acid substitutions per site from averaging over all sequence pairs (i.e. *overall mean distance*), using the Poisson correction model with all ambiguous positions removed for each sequence pair (pairwise deletion option).

### Data and Code Availability

Data generated in this research are deposited in the NCBI short read archive: MNase-seq under PRJNA529909, ATAC-seq under PRJNA1018916, and RNA-seq under PRJNA529417. All data used are detailed in supplementary table S1, Supplementary Material online. Custom scripts are available at the following GitHub repository: https://wendellab.github.io/cottonMNase-seq/.

## Results

### Mapping Chromatin Landscapes by Differential Sensitivity MNase-seq

To characterize the genome-wide chromatin features and *cis*-regulatory landscapes, we performed MNase digestion of fixed chromatin in nuclei using two digestion conditions—heavy and light, titrated according to a previously established protocol (Vera et al. 2014). A total of 16 MNase-seq libraries were generated, consisting of two conditions for two biological replicates from four genotypes: the allopolyploid *G. hirsutum* cultivar Acala Maxxa (AD_1_; GS = 2.2 Gb), A-genome diploid *G. arboreum* accession A_2_-101 (A_2_; GS = 1.8 Gb), D-genome diploid *G. raimondii* (D_5_; GS = 0.8 Gb), and their synthetic F_1_ hybrid (A_2_ × D_5_; GS = 2.4 Gb). An average of 60 million mono-nucleosome DNA-sized fragments (i.e. 150 bp read pairs) was sequenced per 1 Gb GS per library, resulting in 591 million A_2_, 126 million D_5_, 549 million A_2_ × D_5_, and 685 million AD_1_ read pairs (supplementary table S2, Supplementary Material online). After adapter trimming and quality filtering, the remaining 91% to 98% of reads were mapped to their corresponding reference genomes. Interestingly, the proportion of high-quality alignments (*Q* > 20) was notably higher for the D_5_ reads (80% to 86%) versus the other genomes surveyed (range: 60% to 74%; supplementary table S2, Supplementary Material online), likely reflecting lower repetitive content of the smaller D_5_ genome. Quality evaluation of the mapping results indicates that genomic coverage profiles were highly correlated between biological replicates (*R*^2^ = 0.91 to 0.99); therefore, alignments from replicates (per species and per digestive condition) were combined in the following analyses.

**Fig. 1.**
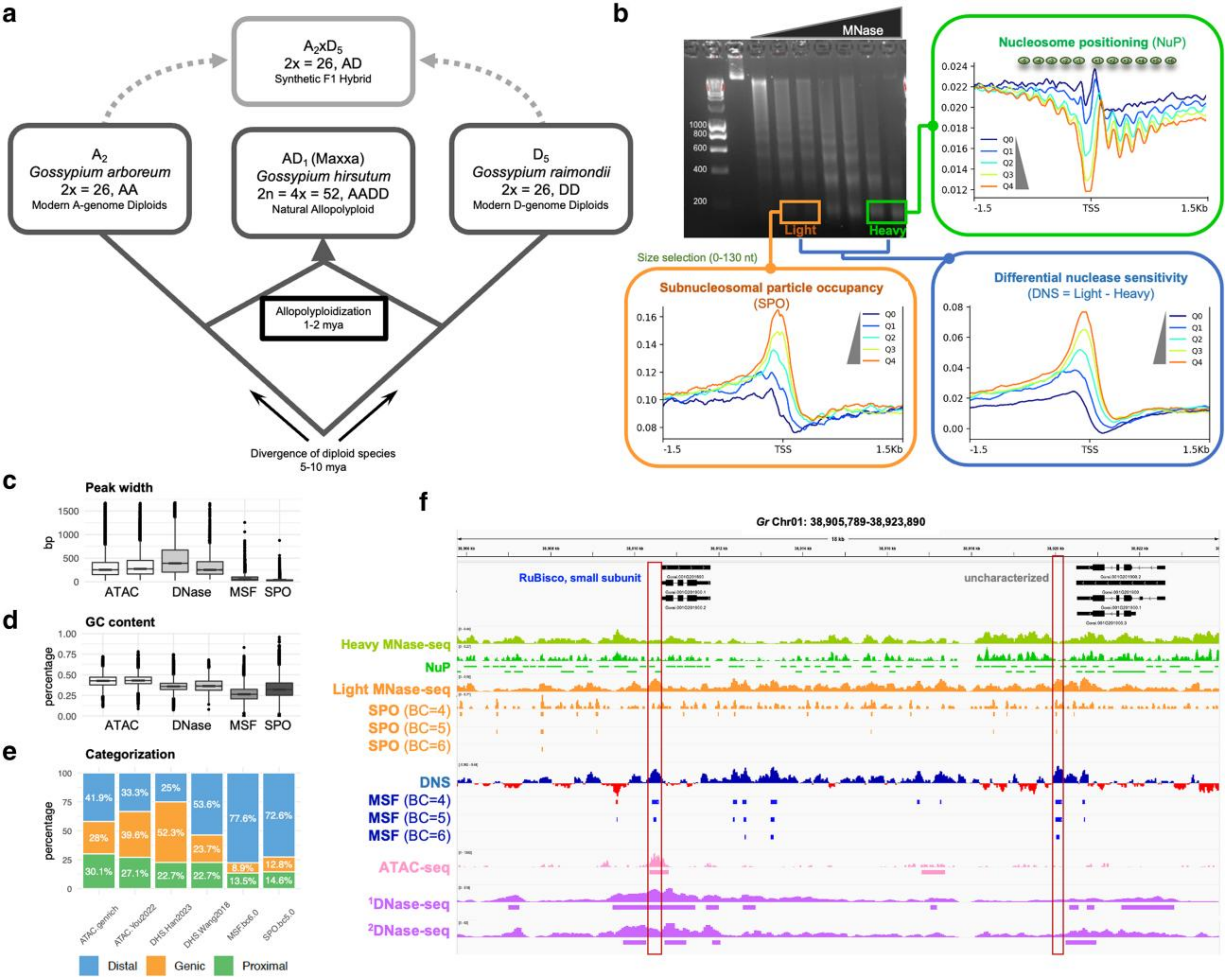
Studying chromatin structure evolution in diploid and allopolyploid cottons. a) Four *Gossypium* genotypes were used in this study: a natural allopolyploid, *G. hirsutum* cultivar Acala Maxxa (AD_1_); the model A- and D-genome diploid progenitors—*G. arboreum* accession A_2_-101 (A_2_) cultivar and *G. raimondii* (D_5_); and their corresponding interspecific diploid F_1_ hybrid (A_2_ × D_5_). b) The technique of DNS-seq was used to profile various chromatin features, including nucleosome positioning (NuP), SPO, and DNS. The agarose gel image shows nucleosomal DNA laddering from MNase digestions, where 5.6 U/mL and 0.4 U/mL were selected for heavy and light digestion, respectively. For each chromatin feature, aggregate plots are shown spanning ±1.5 kb around the TSS and binned by five gene expression level groups, where Q1 to Q4 represent increasing expression quantiles, and Q0 represents the group of nonexpressed genes. c to e). ACRs were compared between the analyses of MSF, SPO, ATAC-seq, and DNase-seq (see supplementary table S6, Supplementary Material online), in terms of peak width c), GC content d), and categorization relative to nearest genes e). Genic—ACRs are located within, or overlapped with, gene regions; Proximal—within 2 kb regions flanking genes; Distal—outside 2 kb regions flanking genes. f) A representative 18 kb region from D_5_ chromosome 1 shows a comparison of chromatin profiles by DNS-seq, ATAC-seq, and DNase-seq. Two leaf DNase-seq datasets were included: ^1^Han et al. (2022) and ^2^Wang et al. (2018). The gene *Gorai.001G201800*, encoding the small subunit of the chloroplast photosynthetic enzyme ribulose-1,5-bisphosphate carboxylase/oxygenase (Rubisco), was the most expressed gene in D_5_. Identified promoter ACRs are marked by boxes.

As illustrated in Fig. 1b (upper right), heavy digestion yields mainly mono-nucleosomes, as in traditional MNase-seq experiments, which enables genome-wide examination of nucleosome positioning and occupancy. The identification of well-positioned nucleosomes accounted for 16% to 20% of each *Gossypium* genome (supplementary table S3, Supplementary Material online), consistent with previous reports in human cells (Valouev et al. 2011) and plants (Wu et al. 2014; Zhang et al. 2015). The weakly positioned nucleosomes (or “fuzzy” nucleosomes), which accounts for 62% to 70% of each cotton genome, likely reflect positional variability and dynamics in multicellular samples. Around the TSS, the canonical pattern of nucleosome occupancy was observed: (i) the first nucleosome (+1 nucleosome) downstream of TSS is strongly localized, while array of phased nucleosome positioning gradually dissipates from the 5′ to the 3′ end of genes; (ii) the region immediately upstream of the TSS is generally depleted of nucleosomes, and thus called the “nucleosome-free region” (NFR), allowing access of TFs and other regulatory proteins; (iii) highly expressed genes tend to have a lower degree of nucleosome occupancy and a larger NFR (Fig. 1b).

The light MNase digestion releases more sensitive, “fragile” nucleosomes and subnucleosomal sized particles (e.g. TFs), which have been used to map MNase hypersensitive sites (MHSs) and profile chromatin accessibility as a complementary approach to DNase-seq and ATAC-seq (Pass et al. 2017; Parvathaneni et al. 2020; Zhao et al. 2020; Savadel et al. 2021). Here, the smaller DNA fragments (0 to 130 bp) sequenced from the light digestions were collected to identify open regions bound by subnucleosomal sized particles; while we refer to the corresponding genomic coverage from these light digestions as SPO (Fig. 1b, lower left), as per Teves and Henikoff (2011), these regions are sometimes referred to as “MHSs” (Zhao et al. 2020) or “MFs” for MOA-seq footprint (Savadel et al. 2021) regions. The DNS (Fig. 1b, lower right) approach permits the identification of MSFs that reveal *cis*-regulatory landscapes (Rodgers-Melnick et al. 2016; Parvathaneni et al. 2020). Thus, we took a combined approach of examining genome-wide chromatin profiles including nucleosome occupancy each by light and heavy digestion, SPO, and MSF, for comparative analyses of each *Gossypium* genotype studied (supplementary fig. S1, Supplementary Material online).

### Comparing Accessibility Analysis by DNS-seq with ATAC-seq and DNase-seq

To assess chromatin accessibility profiles obtained by DNS-seq, we compared our MSF and SPO results with independent datasets generated through different enzymatic assays, ATAC-seq and DNase-seq. Our comprehensive comparison included two replicated ATAC-seq experiments performed in this study (supplementary text 2, Supplementary Material online) and integrated publicly available ATAC-seq and DNase-seq data from three independent studies (Wang et al. 2018; Han et al. 2022; You et al. 2022), all focusing on young leaves of *G. raimondii* (supplementary table S1, Supplementary Material online). Applying recommended analyses of quality control metrics (Bubb and Deal 2020; Schmitz et al. 2022), we observed substantial variations across these datasets in sequencing depth (16.2 to 250.2 million read pairs per library), mapping rate (88.2% to 95.5%), duplication read rate (6.1% to 87.2%), and signal-to-background ratio (18.4% to 45.6%) (supplementary table S4, Supplementary Material online). The number of identified ACRs ranged from 2,059 to 59,763 (supplementary table S5, Supplementary Material online), with overlaps between datasets falling between 3% and 96% (supplementary table S6, Supplementary Material online). Such extensive variations highlight the potential influence of experimental methods and inherent challenges in chromatin accessibility assays for cotton, likely due to its high polyphenol and polysaccharide content. It is important to consider these factors when next comparing results from different techniques.

To explore the relationships between accessibility profiles mapped by different assays, we performed heatmap clustering of Pearson correlation coefficients and principal component analysis (PCA), which revealed distinct clusters for the genome-wide maps generated by DNS-seq, separated from those by ATAC-seq and DNase-seq (supplementary fig. S3, Supplementary Material online). Additionally, only a small fraction (<11%) of MSF and SPO regions were covered by ATAC-seq or DNase-seq peaks (supplementary table S6, Supplementary Material online), suggesting unique features captured by DNS-seq. Furthermore, the ACRs detected by DNS-seq exhibited several distinct characteristics compared to those identified by ATAC-seq and DNase-seq. Notably, MSF and SPO peaks were smaller, had lower GC content, and exhibited a more prominent distribution distal to genes (Fig. 1c to e; supplementary table S5, Supplementary Material online). These smaller ACRs with sharper signals are typically preferred for identifying *cis*-regulatory motifs (Savadel et al. 2021), and lower GC content has been shown to be more indicative of accessible *cis*-regulatory regions flanking genes (Gaffney et al. 2012; Ando et al. 2019; Weinberg-Shukron et al. 2022). Importantly, the accessibility profiles by MSF and SPO demonstrated the expected enrichment before the TSSs and depletion the gene bodies, showing a positive correlation with gene expression levels (supplementary fig. S1, Supplementary Material online), consistent with previous findings (Buenrostro et al. 2013). In contrast, the ATAC-seq and DNase-seq datasets often exhibited enrichment within gene bodies rather than before TSSs and lacked less robust correlations with gene expression (supplementary fig. S2, Supplementary Material online). These results suggest that DNS-seq offers a valuable approach for mapping chromatin accessibility with a strong link to gene expression levels in cotton.

In summary, the distinct clustering of genome-wide profiles (supplementary fig. S3, Supplementary Material online) and limited overlaps in ACRs (supplementary table S6, Supplementary Material online) highlight the unique perspectives provided by DNS-seq profiles, particularly in distant nongenic regions that appear to be less well represented in assays based on Tn5 or DNase I (Fig. 1e). This observation aligns with the previous studies of MHSs (equivalent to the SPO footprints here) in *Arabidopsis* (Zhao et al. 2020) and soybean (Fang et al. 2023), where a significant portion of MHSs (22% in *Arabidopsis* and 67% to 77% in soybean) were not detected by ATAC-seq or DNase-seq. The higher specificity observed here (89% of MSF and SPO peaks unique to DNS-seq) might be partially explained by the technical challenges and data quality issues associated with ATAC-seq and DNase-seq in cotton, as evidenced by the data quality variation (supplementary table S4, Supplementary Material online) and atypical ACR enrichment within gene bodies (supplementary fig. S2, Supplementary Material online). More importantly, these findings suggest that MNase-based approaches like DNS-seq hold significant promise for confident profiling of chromatin accessibility, particularly in resistant plants like cotton. This advantage likely stems from the utility of different MNase digestion conditions to derive reliable estimates of chromatin accessibility, in addition to the ability to capture MNase-specific sites in distal nongenic regulatory regions.

### Alteration of Nucleosome Organization by Hybridization and Allopolyploidization

**Fig. 2.**
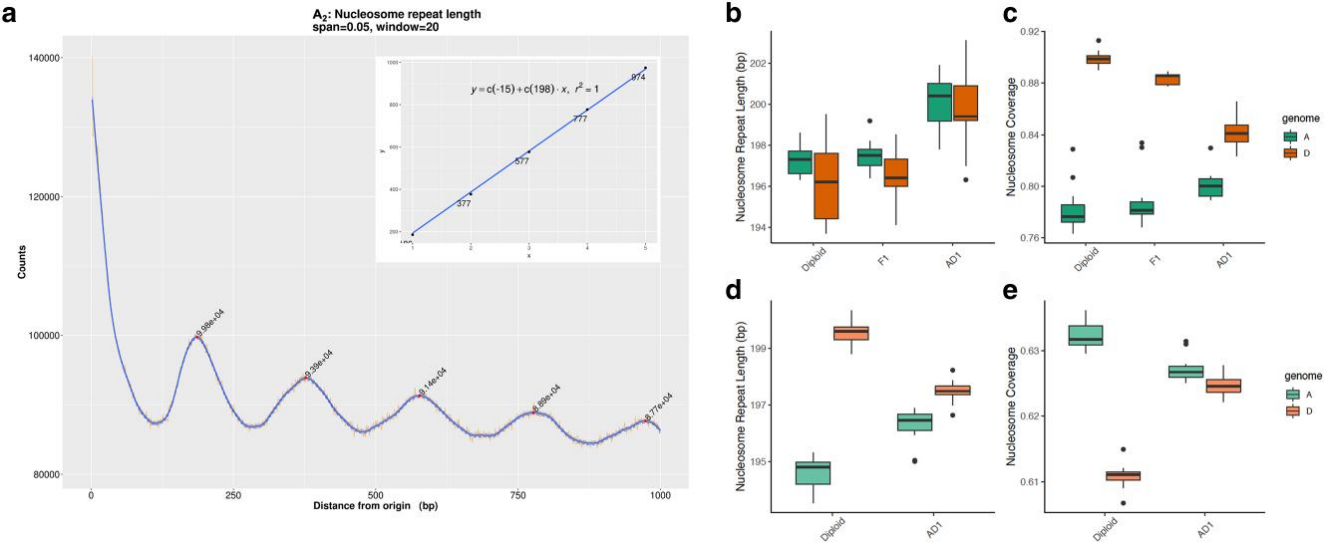
Comparing nucleosome organization in diploid, hybrid, and allopolyploid cottons. a) Nucleosome phasogram exhibits a wave-like pattern of distances between neighboring nucleosome centers. Inset presents a linear fit to the positions of the phase peaks, where the slope represents the estimated NRL of 198 bp in the exemplar, A_2_. b) Estimated NRL by phasogram across diploid and polyploid cotton genomes. c) Estimated NC based on the nucleosome positioning profiled by MNase-seq under heavy digestive conditions. d and e) Predicted NRL and NC based on reference genome sequence, respectively.

To compare nucleosome organization between diploid, hybrid, and allopolyploid cottons, we first computed phasograms to analyze the global patterns of nucleosome positioning and spacing. A phasogram represents the frequency distributions of distances between mononucleosomal reads mapped (i.e. from heavy MNase digestion), observed as oscillating sine wave signals, for which period is the center-to-center distance between neighboring nucleosomes, averaged genome wide (Valouev et al. 2011). For each cotton genome, the average distance between neighboring nucleosomes, also known as NRL, was estimated by applying a linear model to calculate the phasogram period (Fig. 2a; supplementary fig. S4 and table S7, Supplementary Material online). Interspecific and intergenomic comparisons revealed subtle but statistically significant genotype-based variation in average nucleosome spacing. We found that NRLs were generally shorter in the diploids and the diploid hybrid (F_1_) versus the allopolyploid cotton (AD_1_) and that the D-genome NRLs were generally shorter than those in the A-genome (Fig. 2b; Diploids: A_2_ 197.3 ± 0.2 bp, D_5_ 196.2 ± 0.5 bp; F_1_: At 197.5 ± 0.2 bp, Dt 196.4 ± 0.4 bp; AD_1_: At 200.1 ± 0.4 bp, Dt 199.7 ± 0.5 bp; ANOVA followed by Tukey's post hoc test, *P* < 0.05: diploids = F_1_ < AD_1_ and D < A). Consistent with these observations, the percentage of genomic regions occupied by nucleosomes (i.e. NC) also exhibited lower A- versus D-coverage, regardless of ploidy (Fig. 2c; Diploids: A_2_ 78.2 ± 0.5%, D_5_ 89.9 ± 0.1%; F_1_: At 78.8 ± 0.6%, Dt 90.0 ± 0.21%; AD_1_: At 80.1 ± 0.3%, Dt 84.1 ± 0.4%; A < D, Student's *t*-test *P* < 0.05). These results, i.e. shorter NRL and higher NC in the D-genome, together indicate that nucleosomes are generally arranged further apart in the larger A genome. Furthermore, both the NRL and NC reveal significantly larger interspecific differences between the A_2_ and D_5_ diploids relative to the intersubgenomic differences between At and Dt in the allopolyploid (AD_1_), which suggests that allopolyploidization and subsequent evolution as a tetraploid, but not hybridization per se, may result in homogenization of nucleosome density.

Nucleosome positioning is known to be directed by a combination of the intrinsic properties of DNA sequence that act in *cis* and chromatin remodeling that deploys transcription machinery that acts in *trans* (Radman-Livaja and Rando 2010). Therefore, we next examined the roles for *cis*- and *trans*-acting factors in changing the nucleosome distribution during genome evolution. To isolate the *cis* effects, we applied a sequence-based computational model to predict the “intrinsically DNA-encoded” nucleosome features. If each prediction agrees with the experimental estimation, we conclude that *cis* DNA sequence plays a significant role; otherwise, a significant *trans* effect would be inferred. Interestingly, sequence-based predictions of nucleosomal spacing and coverage for each reference genome (i.e. A_2_, D_5_, and AD_1_) suggest that the NRL should be shorter in the A-(sub)genomes (vs. the D-(sub)genomes) with a concomitantly higher NC value, regardless of ploidy level (Fig. 2d and e). This observation directly contrasts the experimentally observed pattern (Fig. 2b and c) and therefore implies a possible role for *trans* effects in nucleosome positioning. Given this observation, it is perhaps surprising that the sequence-based nucleosome positioning predictions for diploid versus polyploid cotton mirrored that of the MNase-seq estimations, both of which find that the differences in NRL and NC between the A_2_ and D_5_ diploids exhibit significant reductions in the At and Dt subgenomes of the allopolyploid (AD_1_). In other words, the synchronization effect on nucleosome organization was impacted in *cis* by sequence evolution accompanying allopolyploidization.

### Chromatin Accessibility Increases in Allopolyploid Promoters

**Fig. 3.**
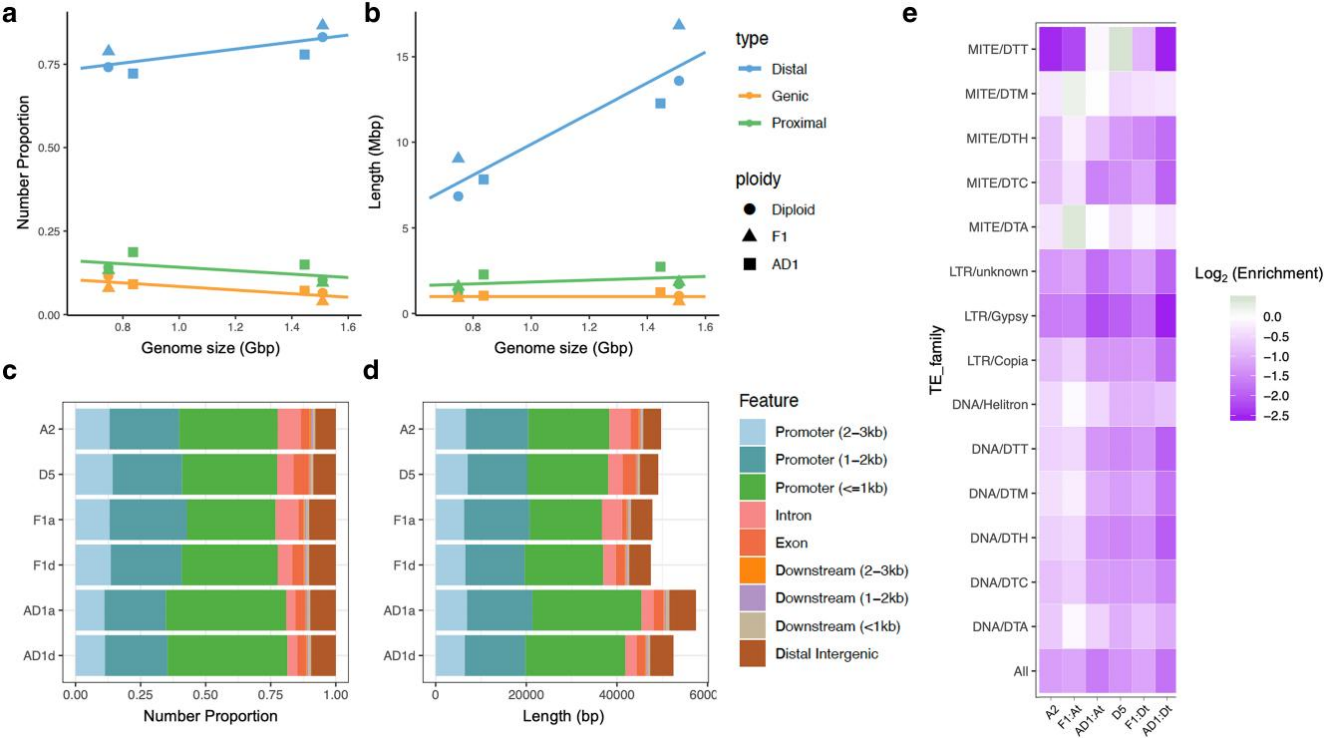
Comparing ACRs in diploid, hybrid, and allopolyploid cotton. a and b) ACRs were categorized as genic (gACRs), proximal (pACRs), or distal ACRs (dACRs). Their relative proportions a) and total lengths b) are presented (*y*-axis) against corresponding genome sizes (*x*-axis), with a linear regression trendline plotted per category. The reference genome sizes used are: A_2_ = F_1_:At = 1.51 Gb; D_5_ = F_1_:Dt = 0.75 Gb; AD_1_:At = 1.45 Gb; AD_1_:Dt = 0.84 Gb. c and d) Parsed categorization of gACRs and pACRs using detailed genomic annotations from ChIPseeker, displayed as peak proportions c) and total lengths d). ANOVA followed by Tukey's post hoc test found significant increases in both proportion and total length of pACRs within the AD_1_ 1 kb promoter regions (*P* < 0.05). e) Heatmap of ACR presence in TEs. Enrichment scores were calculated as the log_2_-transformed fold changes of observed versus expected (estimated from 1,000 permutations) mean ACR proportions within TE superfamilies.

ACRs were identified for each sample from the DNS and SPO data combined, comprising 1.1% to 1.4% of each genome (Table 1; supplementary tables S8 to S11, Supplementary Material online). In the F_1_ hybrid, we identified 581,654 ACRs covering 30.9 Mbp. Both the numbers and total genomic fractions of ACRs were higher than their combined counterparts in the diploid progenitors, A_2_ (296,312; 16.4 Mbp) and D_5_ (190,795; 9.2 Mbp). In the allopolyploid AD_1_, only the total length of ACRs (449,346; 27.4 Mbp) surpassed that of diploid progenitors. A majority of ACRs were located >2 kb from their nearest gene (distal, dACRs: 72% to 87%), whereas 10% to 19% occurred proximally within the 2 kb gene flanking regions (proximal, pACRs) and only 4% to 12% overlapped gene bodies (genic, gACRs). The larger A-(sub)genomes exhibited a higher proportion of dACRs and commensurately lower proportions of gACRs and pACRs relative to the smaller D-(sub)genomes (Fig. 3a), consistent with observations in other plant species which suggest that the proportion of dACRs is positively correlated with GS (Lu et al. 2019). This correlation with GS was even more significant for the total length of dACRs (Fig. 3b), whereas gACRs and pACRs were mostly comparable between A- and D-(sub)genomes, likely due to their general conservation in genes. Interestingly, the proportion and total length of pACRs were significantly increased in AD_1_, specifically due to expansions in the 1 kb promoter regions (Fig. 3c and d).

**Table 1:** ACR classification

	Diploid		F_1_		AD_1_	
	A_2_	D_5_	At	Dt	At	Dt
*Number*	296,312	190,795	357,171	224,483	260,278	189,068
In proximity to genes						
gACR	19,025 (6.4%)	22,240 (11.7%)	13,948 (3.9%)	17,754 (7.9%)	18,540 (7.1%)	17,151 (9.1%)
pACR	30,710 (10.4%)	26,881 (14.1%)	33,873 (9.5%)	29,719 (13.2%)	38,907 (14.9%)	35,333 (18.7%)
dACR	246,577 (83.2%)	141,674 (74.3%)	309,350 (86.6%)	177,010 (78.9%)	20,2831 (77.9%)	136,584 (72.2%)
Overlapped with TEs						
LTR retrotransposons	75,406 (25.4%)	24,306 (12.7%)	98,680 (27.6%)	36,251 (16.1%)	48,132 (18.5%)	20,486 (10.8%)
DNA transposons	19,161 (6.5%)	17,526 (9.2%)	26,249 (7.3%)	23.941 (10.7%)	16,658 (6.4%)	15,738 (8.3%)
Nonoverlapped with TEs	201,745 (68.1%)	148,963 (78.1%)	232,242 (65.0%)	164,291 (73.2%)	195,488 (75.1%)	152,844 (80.8%)
*Length (Mbp)*	16.4	9.2	19.3	11.5	16.2	11.1
In proximity to genes						
gACR	1.0 (6.3%)	1.0 (11.0%)	0.7 (3.6%)	0.9 (7.8%)	1.2 (7.6%)	1.0 (9.3%)
pACR	1.7 (10.6%)	1.3 (14.3%)	1.8 (9.5%)	1.6 (13.6%)	2.7 (16.8%)	2.3 (20.4%)
dACR	13.6 (83.1%)	6.9 (74.7%)	16.8 (86.9%)	9.0 (78.6%)	12.3 (75.6%)	7.8 (70.3%)
Overlapped with TEs						
LTR retrotransposons	4.2 (25.8%)	1.2 (13.0%)	5.4 (28.1%)	1.8 (16.0%)	2.7 (16.3%)	1.1 (9.8%)
DNA transposons	1.1 (6.7%)	0.9 (9.4%)	1.5 (7.8%)	1.2 (10.8%)	1.0 (6.2%)	0.9 (7.9%)
Nonoverlapped with TEs	11.1 (67.5%)	7.1 (77.6%)	12.4 (64.2%)	8.4 (73.2%)	12.6 (77.5%)	9.2 (82.4%)

**Fig. 4.**
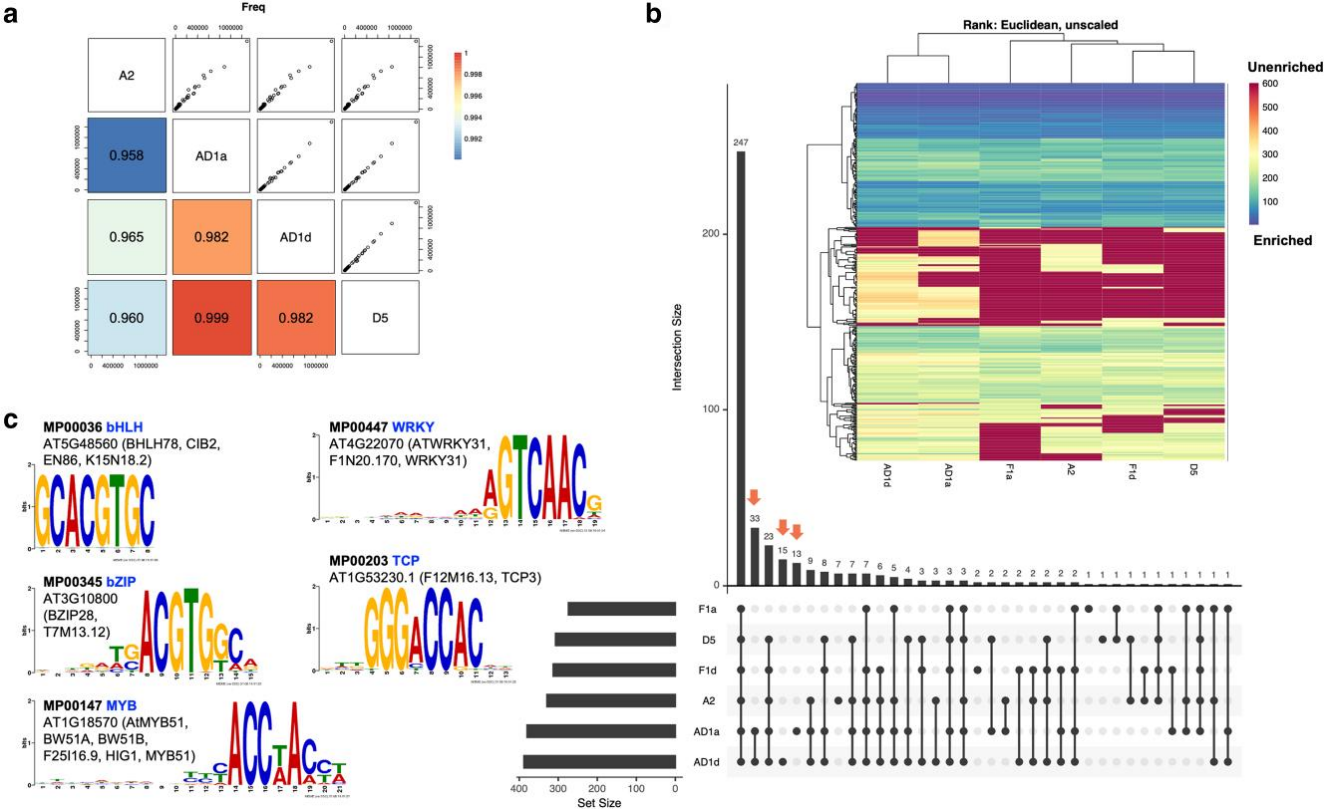
Motif analysis of pACRs in 1 kb promoters. a) Comparing background promoter sequences between genomes, based on FIMO scanning of their whole promoter region sequences for known motifs from plantTFDB v5.0 (Jin et al. 2017). Upper triangle of the matrix shows the scatter plots of motif frequency, and the lower triangle presents the pairwise Pearson correlation coefficients of motif frequency. b) A union of 423 enriched motifs within the 1 kb promoter pACRs by AME. The UpSetplot presents the intersection of motif sets, with AD_1_-specific sets marked by orange arrows. For each (sub)genome, the enrichment ranking of motifs was used for clustering and heatmap visualization, i.e. the lower ranking indicates more enrichments. A ranking score of 600 was assigned to unenriched motifs in the corresponding genome. c) Top 5 most enriched AD_1_-specific motifs.

For each genome, an initial scan of the 1 kb promoter sequences for known DNA motifs from plantTFDB v5.0 (Jin et al. 2017) revealed relatively consistent motif occurrences across (sub)genomes, although the A_2_ promoters exhibited the most divergence relative to the other genomes (Fig. 4a), possibly due to the elevated GC content in its promoters (A_2_ 30.56%, vs. AD1:At 28.08%, D_5_ 28.72%, AD_1_:Dt 28.98%). We then used this background variation in 1 kb promoter sequences as a control to obtain enriched motifs from the pACRs by AME, resulting in 351, 326, and 408 enriched motifs in the parental diploids (aggregated), the F_1_, and in AD_1_, respectively (supplementary table S12, Supplementary Material online). Among the union of 423 enriched motifs, 247 were shared by all (sub)genomes, indicating a high level of *cis*-element conservation among cotton (sub)genomes (Fig. 4b). Interestingly, AD_1_-specific motifs comprised the second, fourth, and fifth largest intersecting sets, which include 33 motifs enriched in both At and Dt pACRs, 15 enriched in Dt only, and 13 enriched in At only. These motifs mostly belong to TFBSs of MYB (ten motifs), WRKY (nine motifs), bZIP (nine motifs), and TCP (nine motifs) TF families (Fig. 4c; supplementary table S12, Supplementary Material online). Congruently, a heatmap dendrogram of pACR motif enrichment rankings showed that AD_1_:At and AD_1_:Dt were more similar to each other and distinct from the diploid enrichment rankings. Among the diploids, clustering of F_1_:Dt and D_5_ showed their higher similarity, with the A_2_ and F_1_:At genomes falling more basally in that clade (Fig. 4b). Furthermore, de novo motif discovery by XSTREME and clustering analysis (supplementary fig. S5, Supplementary Material online) confirmed these patterns, suggesting a synchronization effect associated with allopolyploidization and a potentially asymmetric effect associated with hybridization.

### Decreased Chromatin Accessibility in Repetitive Regions Accompanying Allopolyploidy

**Fig. 5.**
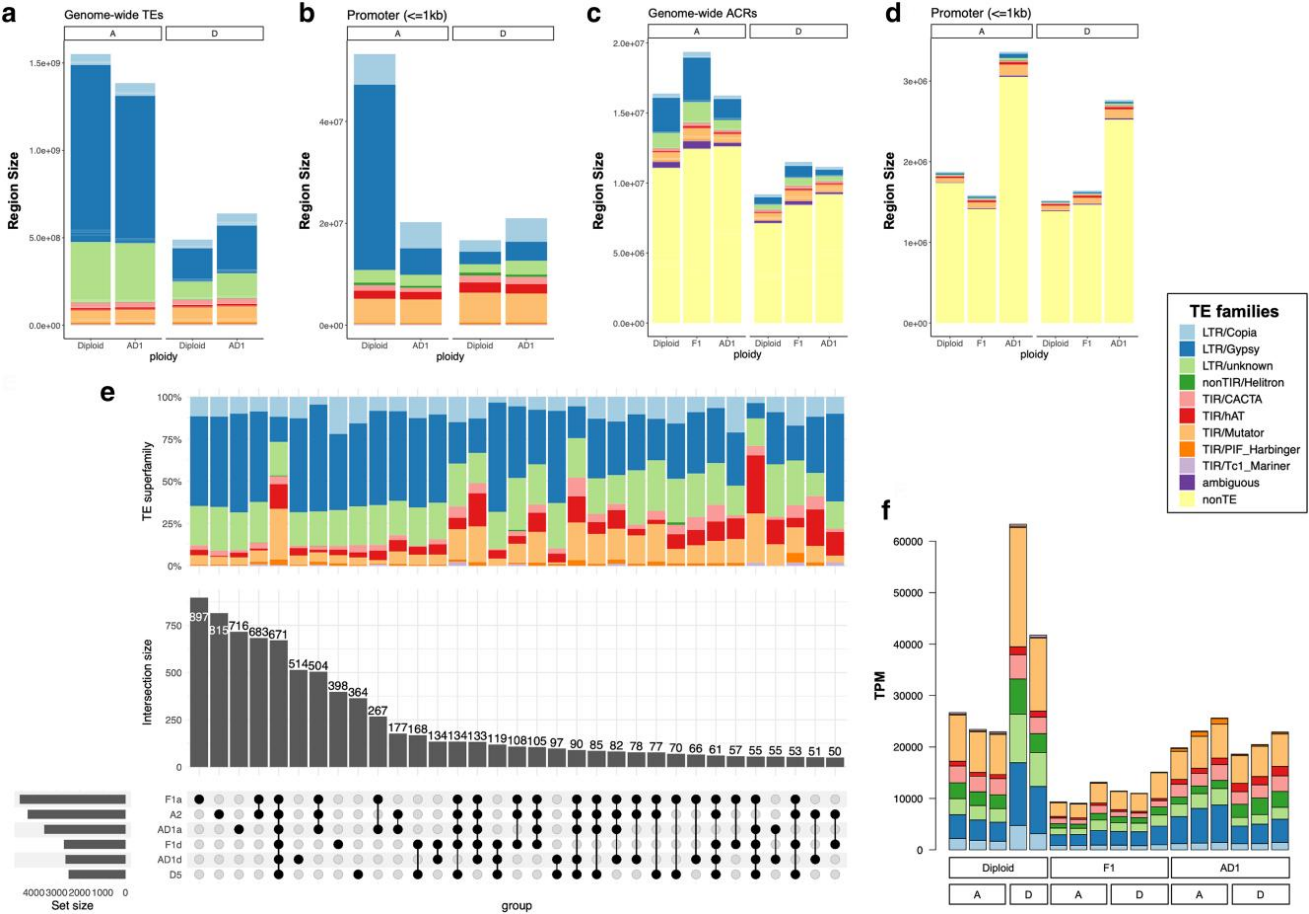
Chromatin accessibility and TEs. a and b) Sizes of TE superfamilies at genome-wide scale a) and within 1 kb promoters b). c and d) Contribution of TEs to ACRs at genome-wide scale a) and within 1 kb promoters b). ACRs that do not overlap with TEs were labeled as “non-TE.” ACRs that overlapped with TEs were considered TE-derived ACRs and further classified by TE superfamilies. e) A union of 8,680 TE families significantly contributed to ACRs. A hypergeometric *P* < 0.05 of TE and ACR overlapping was required to consider the contribution of TE families. UpSetplot presents intersecting TE families sets, with the proportion of superfamilies shown in the barplot above. f) TE expression measured in TPM reads using the cotton leaf transcriptome data.

Genome-wide characterization of TEs revealed that the A subgenome of AD_1_ has 1.2% lower TEs than A_2_ whereas the D subgenome has 5.1% more TEs than D_5_ (AD_1_:At = 81.2%, AD_1_:Dt = 64.6%, A_2_ = 82.4%, and D_5_ = 59.5%; Fig. 5a; supplementary table S13, Supplementary Material online), consistent with previous reports (Zhao et al. 1998; Chen et al. 2020). ACRs accounted for only 0.31% to 0.68% of genomic regions annotated as TEs, significantly lower than their composition in other genomic regions (1.09% to 1.37%; permutation test *P* < 0.05). Depletion of ACRs was evident for all TE superfamilies, with the greatest depletion detected for the Gypsy retrotransposons (Fig. 3e), as expected by their general tendency to reside in heterochromatic regions. More A- than D-(sub)genomic ACRs overlapped with TEs, particularly LTR retrotransposons, congruent with the higher TE content in the larger A-genome (Table 1). Regardless of subgenome, however, the allopolyploid (AD_1_) contained the lowest amounts of ACRs that overlapped with TEs (AD_1_: At 22.5%, Dt 17.6%; F_1_: At 35.8%, Dt 26.8%; A_2_ 32.5%; D_5_ 22.4%), indicating decreased chromatin accessibility in TE regions accompanying allopolyploidization.

Because the allopolyploid (AD_1_) exhibits both a reduction in TE-overlapping ACRs and an increase in promoter ACRs (Fig. 3d), we hypothesized that promoters may have gained more accessibility from TE removal associated with polyploidization. The general distribution of TEs around TSSs is similar between diploid and polyploid cottons (supplementary fig. S6, Supplementary Material online). However, the diploid A_2_ exhibits a strikingly high number of Gypsy elements within its 1 kb promoter regions that are absent from its homologous genome in the allopolyploid, and this pattern was not observed in other genomic regions (Fig. 5b; supplementary fig. S7 and table S14, Supplementary Material online). At the genome-wide scale, TEs contributed to 18% to 36% of ACRs (Table 1; Fig. 5c), but these accessible TEs were mainly located in distal intergenic regions and only contributed to a small portion of promoter ACRs (Fig. 5d). The DNA transposon Mutator-derived ACRs were most abundant within promoters, consistent with their genomic distribution and tendency to be near genes compared to the distribution pattern of LTR retrotransposons. Interestingly, the loss of Gypsy in AD_1_ promoters (Fig. 5b) is associated with a gain of both non-TE and TE-derived ACRs accompanying allopolyploidy (Fig. 5d). The percentage of TE-derived ACRs at the 1 kb promoter regions was significantly higher in AD_1_ than in diploids and F_1_ (AD_1_: At 0.86%, Dt 0.63%; F_1_: At 0.41%, Dt 0.47%; A_2_ 0.46%; D_5_ 0.36%; χ^2^ test *P* < 0.05). Although this observation supports our hypothesis that promoter TE depletion led to increased accessibility in the At genome of the allopolyploid (relative to A_2_), it does not explain the increased accessibility in the Dt genome (vs. D_5_).

Because TE superfamily distribution may vary among genomic regions, we asked whether any particular TE families represented a key source of ACRs. Although we observed a strong positive correlation between genome-wide TEs and TE-derived ACRs for superfamilies within each genome (AD_1_:At 0.96, AD_1_:Dt 0.84; F_1_:At 0.98, F_1_:Dt 0.96; A_2_ 0.99; and D_5_ 0.93), we did not observe ACR enrichment of particular TE superfamilies. Out of the 28,057 TE families characterized across cotton species, a union of 8,680 families was found significantly enriched in TE-derived ACRs (Fig. 5e). Intersection of TE families among genomes revealed a significant proportion of lineage-specific TE families, which accounts for 14% to 20% of the TE-overlapping ACRs in each (sub)genome. The largest intersection set of 897 families was only found in the At genome of the diploid synthetic hybrid. While these lineage-specific families are mainly LTR retrotransposons, TE families shared by at least half of the genomes tend to be depleted of Gypsy and enriched in Mutator and hAT.

Because TEs are often associated with both inaccessible chromatin and transcriptional repression, we evaluated the expression of accessible TEs using transcriptomic data. We found that TE-based transcripts from 7,045 TE families accounted for 3% to 6% of mapped RNA-seq reads. Notably, 4,622 of these expressed TE families also significantly contributed to ACRs (i.e. found among 8,680 families mentioned above). The significant overlap between transcription and accessibility (χ^2^ association test *P* < 0.05) indicates that accessible TEs are likely to be transcriptionally expressed. Interestingly, while the numbers of expressed TE families were comparable between cotton genomes (A_2_ 3622, D_5_ 2973, F_1_:At 3242, F_1_:Dt 3115, AD_1_:At 3381, AD_1_:Dt 2946), higher transcript abundances were found in D_5_ (Fig. 5f; supplementary fig. S8, Supplementary Material online).

### Allopolyploidy Causes More Accessibility Changes than Does Hybridization

Both interspecific hybridization and polyploidization can have profound effects on the epigenome and gene expression. To assess their effects on chromatin accessibility, we initially compared ACRs identified in the diploid A_2_ and D_5_ genomes (supplementary tables S8 and S9, Supplementary Material online) with their homologs in the At and Dt subgenomes of the interspecific diploid hybrid (F_1_) (supplementary table S11, Supplementary Material online). This comparison resulted in only 10.5% of diploid ACRs (11.0% of A_2_ and 9.7% of D_5_) overlapping with F_1_ ACRs. We note that the relatively low overlap in ACRs between diploids and F_1_ reflects the stringency of our peak calling method. Notably, as stringency increased, overlaps decreased, as detailed in supplementary text 1, Supplementary Material online. Given the stringent criteria applied independently for each genotype, the outcome of limited overlap was not entirely unexpected from this conservative approach.

To further validate our findings, we reanalyzed the DNase-seq diploid and F_1_ datasets reported by Han et al. (2022) for comparison (supplementary table S1, Supplementary Material online). This analysis detected 28,029 (8.7 Mbp), 59,763 (34.1 Mbp), and 36,467 (11.2 Mbp) differential hypersensitive sites (DHSs) from A_2_, D_5_, and F_1_, respectively. In comparison with the original report (77,915 in A_2_, 59,997 in D_5_, and 78,722 in F_1_), our analysis is over 2-fold more stringent, resulting in much lower overlap of 36.6% between diploids and F_1_ compared to the reported overlap of 83.7%. Additionally, much smaller ACRs were detected by DNS-seq compared to those identified by DNase-seq (mean peak width 54 bp vs. 492 bp), which naturally leads to reduced overlap upon intersection.

Recognizing these technical nuances, we employed a DA approach directly contrasting the MNase-seq data between diploid genomes and their corresponding subgenomes in the F_1_ and natural allopolyploid (see Materials and Methods and supplementary text 4, Supplementary Material online). Compared to ACR intersections, this method is more sensitive to capturing similarities between accessibility profiles and independent of the stringency of ACR peak calling. The DA analysis of allopolyploid versus diploids revealed an increase of 3.3 to 4.4 Mb and a decrease of 435 to 740 kb in accessibility. In contrast, the differences between the F_1_ hybrid and the diploids were smaller, showing an increase of 16 to 304 kb and a decrease of 132 to 214 kb. These results indicate that allopolyploidization and subsequent evolution at the allopolyploid level, for >1 to 2 million years in this case, collectively induce much greater changes in chromatin accessibility than does hybridization in the F_1_. The consequences of these accessibility changes, particularly in promoter regions, were explored next.

### Duplicated Gene Expression in Diploid Hybrid and Allopolyploid Cotton

**Fig. 6.**
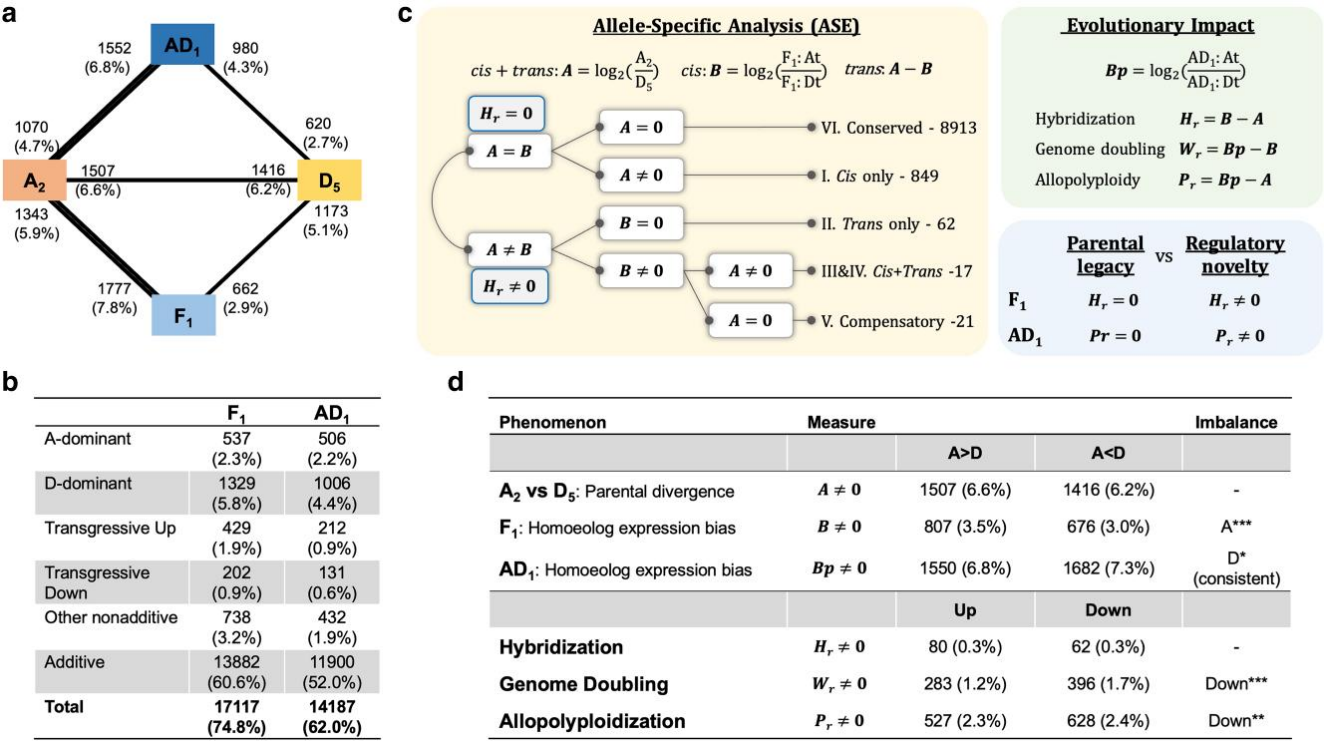
Duplicated gene expression patterns based only on the consistent results by different mapping strategies. a) Differential *total* expression of homoeologous genes in AD_1_ and F_1_ relative to A_2_ and D_5_ parental diploids. Between AD_1_ and A_2_, for example, 1,552 genes (6.8% of 22,889 ortholog groups) are more highly expressed in AD_1_, and 1,070 genes (4.7%) are more highly expressed in A_2_. The thicker lines relative to A_2_ than to D_5_ represented asymmetrically more expression changes to the A-genome parent. b) Test of the additivity hypothesis in AD_1_ and F_1_. Nonadditive expression categories include ELD (A-dominant and D-dominant), transgression (up- and down-regulation), and other nonadditive patterns. For 74.8% and 62.0% of 22,889 ortholog groups, the classification results were consistent by different mapping strategies. c) Illustration of the extended *cis* and *trans* analytic framework (Hu and Wendel 2019), which combined the classic ASE analysis with the estimation of evolutionary impact. d) Summary of parental divergence, HEB, and evolutionary impacts. χ^2^ tests were performed to infer the significance of imbalance: - as insignificant with *P* > 0.05; *P < 0.05; **P < 0.01; ***P < 0.001. Only for HEB in AD_1_, the significant imbalance was found consistent by different mapping strategies, thus labeled as robust in parenthesis.

To assess the consequences of chromatin changes on gene expression evolution, we first characterized the evolution of duplicated gene expression using matching RNA-seq data generated for the two diploids A_2_, D_5_, their F_1_ hybrid, and natural allopolyploid derivative, AD_1_ (supplementary table S1, Supplementary Material online). Duplicated gene expression patterns (Box 1) were categorized under a preestablished analytical framework (Hu and Wendel 2019), illustrated in Fig. 6. We employed a conservative approach and only report results that are consistent across different mapping strategies (supplementary tables S15 to S18 and text S5, Supplementary Material online). We also restricted our analysis to genes where orthology and homoeology among (sub)genomes could be confidently determined. For each of these 22,889 ortho-homoeolog groups (OGs; each containing a single representative for A_2_, D_5_, F_1_:At, F1:Dt, AD_1_:At, and AD_1_:Dt), duplicated gene expression patterns were characterized based on *total* (Fig. 6a and b) and partitioned homoeologous expression levels (Fig. 6c and d), including differential *total* expression relative to parental diploids, ELD, HEB, *cis-* and *trans*-regulatory divergence, as well as the evolutionary impact of hybridization (***Hr***), allopolyploidization (***Pr***), and genome doubling (***Wr***).

Box 1. **Terminology of duplicated gene expression patterns**

- Additive and nonadditive expression: A condition of allopolyploid or hybrid gene expression relative to parental expression levels. Additive expression is inferred when the *total* expression of homoeologous copies matches the arithmetic average of orthologous expression levels in parental diploids. Nonadditive expression deviates from this average, characterizing novel expression patterns specific to a hybrid or allopolyploid.
- ELD: A subcategory of nonadditive expression, where the *total* homoeologous expression is equivalent to the orthologous expression level of one parental diploid but not the other; in the present application, when the aggregate (At + Dt) expression level in the allopolyploid is statistically equivalent to the level of the A genome but not the D-genome diploid, the allopolyploid exhibits ELD toward the A-genome, or A-dominant expression. Note that ELD can be inferred for both higher and lower expression levels of one diploid relative to the other.
- Transgressive expression: Another subcategory of nonadditive expression, where the *total* homoeologous expression statistically exceeds or falls below both parental levels, termed transgressive up-regulation or transgressive down-regulation, respectively.
- HEB: Unequal expression of homoeologous gene copies of a given gene in allopolyploid or hybrid. The direction of bias is indicated by the homoeolog showing higher expression (e.g. At bias). When the bias direction toward a specific subgenome is preferentially observed *among* genes, the genome-wide HEB becomes “unbalanced.”
- *Cis-* and *trans-*regulatory divergence: The regulatory cause of homoeolog gene expression changes relative to parental states (e.g. At/Dt vs. A_2_/D_5_) can be decomposed into *cis-* and *trans-*acting components. *Cis-*regulatory variants refer to sequence changes in the *cis*-elements near genes, while *trans*-regulatory variants refer to expression or functional changes in *trans-*activating factors like TFs. The relative contributions of *cis* and *trans* variants can be estimated using an ASE framework as illustrated in Fig. 6b.
- Impact of genome evolution: The dynamic changes of relative expression patterns between duplicated genes (e.g. A_2_/D_5_ in diploids and At/Dt in hybrid and allopolyploid) can be decomposed into effects of hybridization (***Hr***), allopolyploidization (***Pr***), and genome doubling (***Wr***), according to Hu and Wendel (2019) and as formulated in Fig. 6c.

In both the F_1_ and AD_1_, the *total* expression of homoeologous genes exhibited more differential expression relative to A_2_ than to D_5_ (F_1_—13.7% vs. 8.0%; AD_1_—11.5% vs. 7.0%; Fig. 6a), and, correspondingly, the ELD analysis revealed more D-dominant than A-dominant expression patterns (F_1_—5.8% vs. 2.3%; AD_1_—4.4% vs. 2.2%; Fig. 6b). These observations suggest an asymmetric resemblance of the overall transcriptome toward the D-genome diploid parent, as noted previously (Flagel et al. 2008; Rapp et al. 2009; Yoo and Wendel 2014). This trend was consistent across different mapping strategies (supplementary tables S16 to S18, Supplementary Material online).

When expression was compared between homoeologs, HEB was detected for 6.5% (***B*** ≠ 0) and 14.1% (***Bp*** ≠ 0) of genes in the F_1_ and AD_1_, respectively (Fig. 6d; supplementary table S18, Supplementary Material online), representing a greater than 2-fold increase in HEB in the allopolyploid. While no overall imbalance in HEB was detected in the F_1_, in the allopolyploid more homoeologous pairs exhibited D- (vs. A-) biases, regardless of mapping strategy. Allele-specific expression (ASE) analysis revealed 12.8% of genes exhibited parental expression divergence between A_2_ and D_5_, whose inferred regulation can be subdivided into the previously described categories (Hu and Wendel 2019): *cis* only (I—849 genes), *trans* only (II—62 genes), *cis* and *trans* enhancing (III—9 genes), and *cis* and *trans* compensating (IV—8 genes). Notably, these results ascribe an order of magnitude greater influence of *cis* variation in expression evolution between the diploid cottons (supplementary table S18, Supplementary Material online), suggesting that *cis* evolution has played a dominant role in generating expression variation between those species. In terms of the evolutionary impact of genome polyploidy, genome doubling (***Wr*** ≠ 0, 2.9%) has a much stronger effect than hybridization (***Hr*** ≠ 0, 0.6%), representing two distinct phases of allopolyploidization (***Pr*** ≠ 0, 4.7%). These results also suggested that the relative expression of At versus Dt homoeologs in F_1_ and AD_1_ was mainly determined by the parental state of A_2_ versus D_5_ (***Hr*** = 0, 99.4%; ***Pr*** = 0, 95.3%), also known as “parental legacy” (Buggs et al. 2014). Only a small portion of At versus Dt ratios were distinct from the parental states, a situation known as “regulatory novelty” by hybridization and allopolyploidization (***Hr*** ≠ 0 and ***Pr*** ≠ 0, as illustrated in Fig. 6c).

### Promoter Accessibility Regulates Duplicated Gene Expression Patterns

**Fig. 7.**
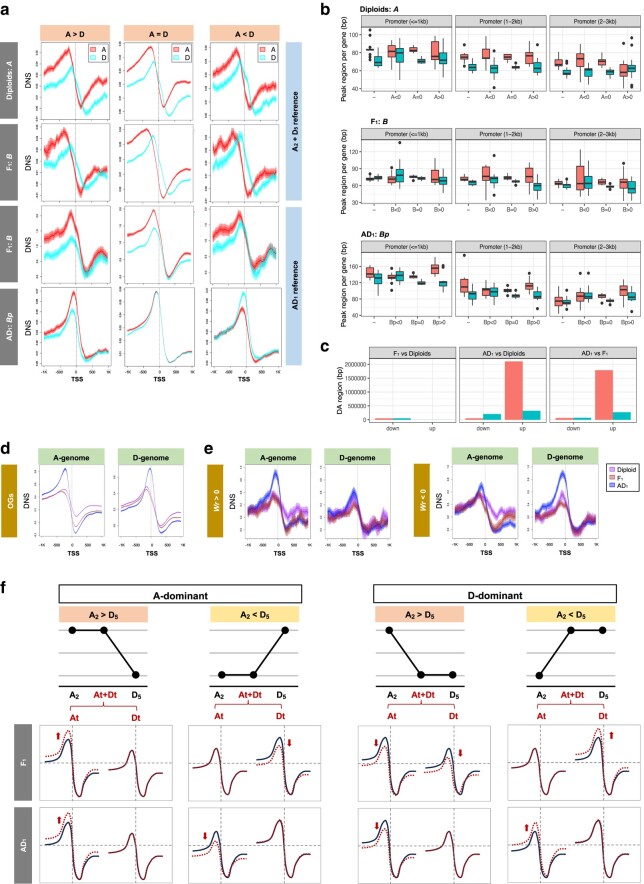
Promoter accessibility of duplicated genes in diploid and allopolyploid cottons. a) Aggregation plots of DNS signals were present in association with duplicated gene expression patterns of parental divergence (***A*** ≠ 0; top row), HEBs in F_1_ (***B*** ≠ 0; middle two rows) and in AD_1_ (***Bp*** ≠ 0; bottom row). The *x*-axis is centered on TSS ± 1 kb. The *y*-axis represents RPGC (reads per genomic content) normalized occupancy performed by deepTools (Ramírez et al. 2014). Each center line represents the aggregated mean occupancy, with ribbons representing the 95% confidence interval. Consistent patterns were also observed from analyzing the chromatin accessibility signals profiled by DNase-seq (Han et al. 2022) as shown in supplementary fig. S12, Supplementary Material online. b) Boxplot of promoter ACR sizes in association with duplicated expression patterns. Using the bottom row “AD_1_: ***Bp***” as an example, promoter ACRs were further classified to three promoter regions (<=1 kb, 1 to 2 kb, and 2 to 3 kb) for presentation; within each panel, the ACR sizes per gene were contrasted between At and Dt for different expression patterns (***Bp*** > 0 indicates higher At vs. Dt expression in AD_1_, ***Bp*** < 0 indicates higher Dt vs. At expression, and ***Bp*** = 0 indicates equal homoeolog expression; “-” refers to inconsistent results from different mapping strategies). c) Bar plot of DA region sizes in pairwise comparisons between diploids, F_1_, and AD_1_ in 1 kb promoters. Within each plot panel, the increase and decrease of accessibility were plotted for A- and D-genomes as color-coded in a). d) For 22,889 OGs, aggregation plots of DNS signals were presented based on A_2_ and D_5_ references. e) For OGs exhibiting genome doubling effects on expression (283 ***Wr*** > 0 and 396 ***Wr*** < 0), aggregation plots of DNS signals were presented based on A- and D-subgenomes of AD_1_ reference. f) Corresponding to four ELD patterns, the modes of promoter accessibility changes were depicted for At and Dt homoeologs corresponding to their total expression patterns.

To explore the links between chromatin architecture and expression evolution, we next examined the promoter accessibility profiles as measured by DNS signals in association with various duplicated gene expression patterns. For a total of 22,889 orthogroups (OGs, see above), the parental A_2_ and D_5_ accessibility profiles were generated by mapping diploid DNS-seq reads to their corresponding reference genomes, and direct comparison of these profiles revealed a systematic left-ward shift of peak and higher accessibility in A_2_ and D_5_ around TSSs, regardless of the direction of parental expression divergence (Fig. 7a, top row). Such a pattern is likely due to differences in gene annotation between the two diploid reference genomes, which thereby limits our ability to directly detect accessibility changes associated with parental expression divergence (***A*** ≠ 0). In contrast, the use of the allopolyploid reference genome revealed that promoter accessibility is positively correlated with homoeologous expression levels; that is, higher A- versus D-promoter accessibility was observed for the homoeologous gene pairs exhibiting A-biased HEB (***Bp*** ≠ 0), and higher D- versus A-promoter accessibility was observed for pairs exhibiting D-biases (Fig. 7a, bottom row). Additionally, the homoeolog that exhibited biased higher expression tended to display larger ACRs within 1 kb of the TSS (Fig. 7b). For HEB in F_1_ (***B*** ≠ 0), the homoeologous accessibility profile based on diploid reference genomes (A_2_ and D_5_ concatenated) exhibited the similar pattern as observed between diploids (Fig. 7a, the second row from top); interestingly, the use of the allopolyploid (AD_1_) reference eliminated the positional shift between A- and D-peaks, while the higher A- than D-promoter accessibility level remained, regardless of the direction of HEB (Fig. 7a, middle two rows). Although we cannot rule out artifacts introduced by either reference, the distinct patterns in diploid hybrid versus allopolyploid cotton indicate that hybridization alone does not alter the relationship between gene expression and promoter accessibility, but the allopolyploid evolution does. In addition to OGs, we also characterized promoter accessibility for genes that cannot be confidently assigned to OGs (referred to as nonOGs: A_2_—18850; D_5_—14329; At—13227; Dt—15895) and found distinct patterns between OG and nonOG genes (supplementary fig. S9, Supplementary Material online). In allopolyploid cotton, a higher A- than D-accessibility was shown for all genes and nonOGs, whereas comparable A- and D-accessibility levels were shown for OGs.

Given the strong evidence of “parental legacy” of hybridization with respect to both nucleosome organization (Fig. 2) and gene expression (Fig. 6), we hypothesized that in the hybrid, promoter accessibility and its regulatory consequences on gene expression would be primarily vertically inherited and thus mirror parental profiles. To test this hypothesis, we examined the relative A- versus D-genome chromatin accessibility profiles for categorized expression patterns, by normalizing the A-genome profiles (A_2_, F_1_:At, and AD_1_:At) and D-genome profiles (D_5_, F_1_:Dt, and AD_1_:Dt) against their corresponding diploid references by genomic content (supplementary figs. S10 and S11, Supplementary Material online). The results revealed a relatively slight decrease by hybridization and a much stronger increase by allopolyploidization in accessibility (F_1_ < A_2_/D_5_ ≪ AD_1_) for both the A- and D-genomes, as evident in aggregation plots (Fig. 7d) and DA tests (Fig. 7c). The DA results also indicated more accessibility increases in At versus Dt promoters, consistent with the previous marginal comparison of ACRs (Fig. 3c and d). In association with the impact of genome doubling on gene expression, the up-regulation of At/Dt homoeolog expression ratios (***Wr*** > 0) was attributed to a biased increase in At promoter accessibility, while the down-regulation of At/Dt homoeolog expression ratios (***Wr*** < 0) were attributed to a biased increase of Dt promoter accessibility (Fig. 7e). No apparent accessibility patterns were observed with the impact of hybridization (***Hr*** ≠ 0 in 142 OG; supplementary figs. S10 and S11A, Supplementary Material online), likely due to small changes. These results show that “parental legacy” can be seen with chromatin structural features, implicating *cis*-regulation as a heritable feature of promoters in different genotypic backgrounds.

With respect to nonadditive patterns accompanying hybridization and polyploidy, we investigated how promoter accessibility changes of At and Dt homoeologs were associated with ELD in F_1_ (supplementary fig. S11B, Supplementary Material online) and AD_1_ (supplementary fig. S11C, Supplementary Material online); this analysis is summarized in Fig. 7f. In the diploid hybrid, when higher parental expression was detected in A_2_ than D_5_, hybridization appeared to further increase the promoter accessibility of At to establish the A-dominant ELD, and decrease the promoter accessibility of both At and Dt to establish the D-dominant ELD. Conversely, when higher parental expression was detected in D_5_ compared to A_2_, the hybridization appeared to further increase the promoter accessibility of Dt to establish the D-dominant ELD. The same was true for A-dominant ELD. Therefore, the regulatory effect of chromatin accessibility changes primarily affects the homoeolog with higher parental expression in F_1_. Interestingly, in allopolyploid cotton, accessibility changes were primarily in At promoters, likely due to sequence evolution accompanying natural allopolyploidization (Fig. 7f). These results demonstrate the distinct regulatory evolution accompanying hybridization versus allopolyploidization.

### Histone Gene Expression Evolution in Association with Nucleosome Organization as Mediated by Chromatin Accessibility

Because histone proteins are essential for nucleosome assembly, we next focused on histone gene expression to ask whether their expression levels vary between (sub)genomes and across ploidy levels, and how this relates to the observed nucleosome spacing patterns. In *G. hirsutum*, we identified 149 histone coding genes, including variants of core histones (H2A—24 At and 23 Dt, H2B—13&13, H3—18&18, and H4—14&16) and linker histones (H1—5 At and 5 Dt), based on phylogenetic relationships and amino acid sequence similarities with 50 well-characterized histone genes in *Arabidopsis* (supplementary table S19, Supplementary Material online). Estimates of the average evolutionary divergence for each family revealed that H1 and H2A comprise more divergent variants than the other families (*overall mean amino acid distance*: H1—0.53, H2A—0.44, H2B—0.16, H3—0.08, and H4—0.02), consistent with previous findings in animals and plants (Probst et al. 2020).

**Fig. 8.**
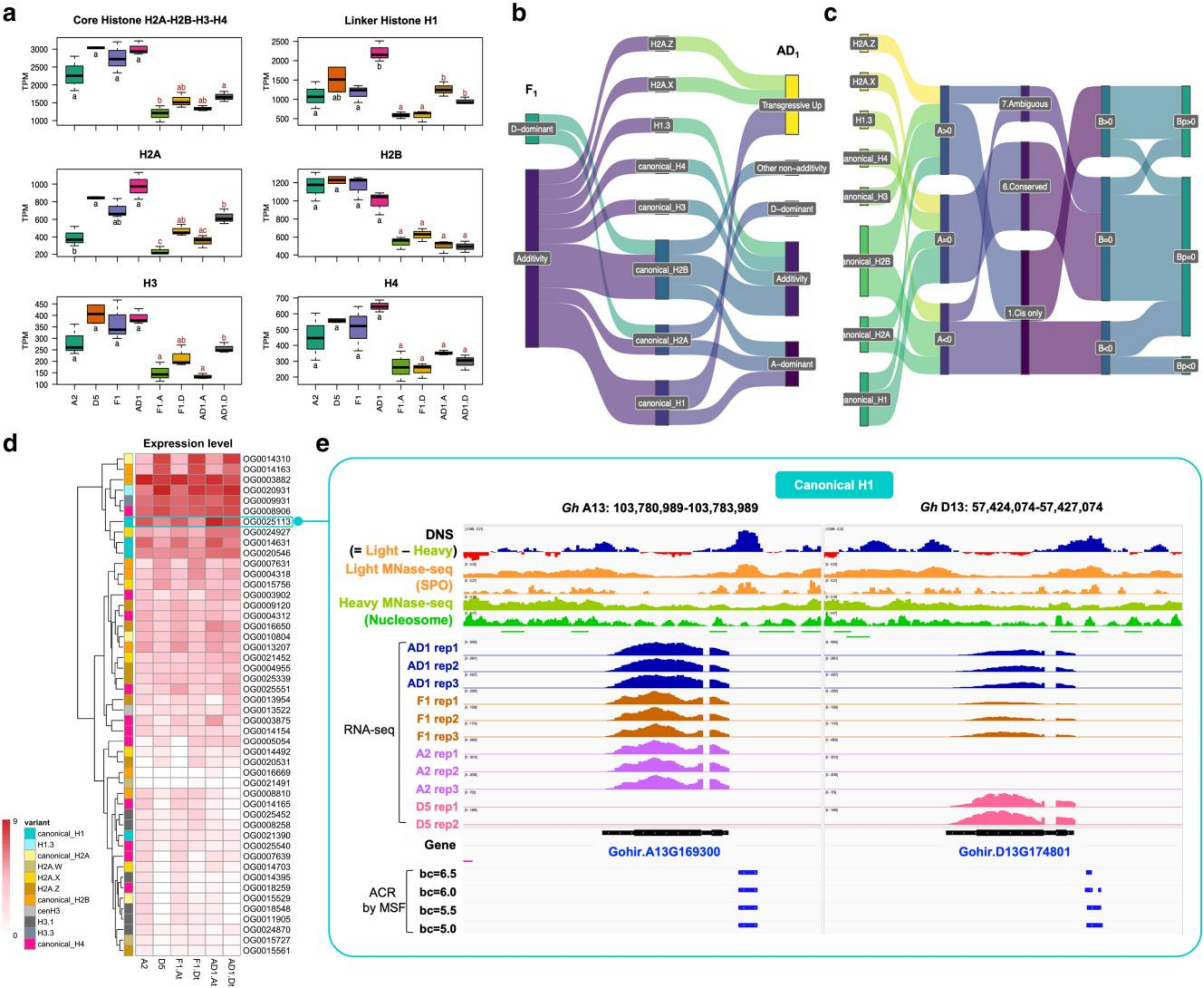
Analysis of histone gene expression. a) Boxplots present summed expression levels of histone gene family. Comparisons across diploid and allopolyploid cottons. Comparisons between (sub)genomes were performed using ANOVA with post hoc Tukey HSD test (*P* < 0.05). Groups with the same letter are not significantly different. b) The inheritance mode of parental histone expression was compared between F_1_ and AD_1_, as characterized by additive and nonadditive expression patterns (e.g. ELD and transgression). Categorization of different histone variants for OGs was depicted by the middle level of the Sankey diagram. c) Classifications of parental expression divergence (***A***), HEB in F_1_ and AD_1_ (***B*** and ***Bp***) were compared by Sankey diagram. d) Heatmap of histone gene expression profiles of 47 OGs. e) Genomic tracks illustrate DNS-MNase-seq and RNA-Seq profiles for a homoeologous pair of canonical H1 genes in *G. hirsutum.* Representatives of other histone variants were shown in supplementary fig. S14, Supplementary Material online.

To investigate the expression patterns of histone genes, we examined 47 OGs containing genes from the A_2_, D_5_, AD1:At, and AD1:Dt genomes (H1—4, H2A—17, H2B—7, H3—8, and H4—11) (supplementary table S20, Supplementary Material online). We found that the *total* expression of these genes was higher in the allopolyploid (mean sum transcripts per million [TPM] with standard deviation: 4988.4 ± 189.5) compared to the diploid genomes (3714.3 ± 301.1 in F_1_, 4338.3 ± 414.9 in D_5_, and 3207.2 ± 661.2 in A_2_), which agrees with the expectation that the allopolyploid genome contains more nucleosomes than do diploid genomes, such that histone transcription needs might be greater on a per cell basis. At the histone gene family level, notably, this increase in expression was particularly evident for linker histone H1 (Fig. 8a). At the histone variant level (supplementary fig. S13, Supplementary Material online), this pattern was observed for canonical H1, H2A.X, and H2A.Z variants, due to transgressive up-regulation of their gene members in allopolyploid cotton; interestingly, their counterparts in hybrid F_1_ tend to exhibit the D_5_-like expression.

Analyzing the expression difference between A- and D-(sub)genomes, we generally observed higher expression in the D-(sub)genome despite the overall lack of statistical significance (Fig. 8a; supplementary fig. S13, Supplementary Material online). Assuming histone expression levels correlate with the nucleosome number, it is intriguing that the smaller D_5_ diploid exhibited statistically equal, or even higher histone expression levels compared to the much larger A_2_ diploid (GS 0.8 Gb vs. 1.6 Gb). This finding is consistent with the nucleosome positioning result, i.e. smaller D_5_ diploid exhibiting higher NC, suggesting a speculation that histone gene expression may contribute to regulation of nucleosome spacing.

At the OG level (i.e. expression by gene), more nonadditive expressions were detected in the allopolyploid than hybrid (Fig. 8b). Directions of parental divergence (i.e. ***A*** > 0 and ***A*** < 0) and HEB (i.e. ***B*** > 0 and ***B*** < 0, or ***Bp*** > 0 and ***Bp*** < 0) were more or less balanced, which were often influenced by *cis*-only regulation, and no significant *trans*-regulatory divergence was detected (Fig. 8c). For instance, a larger and more prominent promoter ACR region was found associated with higher expression of the canonical H1 gene in the At subgenome compared to the Dt subgenome (Fig. 8d and e: OG0025113—*Gohir.A13G169300* vs. *Gohir.D13G174801*). More examples are shown in supplementary fig. S14, Supplementary Material online.

## Discussion

In this study, we employed the MNase-based DNS-seq technique to examine chromatin structural features in the context of allopolyploid cotton, *G. hirsutum*, to address two primary questions regarding the evolutionary impact of allopolyploidization: (i) how does genome merger and doubling accompanying allopolyploidy alter chromatin structure; and (ii) what evidence can be obtained that connects the regulatory aspects of chromatin structure to the evolution of duplicated gene expression?

### Dissecting *Cis* and *Trans* Determinants of Polyploid Chromatin Evolution

With respect to the first question, our data suggest stronger effects on the genome-wide chromatin landscape by allopolyploidy than by hybridization (***Pr*** ≫ ***Hr***), noting that the former entails both genome doubling and, in the case of *Gossypium*, 1 to 2 million years of natural evolution as the lineage diversified and spread across the many regions in the American tropics. Notably, a preponderance of chromatin alterations appears to have been driven by sequence evolution acting in *cis*. First, relative to the parental A_2_ and D_5_ diploids that model the allopolyploid progenitors, only slight changes of nucleosome organization (Fig. 2b and c) and chromatin accessibility (Fig. 7c) were detected in the F_1_ hybrid, with allele-specific patterns closely mirroring those of diploids. This lack of deviation from vertical transmission of preexisting chromatin patterns clearly indicates strong “parental legacy” (Buggs et al. 2014) by hybridization, as well as the *cis* nature of parental divergence on chromatin features, in accordance with the classic ASE model (Wittkopp et al. 2004).

Next, a multilevel synchronization effect was evident in the allopolyploid, which has assimilated various sequence-based and chromatin level features of both the A and D progenitor genomes, including nucleosome spacing (Fig. 2b and c), ACR classification (Table 1), genomic TE content and distribution (Fig. 5a and supplementary fig. S6, Supplementary Material online), accessibility round TSS (Fig. 7a), and the promoter *cis*-regulatory landscape (Fig. 4). These results are consistent with the previous study of genome-wide chromatin analysis in diploid and polyploid cottons using DNase-seq and further enrich the evidence of synchronization effects based on DHS accessibility and histone modification marks (Han et al. 2022). Han et al. (2022) also examined DHS distribution patterns in wild versus domesticated *G. hirsutum*, cultivated *G. barbadense*, and wild *G. darwinii*, revealing convergent DHS distributions between subgenomes in all of these allopolyploid cotton genotypes. However, despite the commonalities between At and Dt subgenomes, significant differences in marginal DHS distribution patterns were observed among species. For instance, 12.3% to 13.5% of DHS are genic in *G. hirsutum*, 9.0% to 9.7% in *G. barbadense*, and 31.5% to 32.3% in *G. darwinii*. These interspecific variations that remain to be further characterized underscore the importance of conducting comparative analyses between species and even incorporating multiple accessions from the same species. Recognizing the limitation of our study, which included only one accession each for allopolyploid and diploid genomes, future studies would benefit from including multiple accessions to enhance our understanding of chromatin evolution in cotton.

Notably, although the synchronization effect accompanying allopolyploidy resembles the *trans* effect in the synthetic hybrid, it cannot be simply interpreted according to the classic ASE model. As previously proposed (Hu and Wendel 2019), an extended *cis*–*trans* framework is required to delineate the *cis* and *trans* determinants of gene expression that arise from genome doubling following hybridization. That is, under the common *trans* environment experienced by both subgenomes in the allopolyploid, the partitioning of *cis*–*trans* regulation needs to be conceptually modeled into inter- and intrasubgenomic interactions, based on integrated analysis of genetic and epigenetic variations. While more sophisticated computational modeling and molecular tools are needed to fully elucidate these interactions, we demonstrated the use of computational prediction to pinpoint *cis* determination of nucleosome positioning (Fig. 2d and e), where reduced difference in nucleosome spacing by allopolyploidy can be predicted by DNA sequence per se. It has been recognized that nucleosome formation favors the periodic distribution of the dinucleotides GG, TA, TG, and TT at contact points between DNA and histones (every ∼10 bp) and sequences such as poly(dA:dT) that require high DNA bending energy tend to be avoided (Kaplan et al. 2009; Segal and Widom 2009). Therefore, nucleosome positions represent sequence-encoded functional features, which can therefore be selected during evolution (Barbier et al. 2021). We hypothesize that subgenomes in allopolyploids could be differentially selected (toward convergence) not only for their homoeologous gene content, but also for their ability to favor or impair nucleosome formation at genome-wide scale to facilitate chromatin package and/or at specific loci to impact accessibility to regulatory factors that mediate selectively favored gene expression. Future studies involving additional allopolyploid systems and tissue types will be instrumental in this hypothesis of nucleosome evolution.

In contrast to the *cis* determination of synchronization in terms of nucleosome spacing and promoter accessibility, the characteristics of nucleosome positions turned out to be strongly shaped by *trans* factors, as evidenced by disparity between experimental observations and DNA predictions (Fig. 2). That is, distances between consecutive nucleosomes were greater in A- than in D-(sub)genomes, whereas the opposite patterns were suggested by the computational prediction of nucleosome occupancy from DNA sequences alone. With a fixed length of ∼147 bp for canonical nucleosomes, NRL ranges from 154 bp in fission yeast (Lantermann et al. 2010) to 240 bp in echinoderm sperm (Athey et al. 1990), depending on species, tissue type, and experimental conditions. Studies on yeast, animal, and human have shown that NRL tends to be shorter in transcriptionally active genomes, such as embryonic stem cells and tumor cells compared to echinoderm sperm, or active gene regions compared to heterochromatic noncoding sequences (Barbier et al. 2021). Notably, telomeric chromatin stands as an exception to this rule, exhibiting an unusually short NRL and high sensitivity to MNase (Tommerup et al. 1994) due to its unique columnar conformation of nucleosome stacking (Soman et al. 2022).

In plants, MNase digestion analysis of cereal species has revealed a typical NRL of 175 to 185 bp, with shorter NRLs observed in telomeric nucleosomes compared to bulk nucleosomes (Vershinin and Heslop-Harrison 1998). Additionally, intriguing differences in MNase kinetics were observed between rye (7.8 Gb, 2*n* = 14) and wheat (160 Gb, 2*n* = 42), where the shorter NRL and faster MNase cleavage of the smaller rye genomes were proposed to be influenced by its prominent subtelomeric heterochromatin. Recent phasogram analyses using mononucleosomal MNase-seq have also been conducted in *Arabidopsis* (135 Mb; NRL of 185.1 bp in leaves and 182.2 bp in flowers), rice (430 Mb; 188 bp in leaves), and maize (2.4 Gb; 193.5 bp in shoots and 190.7 bp in endosperm) (Zhang et al. 2015; Chen et al. 2017), further supporting the trend of larger nucleosome spacing in larger genomes, as observed here for cotton. In both rice and arabidopsis, heterochromatic regions were found to have larger nucleosome spacing compared to euchromatic regions marked by various histone modifications (Zhang et al. 2015). Similarly, in maize, intergenic regions exhibited larger spacing than the genome-wide NRLs (Chen et al. 2017). Differential spacing of nucleosomes associated with distinct genomic regions has also been reported in the human genome (Valouev et al. 2011). Such variations of NRLs have been well recognized to direct the folding of nucleosome arrays into chromatin fibers (Fransz and de Jong 2011; Brouwer et al. 2021): Evidently, longer linker DNA (197 bp vs. 167 bp) together with the binding of linker histones (H1, H5) are required for a further compaction and stabilization of the 30 nm chromatin fiber, as associated with a repressed chromatin state. Indeed, we identified significantly higher expression levels of the linker histone H1 corresponding to larger NRLs in A- versus D-(sub)genomes, as well as the allopolyploid versus diploids.

Hence, it is plausible that plant genomes with larger sizes and higher ploidy levels have undergone adaptations resulting in larger nucleosome spacing, potentially facilitating specific high-order chromatin organizations. Additional studies are necessary to test this hypothesis. Apart from the *cis*-regulatory role of DNA sequences in nucleosome organization, there are several *trans* factors that contribute to this process, including histone variants, posttranslational histone modifications, chromatin remodeling enzymes, and various architectural proteins (Arya et al. 2010). To fully understand the complex interplay between *cis* and *trans* elements in shaping nucleosome organization in polyploid plant genomes, it will be crucial to investigate the sequence and functional evolution of these factors accompanying allopolyploidization.

### Regulatory Relationships among Chromatin Evolution and Duplicated Gene Expression

To address our second main question, above, regarding regulatory control of gene expression evolution accompanying allopolyploidization, we investigated the role of promoter accessibility in shaping various well-recognized phenomena of duplicated gene expression, including asymmetric resemblance of parental diploids, HEB, nonadditive inheritance modes, and genome impact of hybridization (***Hr***) and allopolyploidization (***Pr***). Central to this investigation was also the extended *cis*–*trans* analytic framework (Hu and Wendel 2019), which enabled us to first systematically characterize these duplicated gene expression patterns (Fig. 6), and next disentangle the regulatory effects of chromatin accessibility (Fig. 8). By exploring interconnecting patterns among chromatin traits and duplicate gene expression patterns, our study provides several perspectives into the regulatory underpinnings that govern allopolyploid gene expression dynamics.

#### Regulatory Relationships to Homoeolog Expression Bias

The positive correlation between promoter accessibility and gene expression levels reaffirmed the anticipated connection between HEB direction and accessibility in the allopolyploid; that is, the homoeolog exhibiting higher expression level exhibits greater promoter accessibility than its alternative duplicated copy. However, this regulatory connection was not observed in the synthetic diploid hybrid, which exhibited a systematic asymmetry of higher A- than D-promoter accessibility, irrespective of HEB direction (Fig. 7a). This observation suggests that hybridization by itself generates “mismatches” between gene expression and chromatin accessibility, raising intriguing questions about the temporal scale and mechanisms in establishing their regulatory relationships during allopolyploid formation and evolution. One other implication is that HEB is determined by chromatin features or transcriptional factors other than or in addition to promoter accessibility.

#### The Temporal Scale of Regulatory Evolution

Assessment of ***Hr*** and ***Pr*** revealed contrasting effects of immediate hybridization and evolution of the cognate allopolyploid lineage. Hybridization is shown to be characterized primarily by parental legacy, manifested as mostly “vertical inheritance” of expression levels with minor changes in both accessibility and expression. In contrast, allopolyploidization exerts a pronounced impact, leading to substantial accessibility increases attributed to genome doubling and subsequent sequence evolution. Furthermore, the homoeolog-specific accessibility increase was notably associated with shifts in homoeolog expression ratios (e.g. ***Wr*** > 0 or ***Wr*** < 0 in Fig. 7e), underlining the regulatory influence of chromatin dynamics. Our promoter analysis highlights the potential role of sequence evolution in reducing TE contents and introducing *cis*-regulatory footprints into gene promoter regions, thereby impacting chromatin accessibility and gene expression evolution. Relationships between these dynamics and the multiple cascading spatial and stoichiometric effects of genome doubling (Bottani et al. 2018; Doyle and Coate 2019) comprise a promising direction of future research.

#### Nonadditive Inheritance Modes

Although allopolyploidization led to accessibility increases, we did not detect a significant amount of transgressive up-regulation of gene expression relative to parental diploids, as might have been expected. This observation implicates additional regulatory influences and perhaps stoichiometric controls on gene expression, the identification of which also comprises an interesting research direction. The phenomenon of ELD, another well-known yet mechanistically mysterious nonadditive expression pattern, perhaps exemplifies the complexities of the interplay between chromatin accessibility and gene expression. Our study demonstrates that changes in chromatin accessibility predominantly impact the homoeolog with higher parental expression in the F_1_ generation; in contrast, allopolyploidy is characterized by a distinctive pattern in which accessibility changes predominantly occur in At promoters, a shift likely driven by various biophysical and biochemical factors associated with ploidy stoichiometry, as well as sequence evolution linked to natural allopolyploidization (Fig. 7f). Yoo et al. (2013) previously investigated homoeolog expression levels relative to ELD patterns and also showed that ELD reflects the up- or down-regulation of alternative homoeologs more frequently, compared to the up- or down-regulation of both homoeologs. The interrelationships among these dynamics remain to be elucidated.

### Concluding Remarks

Here, we show that promoter accessibility and nucleosome arrangement represent key components of the evolution of duplicate gene expression. It is important to acknowledge, though, that the realm of “chromatin structure” encompasses multiple molecular biological, quantitative, and spatial dimensions, with numerous mechanisms yet to be integrated into the needed synthesis. For instance, Han et al. (2022) examined the relationships between DHS accessibility and the various histone marks, demonstrating the coordinated dynamics among histone modifications, TEs, and DHS landscape under polyploidization. Additionally, the interplay between DNA methylation and chromatin accessibility remains to be further elucidated in response to hybridization and polyploidization. Between the parental diploids, the D-genome *G. raimondii* contains more TEs near genes than does the A-genome *G. arboreum*, and hence *G. raimondii* orthologs were generally more methylated (Song et al. 2017). Upon hybridization, CG and CHG methylation levels were conserved whereas CHH methylation levels were decreased in the synthetic F_1_, and the majority of these changes were conserved during the subsequent polyploid evolution. In the allopolyploid cotton, however, more CG methylation and lower euchromatic H3K4me4 levels (Zheng et al. 2016) were found in the At than Dt homoeologs, in association with more D-biased HEB. While our work also detected a significant imbalance of D-bias in AD_1_ (Fig. 6d), the globally higher promoter accessibility in the A- than D-genome remains enigmatic.

The orchestration of 3D chromatin organization is another crucial facet of chromatin evolution. Alterations in spatial subgenome distribution into different genome territories and long-range interactions within and between subgenomes intricately link to homoeologous gene expression (Pei et al. 2021). In cotton, allopolyploidization led to chromatin compartment switching and topologically associated domain (TAD) reorganization, both influencing gene expression dynamics (Wang et al. 2018). By leveraging Hi-C and DNase-seq data to uncover chromatin interactions and enhancer–promoter relationships, a long-range transcriptional regulation mechanism was proposed underpinning subgenome expression coordination and partitioning.

More recently, an innovative OCEAN-C approach was applied to map genome-wide open chromatin interactions for hexaploid wheat and its tetraploid and diploid relatives (Yuan et al. 2022). By integrating OCEAN-C, ChIP-seq, ATAC-seq, and RNA-seq data, the regulatory layers of structural variations, epigenetic marks, and chromatin accessibility were jointly investigated, collectively helping to reveal the role of open chromatin interactions in shaping gene expression variation during allopolyploid evolution.

In summary, our study details changes in chromatin features genome-wide, offering insights into how allopolyploidy affects nucleosome occupancy, chromatin accessibility, and the regulatory underpinnings of expression evolution of duplicated genes. Given the broader complexity of chromatin dynamics, exploring the synergies among histone modifications, DNA methylation, enhancer–promoter interactions, and 3D chromatin organization will continue to further our understanding of the intricate web of regulatory mechanisms in shaping gene expression evolution, and ultimately phenotypic evolution, in cotton and other allopolyploid systems.

## Supplementary Material

msae095_Supplementary_Data
