# Supplementary material for: Evolutionary Dynamics of Chromatin Structure and Duplicate Gene Expression in Diploid and Allopolyploid Cotton
Source: Mol Biol Evol. 2024 May 17;41(5):msae095. doi: 10.1093/molbev/msae095 (PMC11140268; doi:10.1093/molbev/msae095)
Supplement: msae095_Supplementary_Data [file msae095_supplementary_data.zip › 2.Supplementary text.Revision.pdf]

Supplementary Information

Evolutionary dynamics of chromatin structure and duplicate gene expression in diploid and allopolyploid cotton

Guanjing Hu[1,2*], Corrinne E. Grover[3], Daniel L. Vera[4], Pei-Yau Lung[5], Senthil B. Girimurugan[6], Emma R. Miller[3], Justin L. Conover[3,11,12], Shujun Ou[7], Xianpeng Xiong[2], De Zhu[2], Dongming Li[8,2], Joseph P. Gallagher[9], Joshua A Udall[10], Xin Sui[5], Jinfeng Zhang[5], Hank W. Bass[4*], Jonathan F. Wendel[3*]

[1] State Key Laboratory of Cotton Bio-breeding and Integrated, Institute of Cotton Research, Chinese Academy of Agricultural Sciences, Anyang, China 455000

[2] Shenzhen Branch, Guangdong Laboratory of Lingnan Modern Agriculture, Key Laboratory of Synthetic Biology, Ministry of Agriculture and Rural Affairs, Agricultural Genomics Institute at Shenzhen, Chinese Academy of Agricultural Sciences, Shenzhen, China 518120

[3] Department of Ecology, Evolution and Organismal Biology, Iowa State University, Ames, Iowa, USA 50011

[4] Department of Biological Science, Florida State University, Tallahassee, Florida, USA 32306

[5] Department of Statistics, Florida State University, Tallahassee, Florida, USA 32306

[6] Dept of Mathematics, Florida Gulf Coast University, Florida, USA 33965

[7] Department of Molecular Genetics, Ohio State University, Columbus, Ohio, USA 43210

[8] Zhengzhou Research Base, State Key Laboratory of Cotton Biology, School of Agricultural Sciences, Zhengzhou University, Zhengzhou, China 450000

[9] Forage Seed and Cereal Research Unit, USDA/Agricultural Research Service, Corvallis, Oregon, USA 97331

[10] Crop Germplasm Research Unit, USDA/Agricultural Research Service, College Station, Texas, USA 77845

[11] Department of Ecology & Evolutionary Biology, University of Arizona, Tucson, AZ, USA 85721

[12] Department of Molecular & Cellular Biology, University of Arizona, Tucson, AZ, USA 85721

* Correspondence: Jonathan F. Wendel (jfw@iastate.edu), Hank W. Bass (bass@bio.fsu.edu), and Guanjing Hu (huguanjing@caas.cn)

Abbreviation box

ACR, accessible chromatin region

ASE, allele-specific expression

ATAC-seq, assay for transposase accessible chromatin sequencing

BC, biological cutoff

DA, differential accessibility

DNase-seq, DNase I hypersensitive site sequencing

DHS, DNase I-hypersensitive site

DNS, differential nuclease sensitivity

DNS-seq, differential micrococcal nuclease digested nucleosomal DNA sequencing

ELD, expression level dominance

GS, genome size

HEB, homoeolog expression bias

MH, MNase hypersensitive

MNase-seq, micrococcal nuclease sequencing

MOA, MNase-defined cistrome-occupancy analysis

MSF, MNase sensitive footprints

NC, nucleosome coverage

NFL, nucleosome repeat length

NFR, nucleosome free region

OG, ortho-homoeolog group

RPM, reads per million

SPO, subnucleosomal particle occupancy

TE, transposable element

TSS, transcription start site

TTS, transcription termination site

Supplementary Text 1: Optimization of iSeg setting

To optimize iSeg performance, a range of biological cutoff (BC) stringencies from 4.0 to 7.0 (low to high) were applied to the DNS data. The positive DNS peaks identified represent MNase hypersensitive (HS) regions that are depleted of nucleosomes or relatively more accessible to regulatory transcription factors. Across diploid genomes (A_2 and D_5) and subgenomes (At and Dt) in F_1 and AD_1 genomes, the same stringency led to equal percentages of total genome sequence to be identified as ACRs: 1.85% at BC=4.0, 1.38% at BC=4.5, 1.06% at BC=5.0, 0.82% at BC=5.5, 0.65% at BC=6.0, 0.51% at BC=6.5, and 0.40% at BC=7.0. To understand how the choice of BC stringency affects ACR characterization, we inspected the profiles of ACR distribution across different stringency levels. Based on proximity to their nearest annotated genes, ACRs were categorized as genic (gACRs; overlapping a gene), proximal (pACRs; within 2 Kb of a gene) or distal (dACRs; >2 Kb from a gene). As the BC stringency increases, the ACR peak number and total length decrease (**Supplementary Text 1 Figure 1-2**), whereas the relative proportion of three ACR categories remains unaffected by stringency (**Supplementary Text 1 Figure 3-4**). Thus, we concluded that ACR identification and categorization is robust regardless of the choice of BC stringency.

We next assessed whether BC stringency affects direct ACR comparisons across genomes between diploids and their respective subgenomes in F_1. Along with the increased BC stringencies, the peak overlaps between diploid and F_1 decreased from 15.4% to 6.9%, suggesting that the number of overlaps decreased with the increased stringency level of ACR detection (**Supplementary Text 1 Figure 5**).

Given the amount of ACRs mapped by ATAC-seq and DNase-seq in cotton (0.45%-0.80% of genome; see main text and **Supplementary Text 2: ATAC-seq analysis in *G. raimondii***), we chose BC=6.0 as the stringency parameter. For sub-nucleosomal particle occupancy (SPO) scores, the following BC stringencies were used to identify 0.83% of A_2 genome at BC=4, 0.68% of D_5 genome at BC=5, 0.67% of AD_1 genome at BC=4, and 0.77% of $A_2 \times D_5$ genome at BC=4.5.

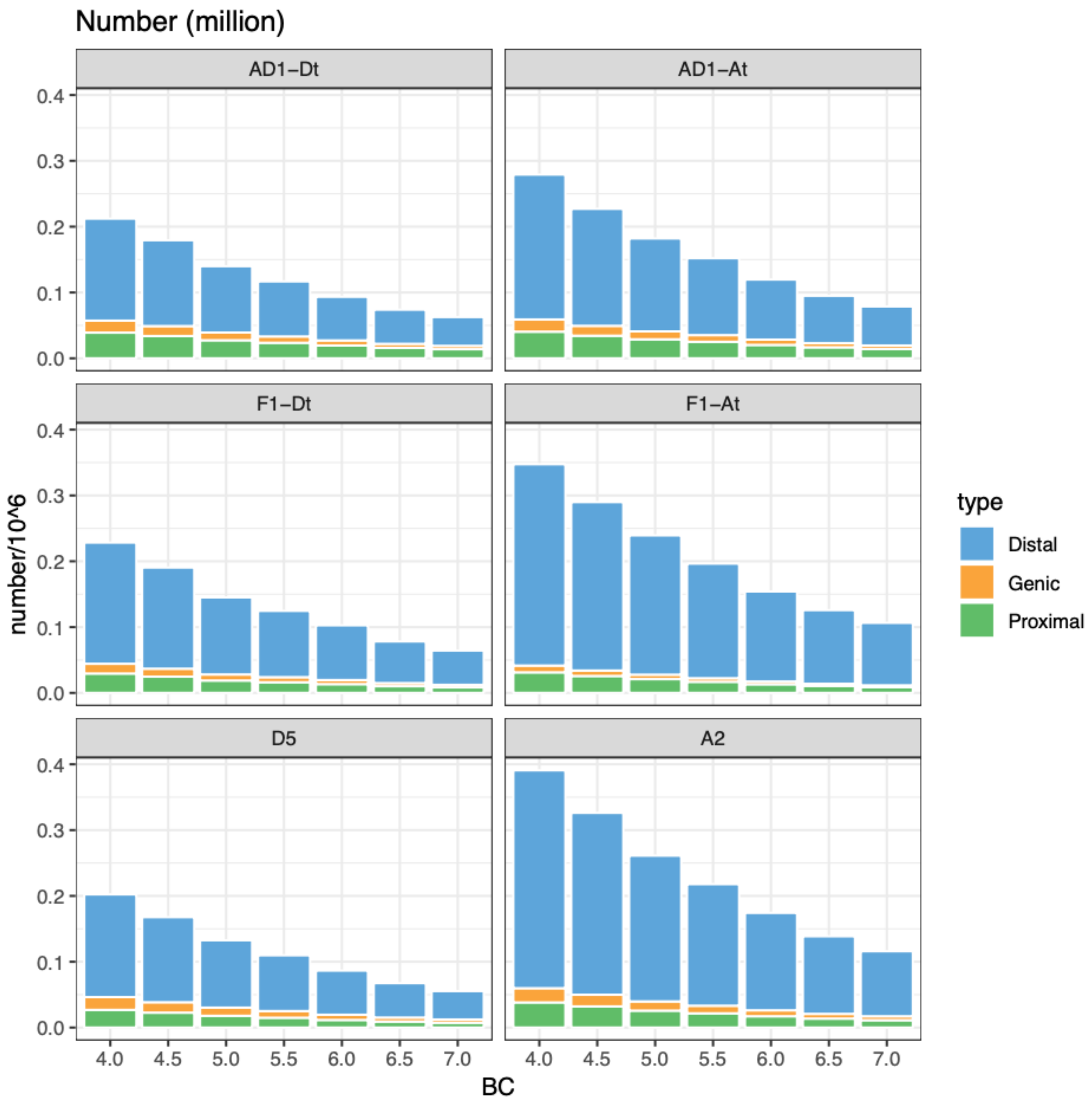

Supplementary Text 1 Figure 1. Peak number identified per genome under different iSeg stringencies.

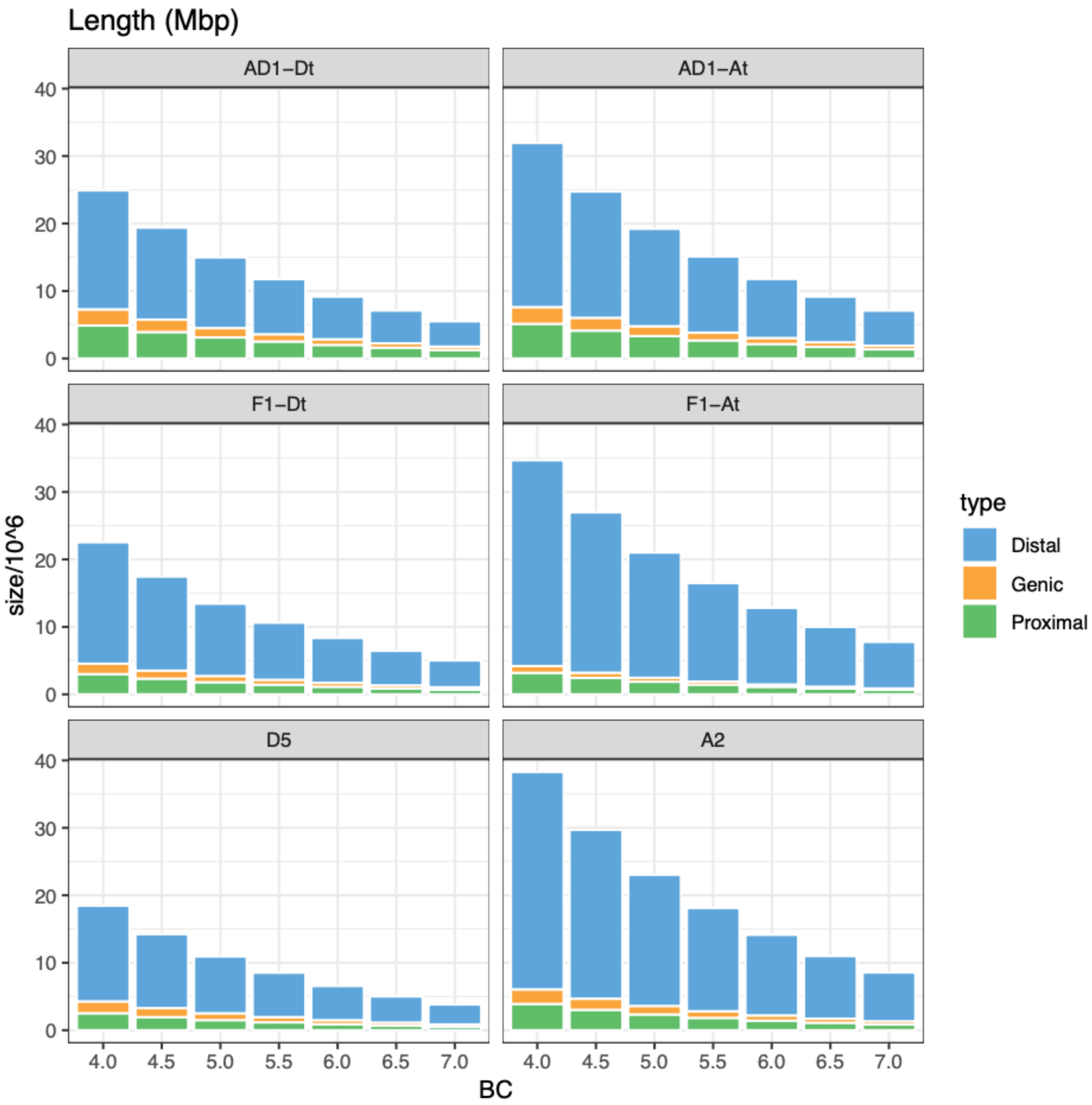

Supplementary Text 1 Figure 2. Peak length identified per genome under different iSeg stringencies.

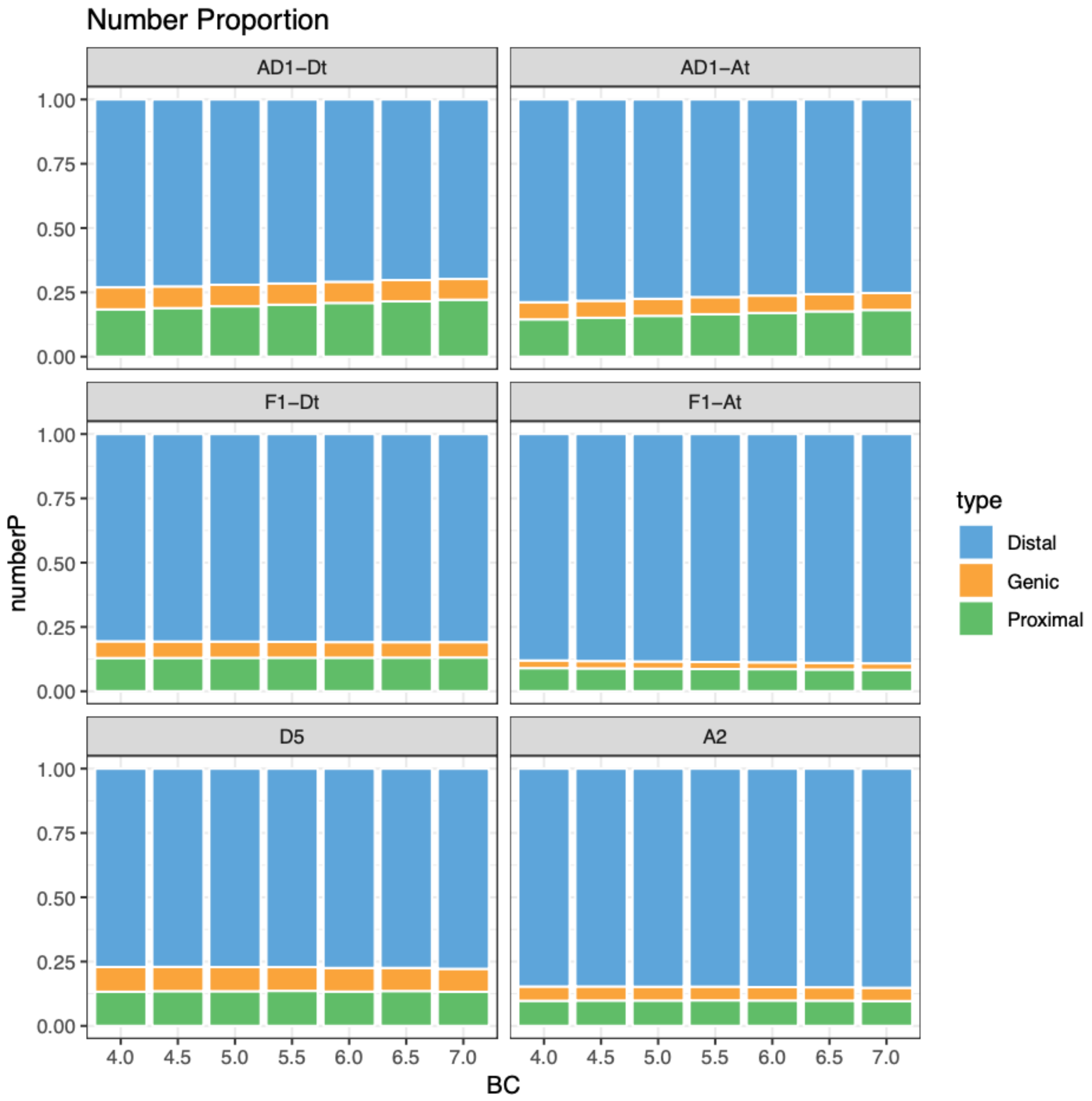

Supplementary Text 1 Figure 3. Relative proportion of peak numbers identified per genome under different iSeg stringencies.

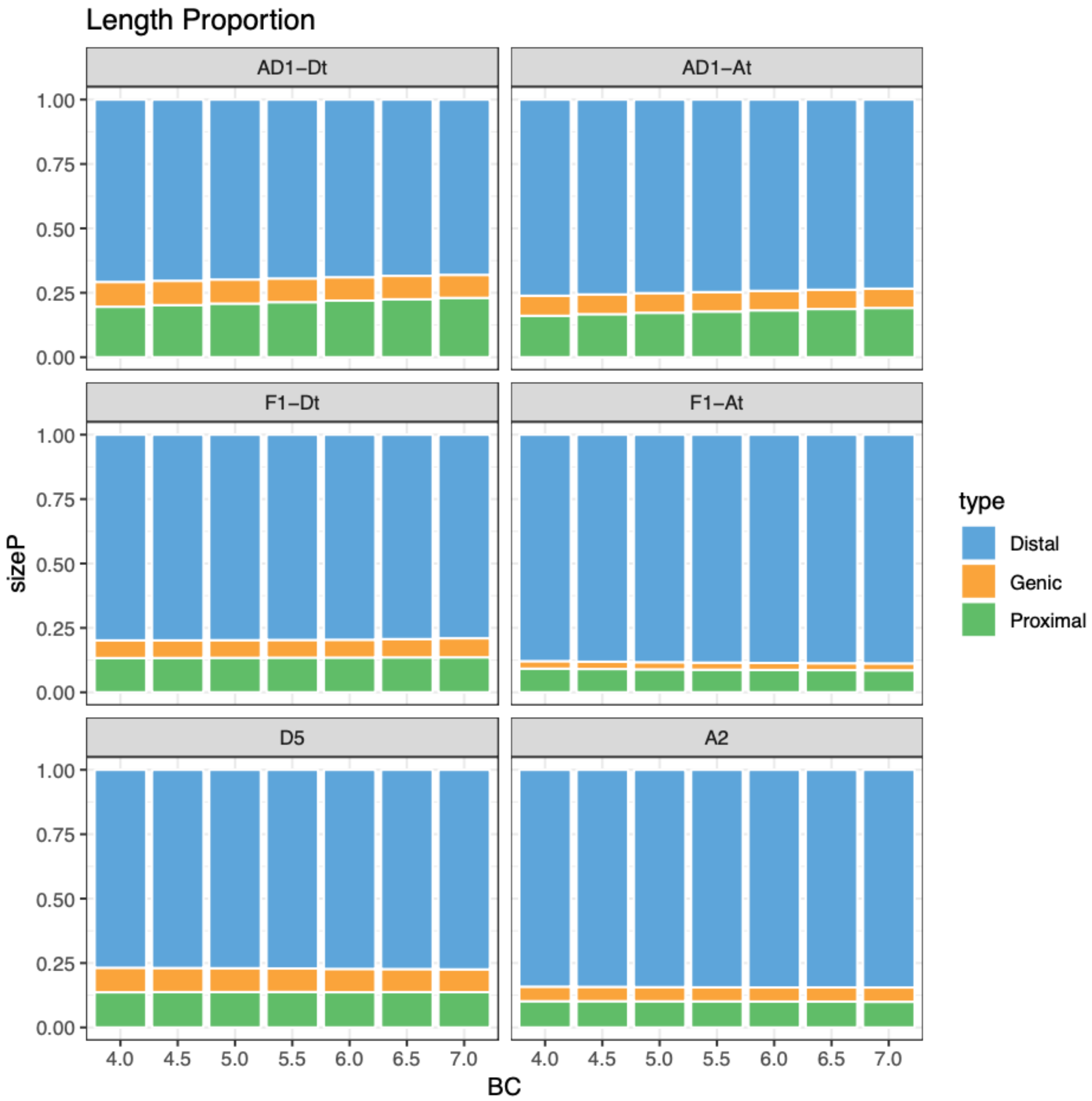

Supplementary Text 1 Figure 4. Relative proportion of peak length identified per genome under different iSeg stringencies.

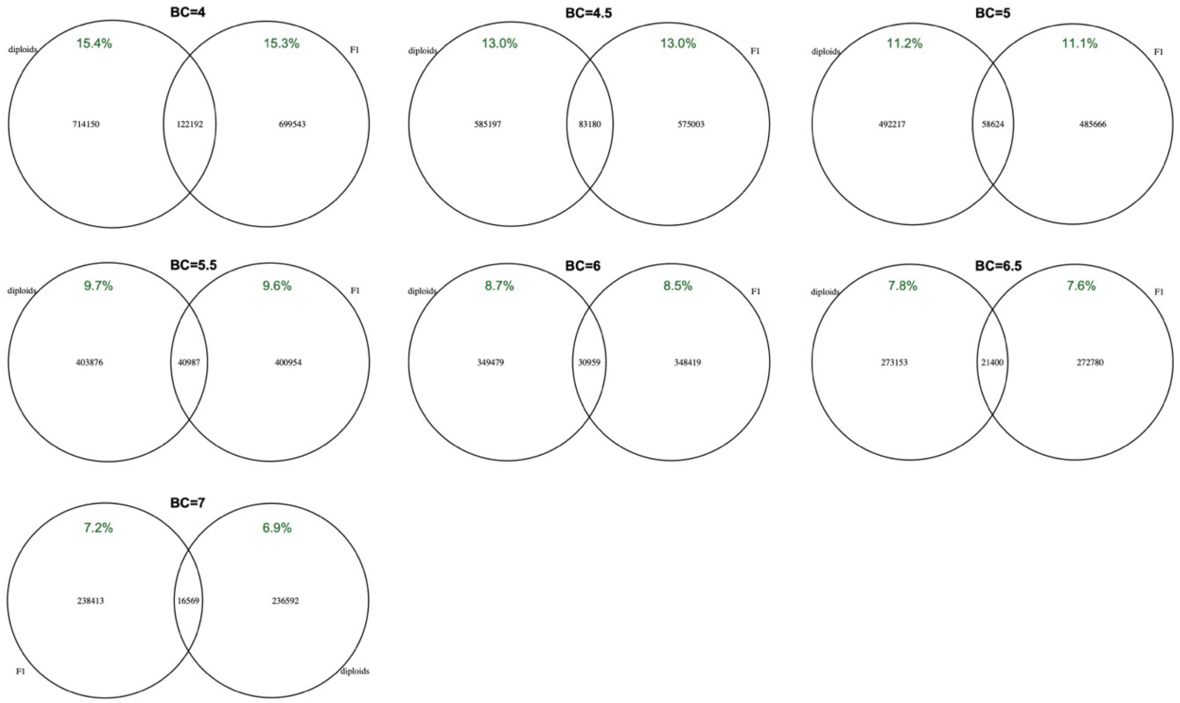

Supplementary Text 1 Figure 5. Peak comparisons between diploids and F_1 under different iSeg stringencies. To enable direct peak comparisons, regions independently detected from A_2 and D_5 genomes were combined as the diploid set. Venn diagrams show the overlap of regions by number between diploid and F_1, and their percentage of overlapped regions were shown in green.

Supplementary Text 2: ATAC-seq analysis in *G. raimondii*

We performed ATAC-seq to map accessible chromatin regions (ACRs) in young leaves. Two replicated ATAC-seq libraries were sequenced to 21.1 and 16.2 million reads per sample. The strand cross-correlation statistics supported the high quality of the ATAC-seq data, and the correlation of mapping read coverages (Pearson's $r = 1.00$ and Spearman's $r = 0.72$) suggested a high level of reproducibility between replicates (Table S4. Summary of ATAC-seq and DNase-seq data). Peak calling methods of HOMER, MACS2, and Genrich were applied for comparison, which identified 17,102 (6.0 Mb), 16,195 (5.5 Mb), 11,687 (4.6 Mb) ACRs by combining replicates, respectively (Table S5. ACR identification). Between these ACR lists, the overlaps of 41.2% to 79.5% indicated a relatively high consistency in ATAC-seq peak calling. The enrichment of ACRs around gene transcription start sites (**Supplementary Text 1 Figure 1**) suggested that these regions were potentially important for *cis*-regulatory control. Based on proximity to their nearest annotated genes, ACRs were categorized as genic (gACRs; overlapping a gene), proximal (pACRs; within 2 Kb of a gene) or distal (dACRs; >2 Kb from a gene). These three categories each represented about one third of the total number of ACRs, where Genrich detected more dACRs than did HOMER and MACS2 (41.9%, 31.3% and 35.2%, respectively; **Supplementary Text 1 Figure 2-4**, pie charts). The majority dACRs were located over 3 Kb from the nearest gene (**Supplementary Text 1 Figure 2-4**, density plots), and this finding of abundant dACRs implicated potentially long-range *cis*-regulatory elements and is consistent with the previous ATAC-seq studies in plants (Lu et al. 2019; Ricci et al. 2019). The three ACR categories exhibited similar width distribution and were 10% more GC-rich than the control regions that were randomly selected by maintaining the same width distribution (**Supplementary Text 1 Figure 2-4**, bar plot and boxplot). Because high GC content is associated with several distinct features that can affect the *cis*-regulatory potential of a sequence (Landolin et al. 2010; Wang et al. 2012), these results support the putative regulatory functions of ACRs. In addition, ACRs are generally depleted from TEs (negative enrichment scores from permutation tests).

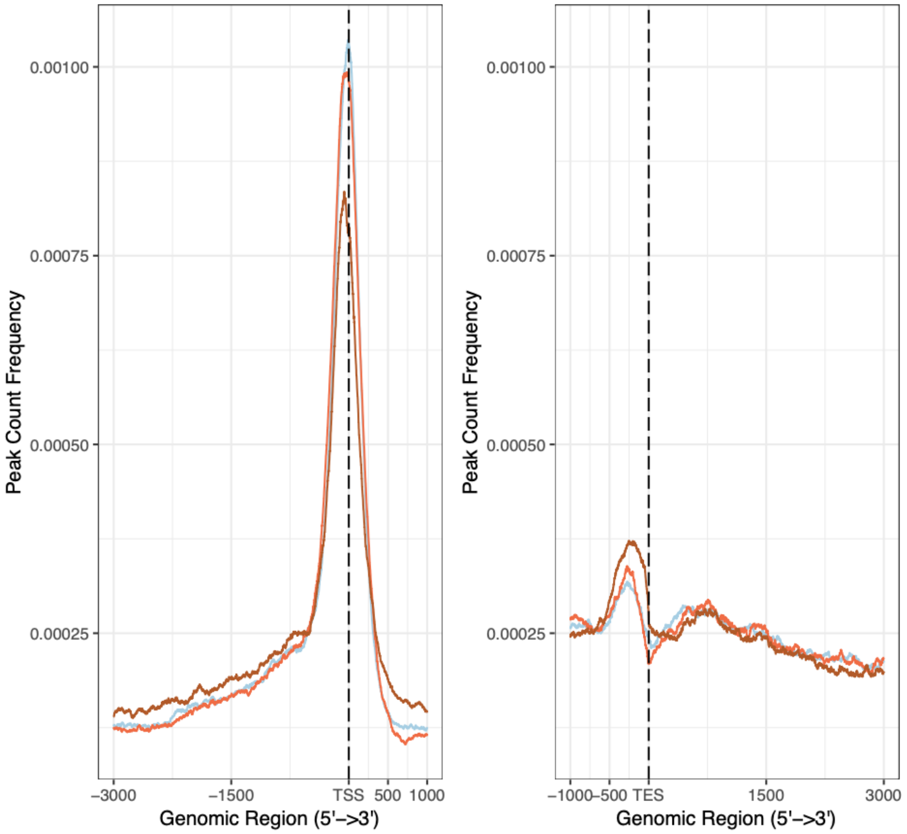

Supplementary Text 2 Figure 1. Peak count frequency of ACRs around gene transcription start sites (TSS) and transcription end sites (TES). Red, MACS2; blue, HOMER; brown - Genrich.

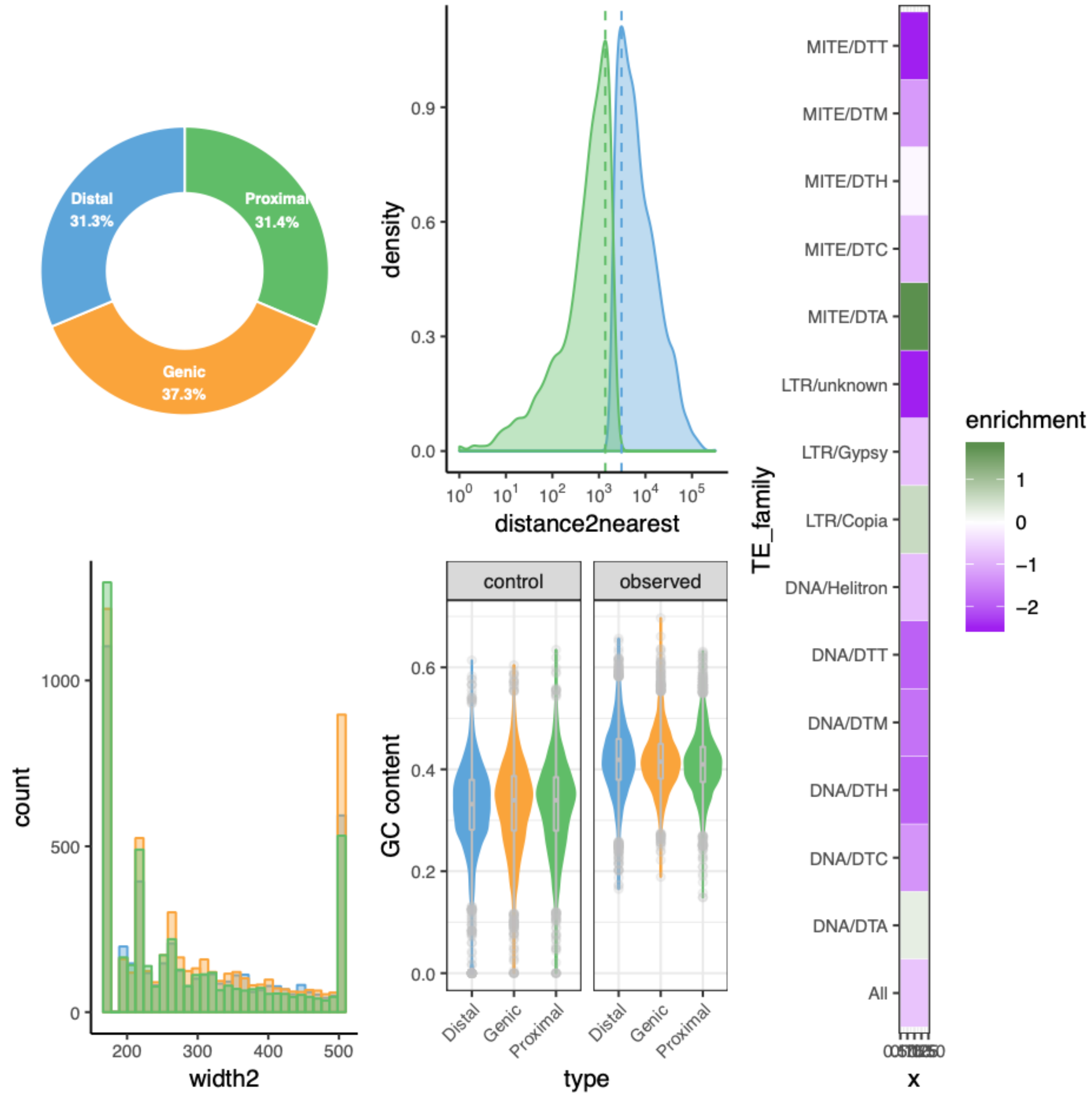

Supplementary Text 2 Figure 2. ATAC-seq ACRs called by HOMER. Top left pie chart presents the percentage of categorized ACR numbers. Top middle density plot presents the distance of proximal and distal ACRs to the nearest annotated genes. Bottom left bar plot present width distribution of ACRs. Bottom middle boxplot presents ACR GC contents as observed and simulated as control. The heatmap bar on the right presents the over- and under-representation scores of a certain transposable element (TE) family containing ACRs.

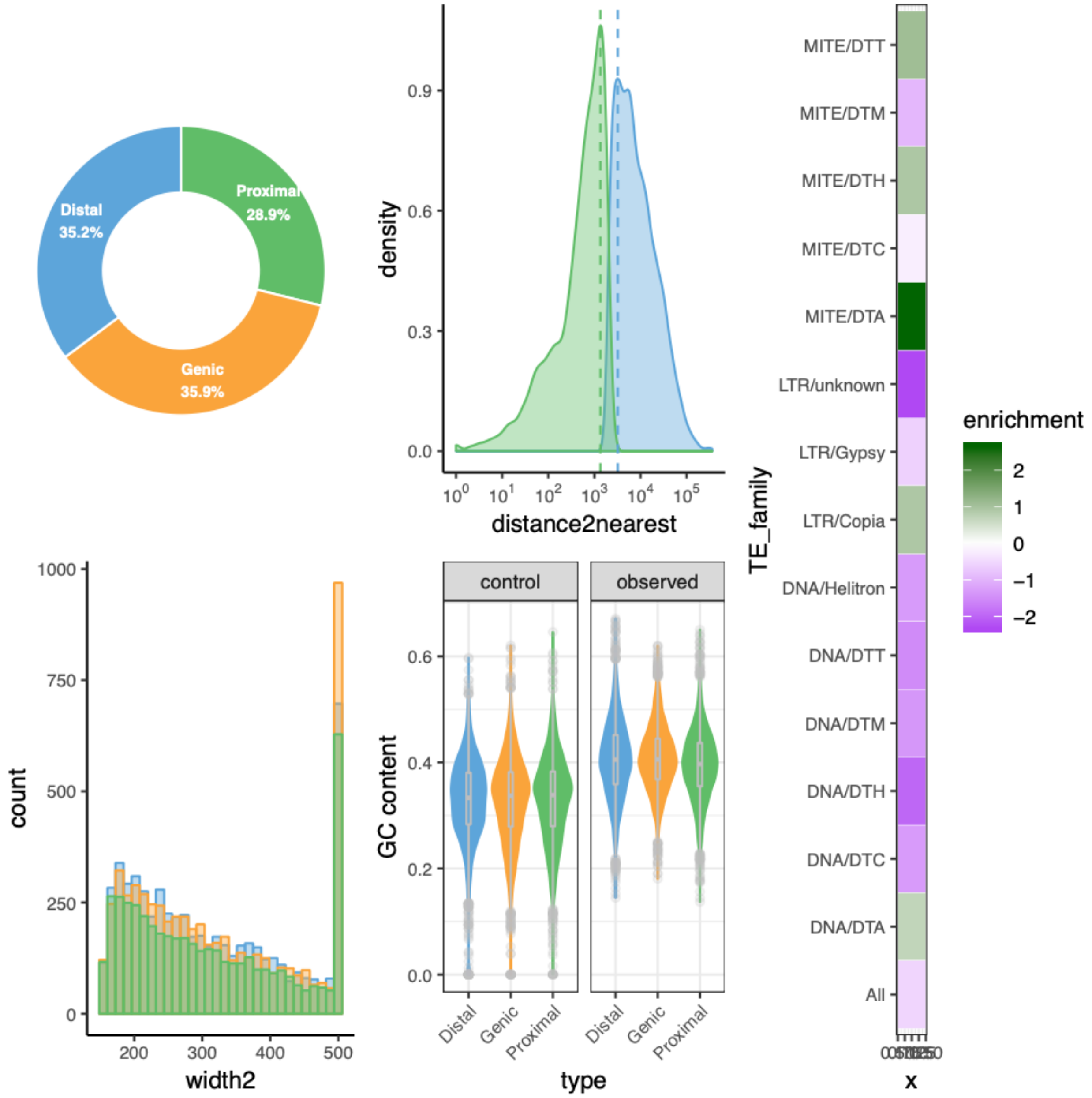

Supplementary Text 2 Figure 3. ATAC-seq ACRs called by MACS2. Top left pie chart presents the percentage of categorized ACR numbers. Top middle density plot presents the distance of proximal and distal ACRs to the nearest annotated genes. Bottom left bar plot present width distribution of ACRs. Bottom middle boxplot presents ACR GC contents as observed and simulated as control. The heatmap bar on the right presents the over- and under-representation scores of a certain transposable element (TE) family containing ACRs.

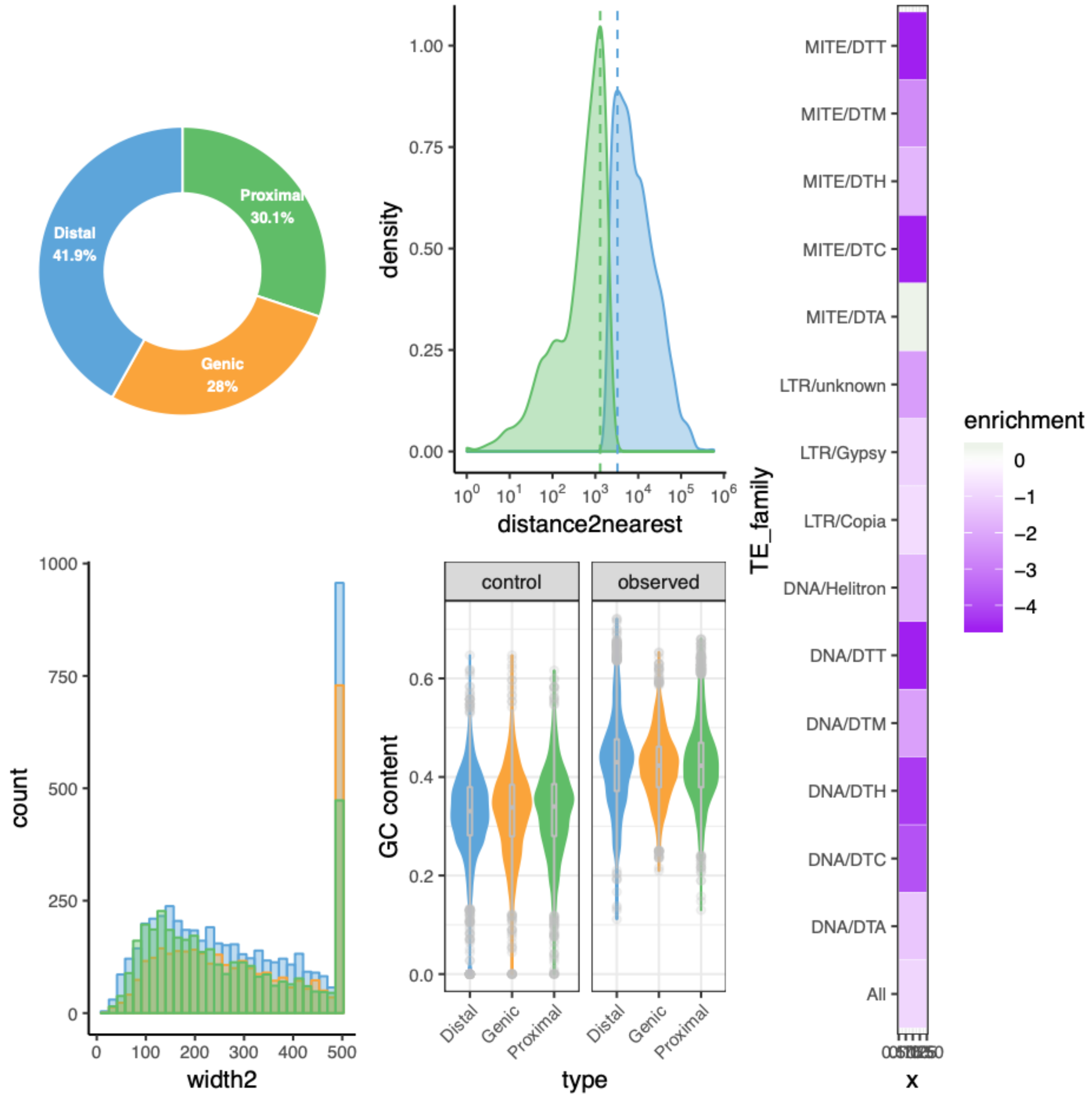

Supplementary Text 2 Figure 4. ATAC-seq ACRs called by Genrich. Top left pie chart presents the percentage of categorized ACR numbers. Top middle density plot presents the distance of proximal and distal ACRs to the nearest annotated genes. Bottom left bar plot present width distribution of ACRs. Bottom middle boxplot presents ACR GC contents as observed and simulated as control. The heatmap bar on the right presents the over- and under-representation scores of a certain transposable element (TE) family containing ACRs.

Supplementary Text 3: A hypersensitive region in *G. raimondii* chromosome 1

Chromatin accessibility assays, including differential MNase-seq, ATAC-seq, and DNase-seq analyses revealed a hypersensitive region in *G. raimondii* chromosome 1 (Ch01: 23,162,848 - 23,811,737; **Supplementary Text 3 Figure 1**). Derived from the high DNS scores (DNS=light-heavy), an unexpected high number of ACRs (1174 peaks of 417,072 bp) were identified from this 649 Kbp region. Previously, this region was annotated as a putative nuclear mitochondrial DNA sequence block, including many genes closely resembling mitochondrial homologues (Gorai.001G159900 - Gorai.001G168200) (Paterson et al. 2012). If this putative insertion of mitochondrial DNA was relatively recent, it may lack proper GC content or other physicochemical properties to array nucleosomes, thereby becoming highly sensitive to nuclease digestion. To test this hypothesis, we applied a sequence-based computational model to predict likelihood for nucleosome formation. Because the predicted nucleosome coverage of this region was even higher than the overall coverage in chromosome 1 (NC=78.2% vs 60.7%), we concluded that the observed hypersensitivity was unlikely to be intrinsically determined by DNA sequence. However, empirically, much lower nucleosome occupancies were observed in this region as revealed by heavy MNase digestion (NC=69.3% vs 91.2%; **Supplementary Text 3 Figure 1:** second track from top). This abnormal nucleosome organization may be controlled by *trans*-acting factors (e.g. chromatin remodelers), but such region-specific effect is less likely. An alternative explanation is assembly error that misassembled mitochondrial reads into nuclear DNA sequence, which requires further FISH experiment or long-read nuclear sequences for validation. Regardless of the cause of hypersensitivity, this region was blacklisted and excluded from following ACR analyses in this study.

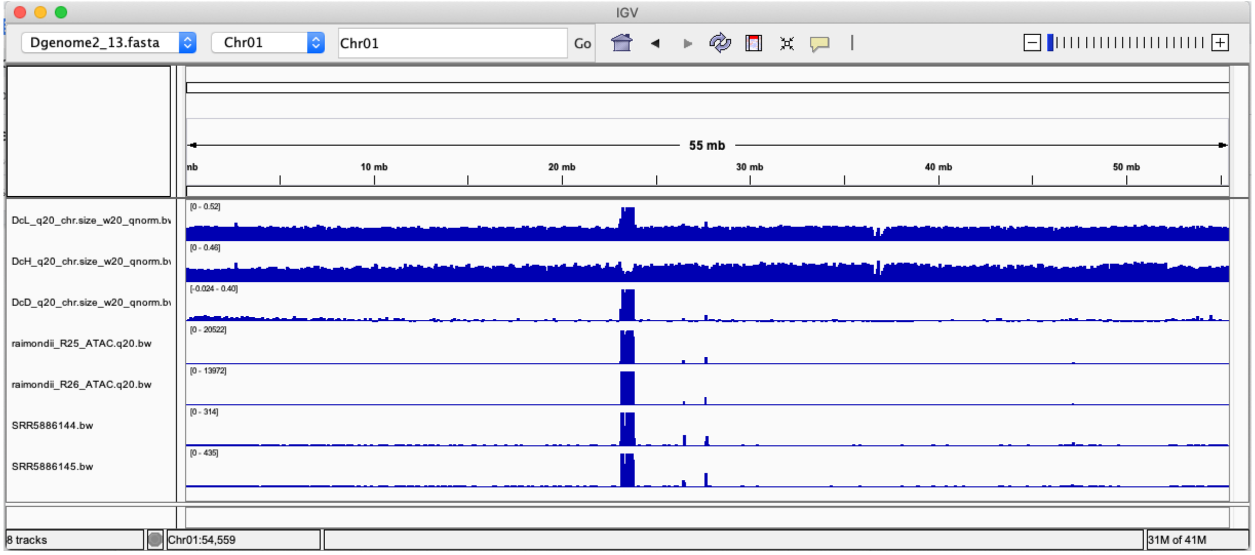

Supplementary Text 3 Figure 1. IGV visualization of *G. raimondii* chromosome 1. Tracks from top to bottom are: 1) nucleosome occupancy by light digestion; 2) nucleosome occupancy by heavy digestion; 3) DNS; 4) ATAC-seq rep 1; 5) ATAC-seq rep 2; 6) DNase-seq rep 1; 5) DNase-seq rep 2.

Supplementary Text 4: Comparison of different analytic approaches to conduct differential analysis (DA) analysis

Following a previously established workflow (Reske et al. 2020), we compared different approaches to conduct DNA analysis using the R package csaw [v1.16.1] (Lun and Smyth 2016). As shown in **Supplementary Text 4 Table 1**, methold 1 and 2 map MNase-seq read pairs to genomic regions of

interest (i.e., detected ACRs as a given peak set) for examining DA, while method 3 and 4 first map read pairs to sliding windows of the whole genome and then aggregate DA signals on regions of interest. For normalization, method 1 and 3 implement TMM (the trimmed mean of M values), while 2 and 4 implement a non-linear loess-based (loess: locally estimated scatterplot smoothing) normalization method. By contrasting MNase-seq profiles between light and heavy digestion, we expected the observed DA regions to recover ACRs detected by the iSeg [v1.3.4] (Girimurugan et al. 2018) application on DNS scores. In other words, we asked if those previously detected ACRs can be supported by DA analysis; the higher recovery rate of ACRs could be used to identify the more suitable DA method. Except method 2, the other three methods recovered 38-43% of ACRs (**Supplementary Text 4 Figure 1**), indicating that the previous ACR detection was well supported by the DA analysis using default *csaw* parameters. It is worth pointing out that the recovery rate can be affected by the stringency of both iSeg (i.e. Biological cutoff) and *csaw* (analytic parameters and FDR threshold). Because method 3 and 4 are more versatile than method 1 in analyzing other genomic regions of interest, such as gene promoters, and method 3 performs slightly better than method 4, we chose method 3 for following analyses.

To conduct DA analysis between diploids, F_1, and AD_1, we tested the use of both concatenated diploid (A_2+D_5) and AD_1 references to mitigate biases introduced by the reference genome. Because the mapping rates of MNase-seq data were higher when mapped to the corresponding reference than those mapped to the other reference (**Supplementary Text 4 Table 2**), the A_2+D_5 reference is more suitable for comparing diploids and F_1 accessibility profiles (i.e., inferring hybridization effect), while the AD_1 reference is more suitable for comparing diploids and AD_1 accessibility profiles (i.e., inferring polyploidization effect). The stronger polyploidization than hybridization effect was found regardless of which reference genome was used (**Supplementary Text 4 Figure 2**).

Supplementary Text 4 Table 1. Four analytic approaches tested

#	***csaw* mode**	**Normalization**	**Description**
1	*regionCounts()*	TMM	Only ACR regions were used. TMM assumes that most regions are not truly DA, and it assesses for systematic signal differences present across the genome that are presumed to be technical. Therefore, the TMM method should control for technical error more than scaling to total read depth by eliminating any systematic biases while still permitting true asymmetric differences.
2	*regionCounts()*	Loess	Only ACR regions were used. Loess assumes a symmetric global distribution in which there are no true biological global differences, and any evidence of these biases are technical and should be removed. This explains the low recovery and symmetric distribution of up and down changes.
3	*windowCounts()*	TMM	TMM was globally applied to binned windows of the whole genome, and the DA signals of the ACR regions were extracted for significance tests.
4	*windowCounts()*	Loess	Loess was globally applied to binned windows of the whole genome, and the DA signals of the ACR regions were extracted for significance tests.

Supplementary Text 4 Table 2. Mapping rate against different reference genomes

**MNase-seq sample**	**AD_1 reference**	**A_2+D_5 reference**
A6H	49.22%	**68.55%**
A6Hn	66.78%	**73.97%**
A6L	57.04%	**75.10%**
A6Ln	56.13%	**76.45%**
D1H	53.36%	**89.63%**
D1L	53.39%	**87.49%**
D2H	47.50%	**81.84%**
D2L	54.20%	**88.35%**
F2H	51.51%	**77.82%**
F2L	55.16%	**80.22%**
F3H	52.76%	**77.83%**
F3L	54.00%	**78.81%**
M1H	**84.49%**	57.00%
M1L	**85.30%**	57.47%
M2H	**84.11%**	56.12%
M2L	**84.68%**	57.81%

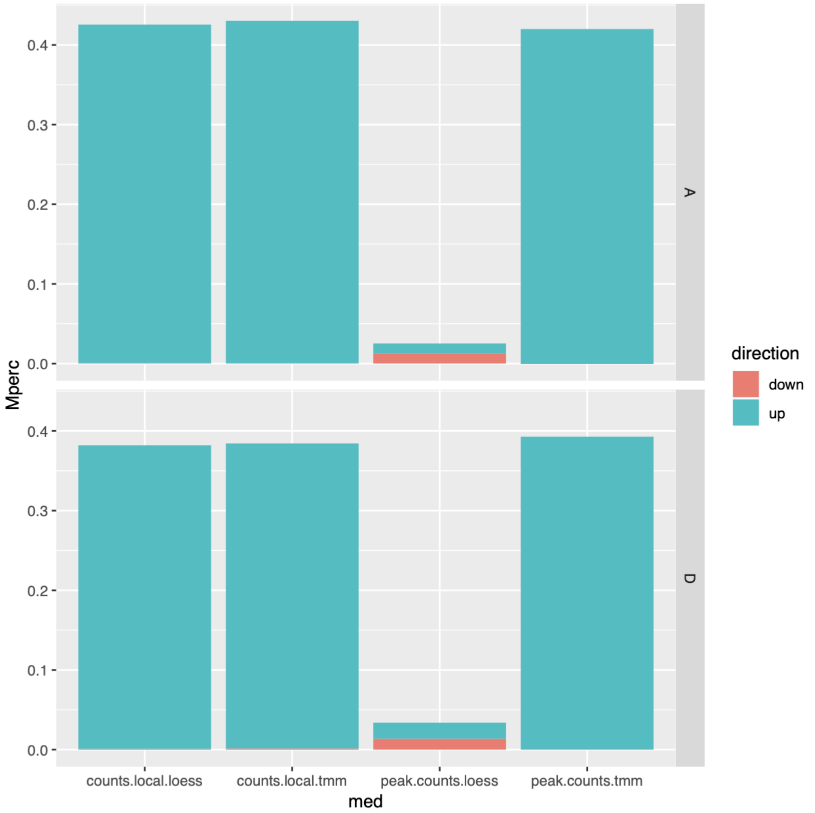

Supplementary Text 4 Figure 1. Comparison of four analytic approaches in identifying ACRs as DA between light and heavy conditions in AD_1. The up or down direction indicates higher mapping coverage in light (sensitive to light MNase digestion) or heavy (resistant to light digestion) condition, respectively. The A- and D- subgenome results were separately plotted with the y-axis representing the percentage recovery rate of ACRs.

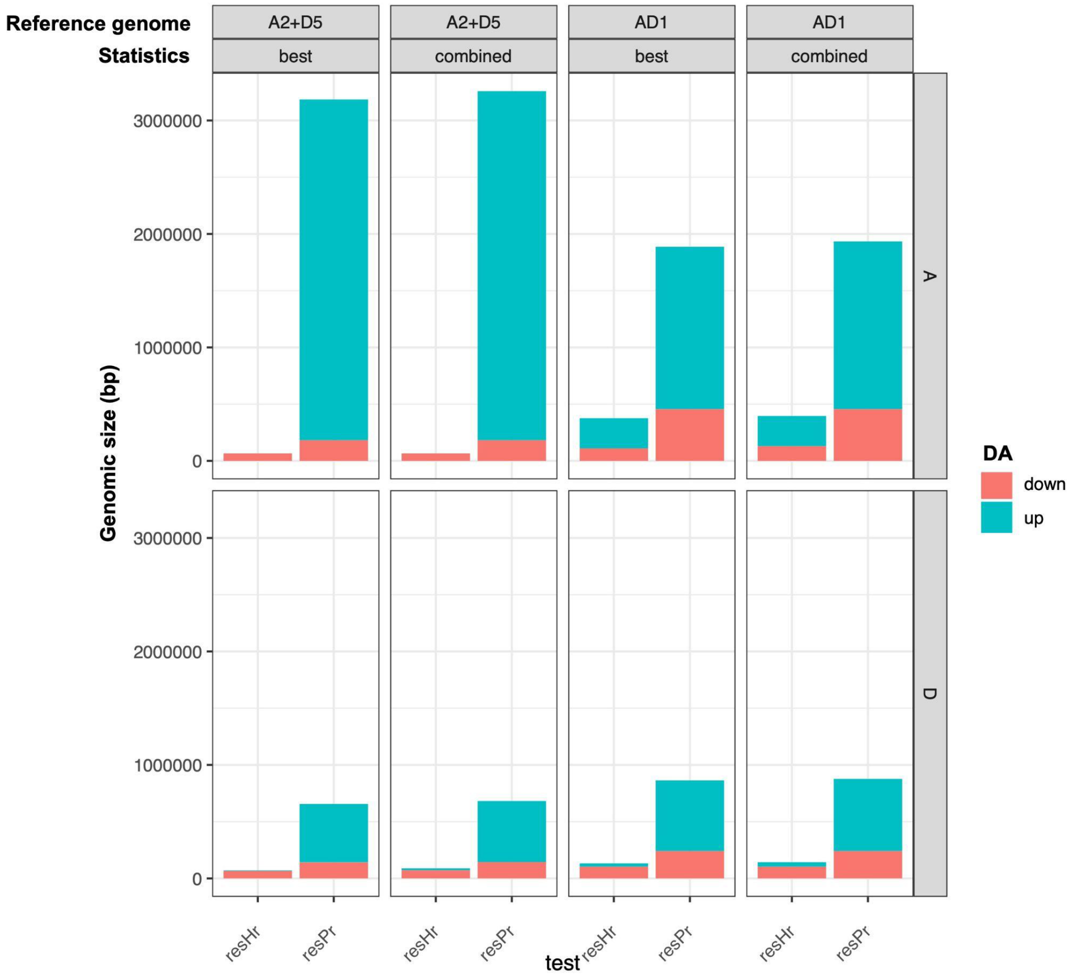

Supplementary Text 4 Figure 2. Comparison of DA results based on different reference genomes - A_2+D_5 and AD_1. In combination with each reference genome (top bar), two overlap statistics of *csaw* were used (combined and best; 2nd bar from top), and the A- and D- subgenome results were plotted below. Within each plot panel, the bars represent the detected genomic regions exhibiting DA of F_1 vs diploids and (resHr) AD_1 vs diploids (resPr); up- (blue) and down- (red) regulation (blue) represent increased and decreased accessibility, respectively, by genome evolution.

Supplementary Text 5: Analysis of duplicated gene expression patterns using different mapping strategies

Among the twelve RNA-seq samples generated, one D_5 sample turned out to be a mislabeled polyploid sample and was thereby excluded (**Supplementary Text 5 Figure 1**). To optimize the method to infer duplicated gene expression patterns, we tested three mapping strategies termed by (1) D_5-ref, (2) AD_1-ref, and (3) individual-ref. The mapping rates of diploid and F_1 RNA-seq reads were much higher using individual-ref and D_5-ref than those using AD_1-ref (80-90% vs 56-64%), while the mapping rates of AD_1 RNA-seq reads were comparable between different strategies (82-88%) (Table S12). It is reasonable to conclude that individual-ref and D_5-ref are more suitable than AD_1-ref to handle the interspecific analysis of diploid and allopolyploid cotton transcriptomes. It is also worth noting that both individual-ref and D_5-ref tend to map more D_5 than A_2 reads, while AD_1-ref tends to map more A_2 than D_5 reads, which can introduce technical bias in inferring expression asymmetry between A- and D- (sub)genomes. To mitigate the biases, we took a conservative approach to report the duplicated gene expression patterns that can be commonly identified by different mapping strategies. The commonly inferred *total* gene expression pattern of homoeologous gene pairs accounted for 37.2-67.8% of F_1/AD_1 differential expression relative to diploids (Table S13) and 62.0-74.8% of additivity test results (Table S14). The commonly inferred patterns based on partitioned homoeologous gene expression accounted for 41.5%-63.7% of parental divergence, 22.7-53.2% of HEB in F_1, 71.4-82.9% of HEB in AD_1, 15.9-61.9% of evolutionary impact, and 6.3-79.2% of *cis*-*trans* categories (**Supplementary Text 5 Table 1**).

Supplementary Text 5 Table 1. Inference of duplicated gene expression patterns

Regulation Pattern	common	D5-ref	D5-ref %	AD1-ref	AD1-ref %	Individual-ref	Invidual-ref %
Diploid divergence (***A*** ≠ 0)	2923	4591	63.7%	7043	41.5%	5280	55.4%
HEB in F_1 (***B*** ≠ 0)	1483	2788	53.2%	6538	22.7%	3627	40.9%
HEB in AD_1 (***Bp*** ≠ 0)	3232	3901	82.9%	4522	71.5%	4522	71.5%
Hybridization (***Hr*** ≠ 0)	142	328	43.3%	891	15.9%	809	17.6%
Allopolyploidy (***Pr*** ≠ 0)	1155	1867	61.9%	2821	40.9%	2649	43.6%
Genome doubling (***Wr*** ≠ 0)	679	1383	49.1%	2577	26.3%	2337	29.1%
Cis-trans category total	12645	22889	-	22889	-	22889	-
1.Cis only	849	1639	51.8%	3805	22.3%	1899	44.7%
2.Trans only	62	161	38.5%	215	28.8%	277	22.4%
3.Cis+Trans: enhancing	9	23	39.1%	33	27.3%	37	24.3%
4.Cis+Trans: compensating	8	35	22.9%	193	4.1%	119	6.7%
5.Compensatory	21	91	23.1%	432	4.9%	333	6.3%
6.Conserved	8913	15428	57.8%	11259	79.2%	14218	62.7%
7.Ambiguous	2783	5512	50.5%	6952	40.0%	6006	46.3%

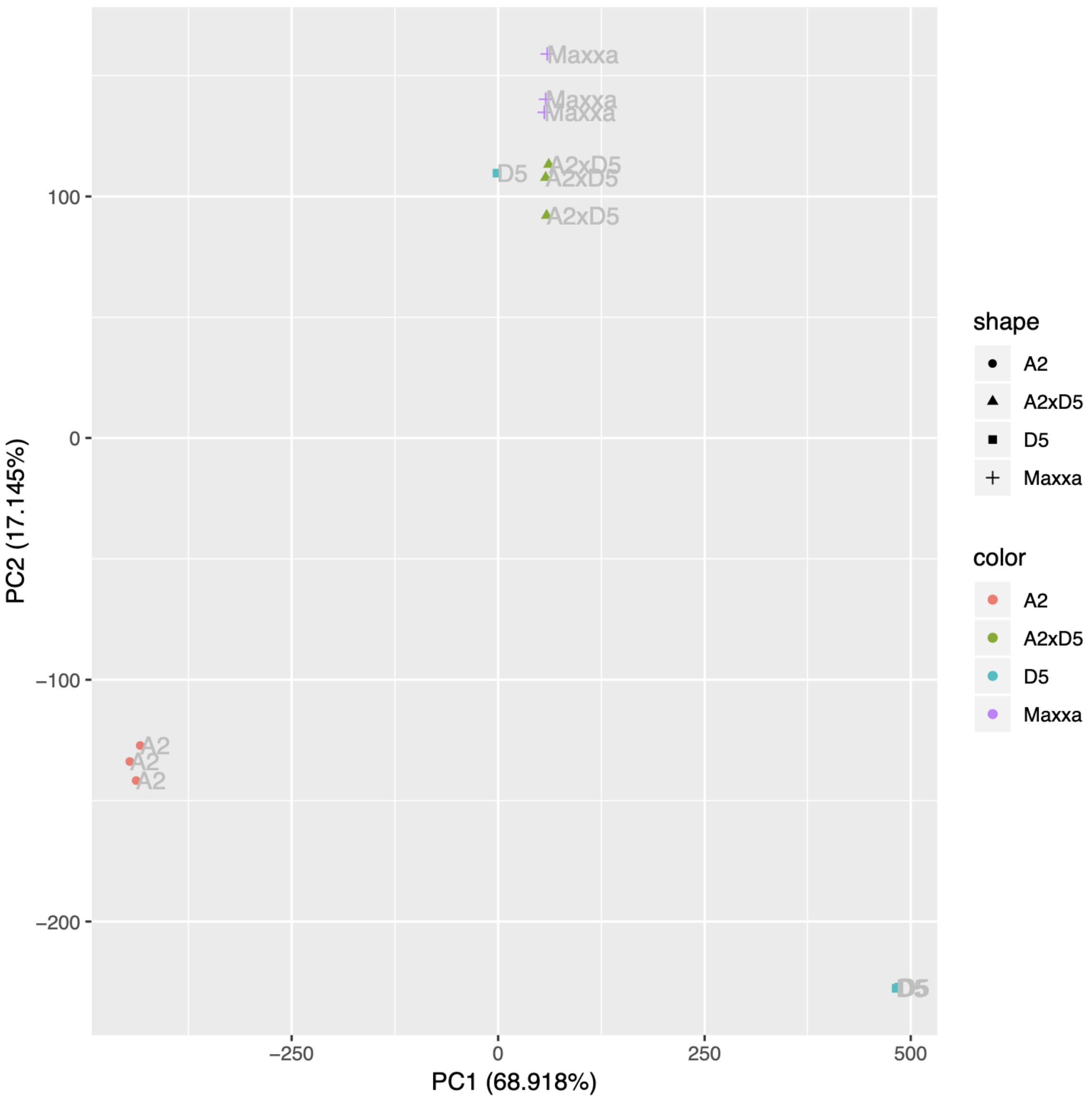

Supplementary Text 5 Figure 1. PCA analysis of log2 TPM using D_5-ref.

References

Girimurugan, S. B., Liu, Y., Lung, P.-Y., Vera, D. L., Dennis, J. H., Bass, H. W., & Zhang, J. (2018). iSeg: an efficient algorithm for segmentation of genomic and epigenomic data. In *BMC Bioinformatics* (Vol. 19, Issue 1). https://doi.org/10.1186/s12859-018-2140-3

Landolin, J. M., Johnson, D. S., Trinklein, N. D., Aldred, S. F., Medina, C., Shulha, H., Weng, Z., & Myers, R. M. (2010). Sequence features that drive human promoter function and tissue specificity. *Genome Research*, *20*(7), 890–898.

Lun, A. T. L., & Smyth, G. K. (2016). csaw: a Bioconductor package for differential binding analysis of ChIP-seq data using sliding windows. *Nucleic Acids Research*, *44*(5), e45.

Lu, Z., Marand, A. P., Ricci, W. A., Ethridge, C. L., Zhang, X., & Schmitz, R. J. (2019). The prevalence, evolution and chromatin signatures of plant regulatory elements. *Nature Plants*. https://doi.org/10.1038/s41477-019-0548-z

Paterson, A. H., Wendel, J. F., Gundlach, H., Guo, H., Jenkins, J., Jin, D., Llewellyn, D., Showmaker, K. C., Shu, S., Udall, J., Yoo, M.-J., Byers, R., Chen, W., Doron-Faigenboim, A., Duke, M. V., Gong, L., Grimwood, J., Grover, C., Grupp, K., … Schmutz, J. (2012). Repeated polyploidization of *Gossypium genomes* and the evolution of spinnable cotton fibres. *Nature*, *492*(7429), 423–427.

Reske, J. J., Wilson, M. R., & Chandler, R. L. (2020). ATAC-seq normalization method can significantly affect differential accessibility analysis and interpretation. *Epigenetics & Chromatin*, *13*(1), 22.

Ricci, W. A., Lu, Z., Ji, L., Marand, A. P., Ethridge, C. L., Murphy, N. G., Noshay, J. M., Galli, M., Mejía-Guerra, M. K., Colomé-Tatché, M., Johannes, F., Rowley, M. J., Corces, V. G., Zhai, J., Scanlon, M. J., Buckler, E. S., Gallavotti, A., Springer, N. M., Schmitz, R. J., & Zhang, X. (2019). Widespread long-range cis-regulatory elements in the maize genome. *Nature Plants*, *5*(12), 1237–1249.

Wang, J., Zhuang, J., Iyer, S., Lin, X., Whitfield, T. W., Greven, M. C., Pierce, B. G., Dong, X., Kundaje, A., Cheng, Y., Rando, O. J., Birney, E., Myers, R. M., Noble, W. S., Snyder, M., & Weng, Z. (2012). Sequence features and chromatin structure around the genomic regions bound by 119 human transcription factors. In *Genome Research* (Vol. 22, Issue 9, pp. 1798–1812). https://doi.org/10.1101/gr.139105.112
