# Supplementary material for: Evolutionary Dynamics of Chromatin Structure and Duplicate Gene Expression in Diploid and Allopolyploid Cotton
Source: Mol Biol Evol. 2024 May 17;41(5):msae095. doi: 10.1093/molbev/msae095 (PMC11140268; doi:10.1093/molbev/msae095)
Supplement: msae095_Supplementary_Data [file msae095_supplementary_data.zip › 3.Supplementary Figures and Tables Legend.revision.pdf]

Supplementary Figures

Figure S1. Aggregate plots of chromatin features profiled by DNS-seq. For each species, genome-wide nucleosome occupancy by heavy (NO_H) and light (NO_L) MNase digestion, differential nuclease sensitivity (DNS), and subnucleosomal particle occupancy (SPO) were plotted over scaled gene regions. The scaled gene region encompasses 1.5 kb upstream of the transcription start site (TSS), the metagene scaled to 3 kb, and 1.5 kb downstream of the transcription end site (TES). Gene groups Q1 to Q4 represent the increasing expression quantiles, and Q0 represents the group of non-expressed genes.

Figure S2. Aggregate plots of chromatin features profiled by DNS-seq, ATAC-seq, and DNase-seq in *G. raimondii*. **A**. Genomic regions around TSS and TES of genes were plotted for DNS-seq (MSF.bc6.0 and SPO.bc5.0), ATAC-seq in this study ATAC.genrich and by You et al. (2022), and DNaseq by Wang et al. (2018) and by Han et al. (2022). **B**. Gene groups Q0-Q4 were categorized using young leaf expression data.

Figure S3. Comparing various chromatin profiles in *G. raimondii*. **A**. PCA analysis and eigenvalues. B. Heatmap and clustering based on Pearson's correlation coefficients. ATAC-seq and DNase-seq were most correlated with each other (Pearson's $r = 0.63$-0.84), followed by DNS versus SPO ($r = 0.53$-0.69). Despite the tissue difference, SPO signals were also well correlated with the DNase-seq results ($r = 0.46$-0.56). The lowest correlation was observed between DNS and ATAC-seq ($r = 0.12$-0.26).

Figure S4. Phasograms of nucleosomes in diploid, hybrid, and allopolyploid cotton. Phasograms are histograms of distances between the midpoints of neighboring nucleosome cores (i.e., center of paired-end reads), which reveal consistent spacing of positioned nucleosomes by exhibiting a wave-like pattern with a period that represents genome-average inter-nucleosomal spacing. The x-axis shows the range of recorded phases in base pair (bp). The y-axis shows frequencies of corresponding phases by read counts. Inset presents a linear fit to the positions of the phase peaks, where the slope represents the estimated nucleosome repeat length (NRL). For example, in the upper left panel, the NRL was estimated 198 bp in A_2.

Figure S5. XSTREME *de novo* motif discovery. A total of 184 motifs were enriched in 1 kb promoter pACRs: $A_2 = 25$, $D_5 = 36$; F_1:At = 3, F_1:Dt = 28; AD_1:At = 47, AD_1:Dt = 45. A global hierarchical tree was built by RSAT matrix-clustering for visualization and to inspect redundancy between similar motifs, which separated 184 motifs into 48 clusters. Each cluster has a different branch color from neighboring clusters.

Figure S6. Distribution of TEs relative to transcription start sites in A- and D- cotton genomes.

Figure S7. Sizes of TE superfamilies in different genomic regions.

Figure S8. Heatmap and clustering of expressed TE families.

Figure S9. Promoter accessibility of all, OG, and nonOG genes in diploid and allopolyploid cottons. Aggregation plots of DNS signals around TSS were present in diploids (top row), F_1 (middle two rows), and AD_1 (bottom row).

Figure S10. A- and D-genome promoter accessibility based on A_2 and D_5 references, respectively.

Figure S11. A- and D-genome promoter accessibility based on AD_1 references, respectively. **A.** HEB and impacts of genome evolution. **B.** nonadditive expression in F_1. **C**. nonadditive expression in AD_1.

Figure S12. Aggregation plots of chromatin accessibility signals by analyzing a public DNase-seq data (Han et al. 2022) as Figure 7A (**A**) ad Figure S9 (**B**).

Figure S13. Barplot of histone gene expression by variants.

Figure S14. Genomic tracks illustration of representative genes of histone variants

Supplementary Tables

Table S1. Summary of sequencing data in this study

Table S2. Summary of MNase-seq data

Table S3. Nucleosome identification and classification

Table S4. Summary of ATAC-seq and DNase-seq data in *G. raimondii*

Table S5. Comparison of ACR identification in *G. raimondii*

Table S6. Overlaps of ACR identification in *G. raimondii*

Table S7. Estimation of nucleosome repeat length (bp) by chromosome

Table S8. ACR identification by differential nuclease sensitivity analysis (BC=6.0) and subnucleosomal occupancy analysis (BC=4.0) in *G.arboreum*

Table S9. ACR identification by differential nuclease sensitivity analysis (BC=6.0) and subnucleosomal occupancy analysis (BC=5.0) in *G.raimondii*

Table S10. ACR identification by differential nuclease sensitivity analysis (BC=6.0) and subnucleosomal occupancy analysis (BC=4) in *G.hirsutum*

Table S11. ACR identification by differential nuclease sensitivity analysis (BC=6.0) and subnucleosomal occupancy analysis (BC=4.5) in *G. arboreum* X *G. raimondii*

Table S12. Ranking of significantly enriched motifs in 1 kb promoter ACRs

Table S13. Genome-wide TE classification and composition

Table S14. TE-derived ACR distribution and composition

Table S15. Mapping summary of RNA-seq data

Table S16. Analysis of expression asymmetry relative to diploids

Table S17. Test of additivity hypothesis

Table S18. Duplicated gene expression patterns under the *cis* and *trans* analytic framework

Table S19. Genome-wide characterization of cotton histone protein coding genes

Table S20. Histone gene expression analysis
