# Supplementary material for: Evolutionary Dynamics of Chromatin Structure and Duplicate Gene Expression in Diploid and Allopolyploid Cotton
Source: Mol Biol Evol. 2024 May 17;41(5):msae095. doi: 10.1093/molbev/msae095 (PMC11140268; doi:10.1093/molbev/msae095)
Supplement: msae095_Supplementary_Data [file msae095_supplementary_data.zip › 4.SuppleFigures.revision.pdf]

Figure S1

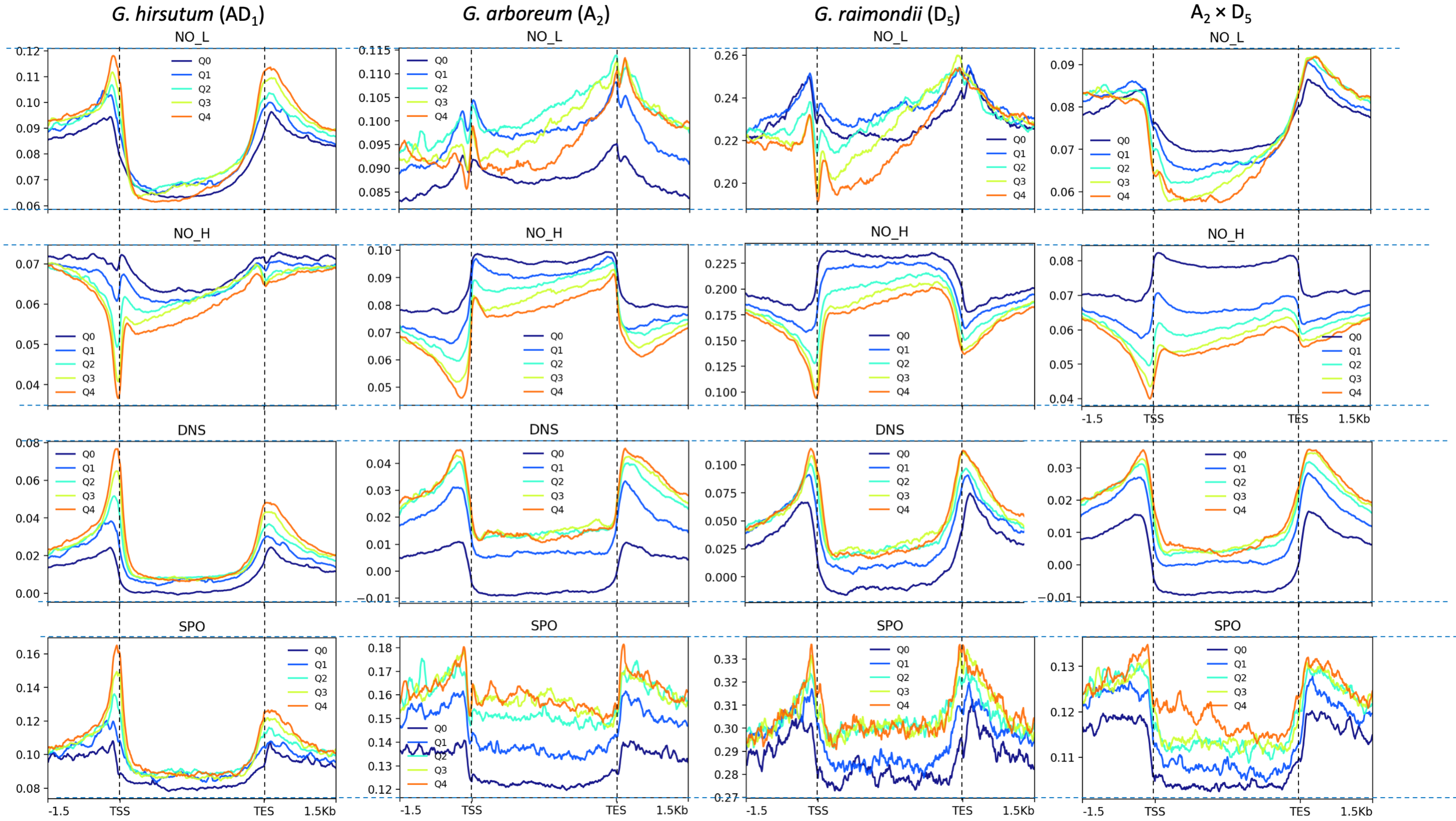

Figure S1. Aggregate plots of chromatin features profiled by differential sensitivity MNase-seq. For each species, genome-wide nucleosome occupancy by heavy (NO_H) and light (NO_L) MNase digestion, differential nuclease sensitivity (DNS), and subnucleosomal particle occupancy (SPO) were plotted over scaled gene regions. The scaled gene region encompasses 1.5 kb upstream of the transcription start site (TSS), the metagene scaled to 3 kb, and 1.5 kb downstream of the transcription end site (TES). Gene groups Q1 to Q4 represent the increasing expression quantiles, and Q0 represents the group of non-expressed genes.

Figure S2

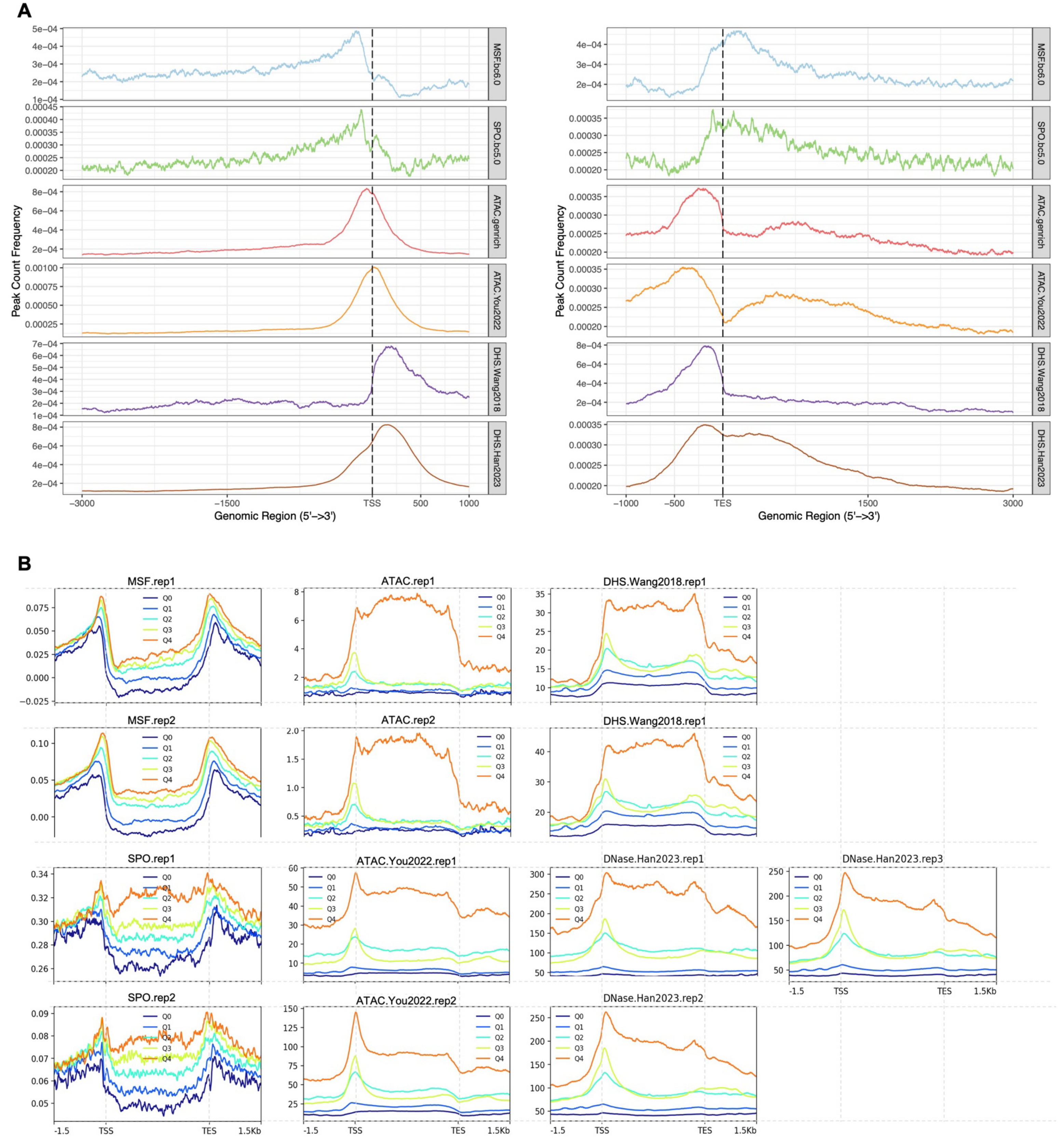

Figure S2. Aggregate plots of chromatin features profiled by DNS-seq, ATAC-seq, and DNase-seq in G. raimondii. A. Genomic regions around TSS and TES of genes were plotted for DNS-seq (MSF.bc6.0 and SPO.bc5.0), ATAC-seq in this study and by You et al. (2022), and DNaseq by Wang et al. (2018) and by Han et al. (2022). B. Gene groups Q0-Q4 were categorized using young leaf expression data.

Figure S3

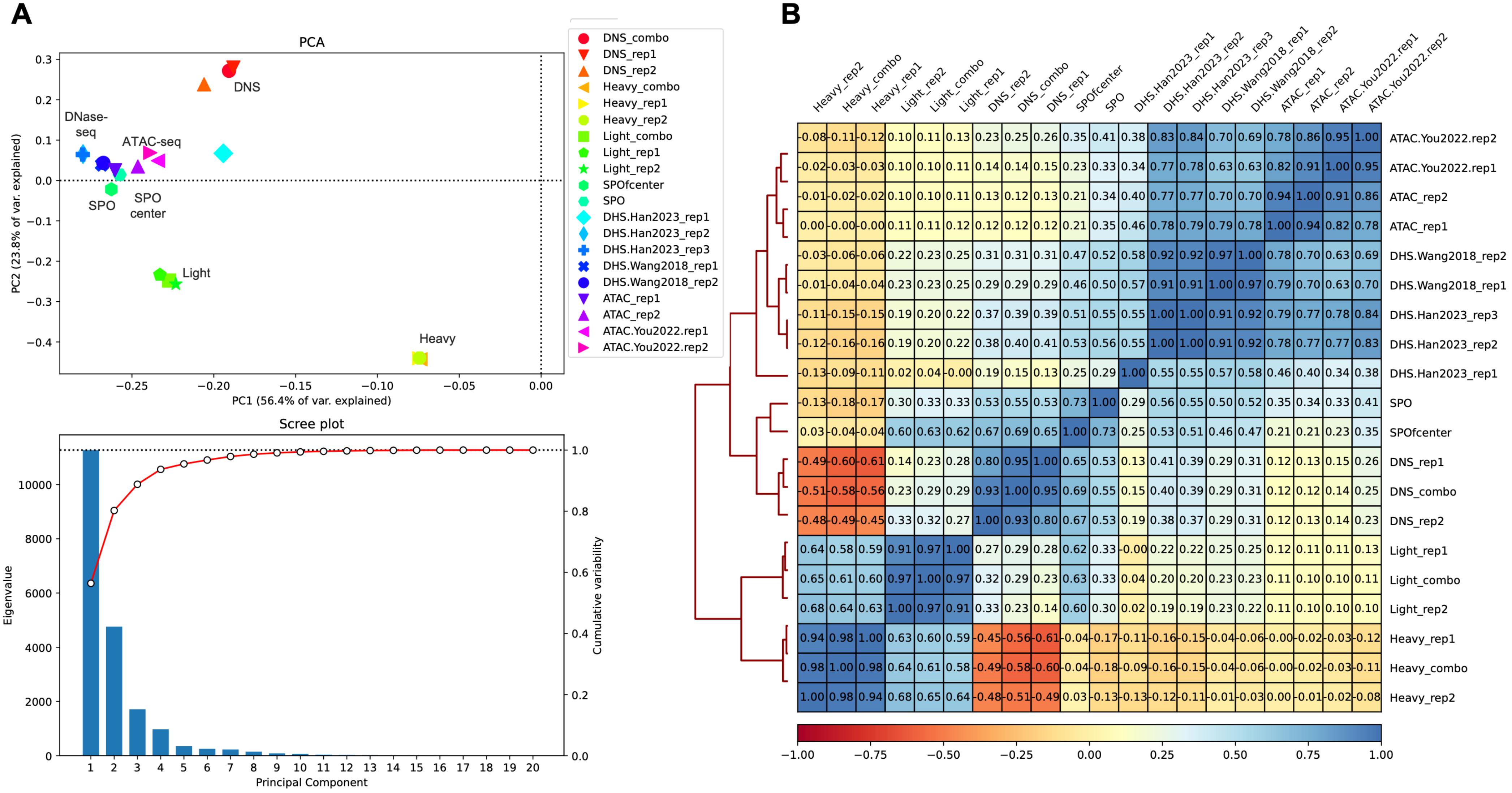

Figure S3. Comparing various chromatin profiles in G. raimondii. A. PCA analysis and eigenvalues. B. Heatmap and clustering based on Pearson's correlation coefficients. ATAC-seq and DNase-seq were most correlated with each other (Pearson's r = 0.63-0.84), followed by DNS versus SPO (r = 0.53-0.69). Despite the tissue difference, SPO signals were still well correlated with the DNase-seq results (r = 0.46-0.56). The lowest correlation was observed between DNS and ATAC-seq (r = 0.12-0.26).

Figure S4

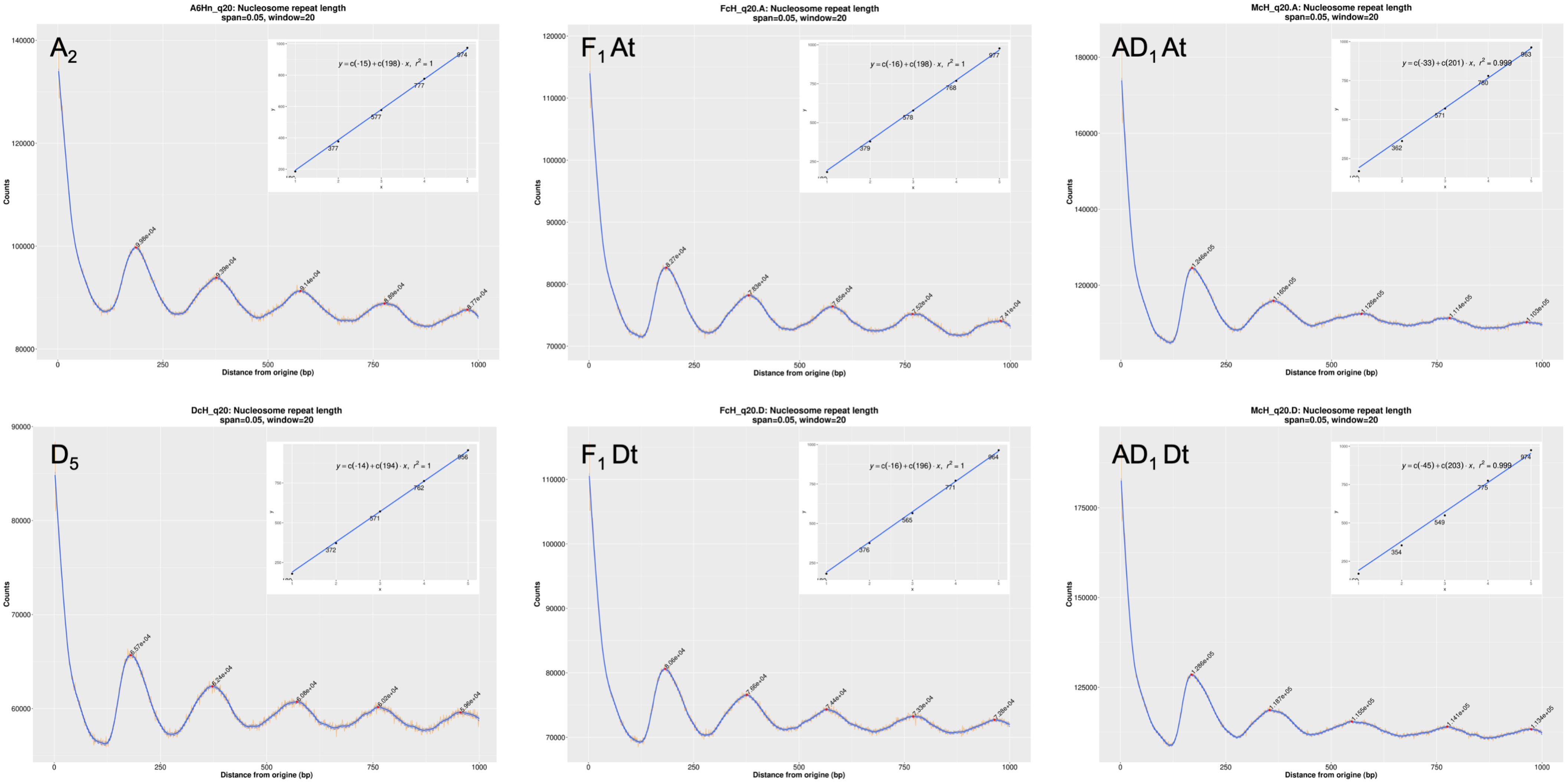

Figure S4. Phasograms of nucleosomes in diploid, hybrid, and allopolyploid cotton. Phasograms are histograms of distances between the midpoints of neighboring nucleosome cores (i.e., center of paired-end reads), which reveal consistent spacing of positioned nucleosomes by exhibiting a wave-like pattern with a period that represents genome-average internucleosomal spacing. The x-axis shows the range of recorded phases in base pair (bp). The y-axis shows frequencies of corresponding phases by read counts. Inset presents a linear fit to the positions of the phase peaks, where the slope represents the estimated nucleosome repeat length (NRL). For example, in the upper left panel, the NRL was estimated 198 bp in A2.

Figure S5

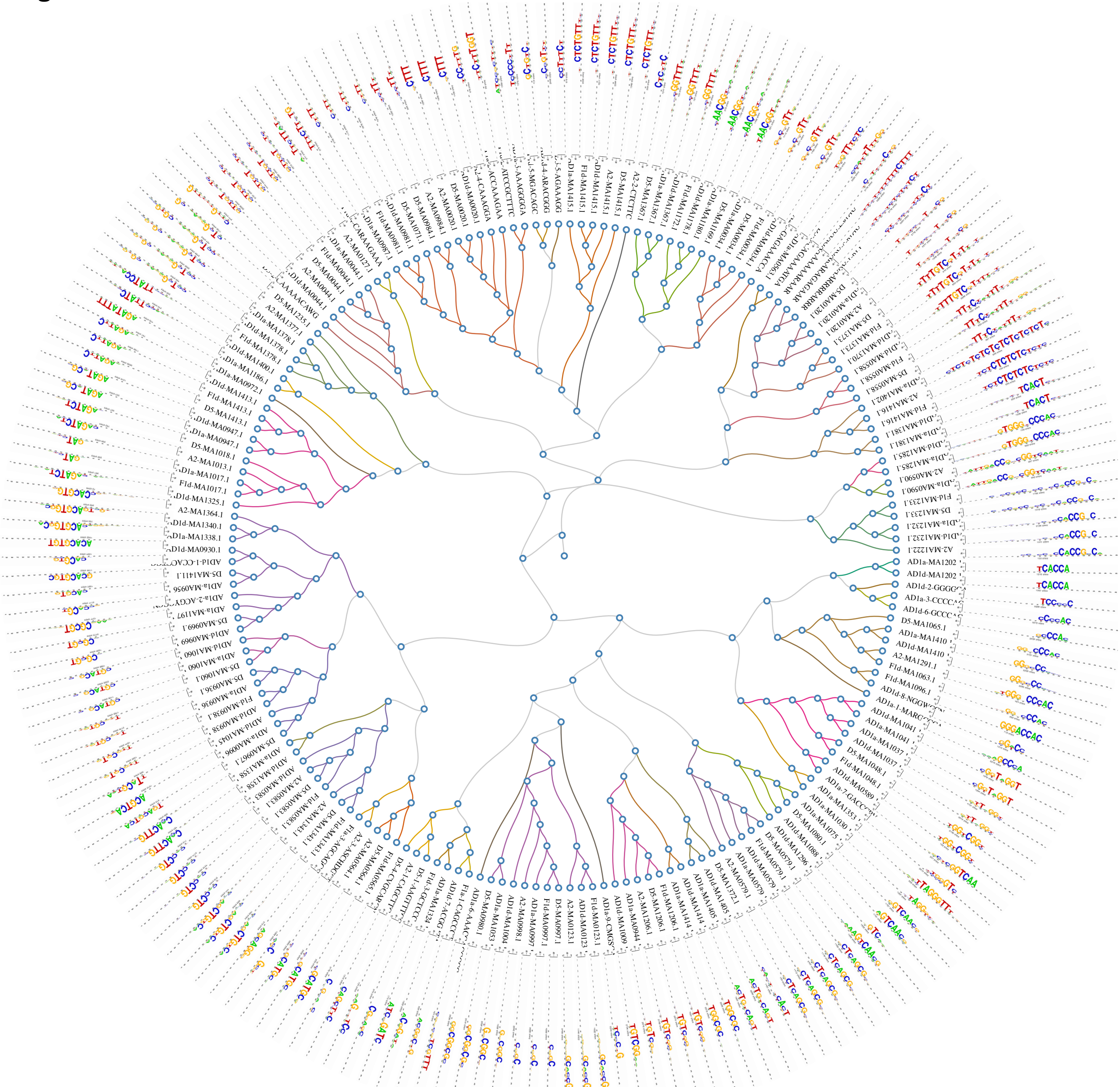

Figure S5. XSTREME de novo motif discovery. A total of 184 motifs were enriched in 1 kb promoter pACRs: A2 = 25, D5 = 36; F1:At = 3, F1:Dt = 28; AD1:At = 47, AD1:Dt = 45. A global hierarchical tree was built by RSAT matrix-clustering for visualization and to inspect redundancy between similar motifs, which separated 184 motifs into 48 clusters. Each cluster has a different branch color from neighboring clusters.

Figure S6

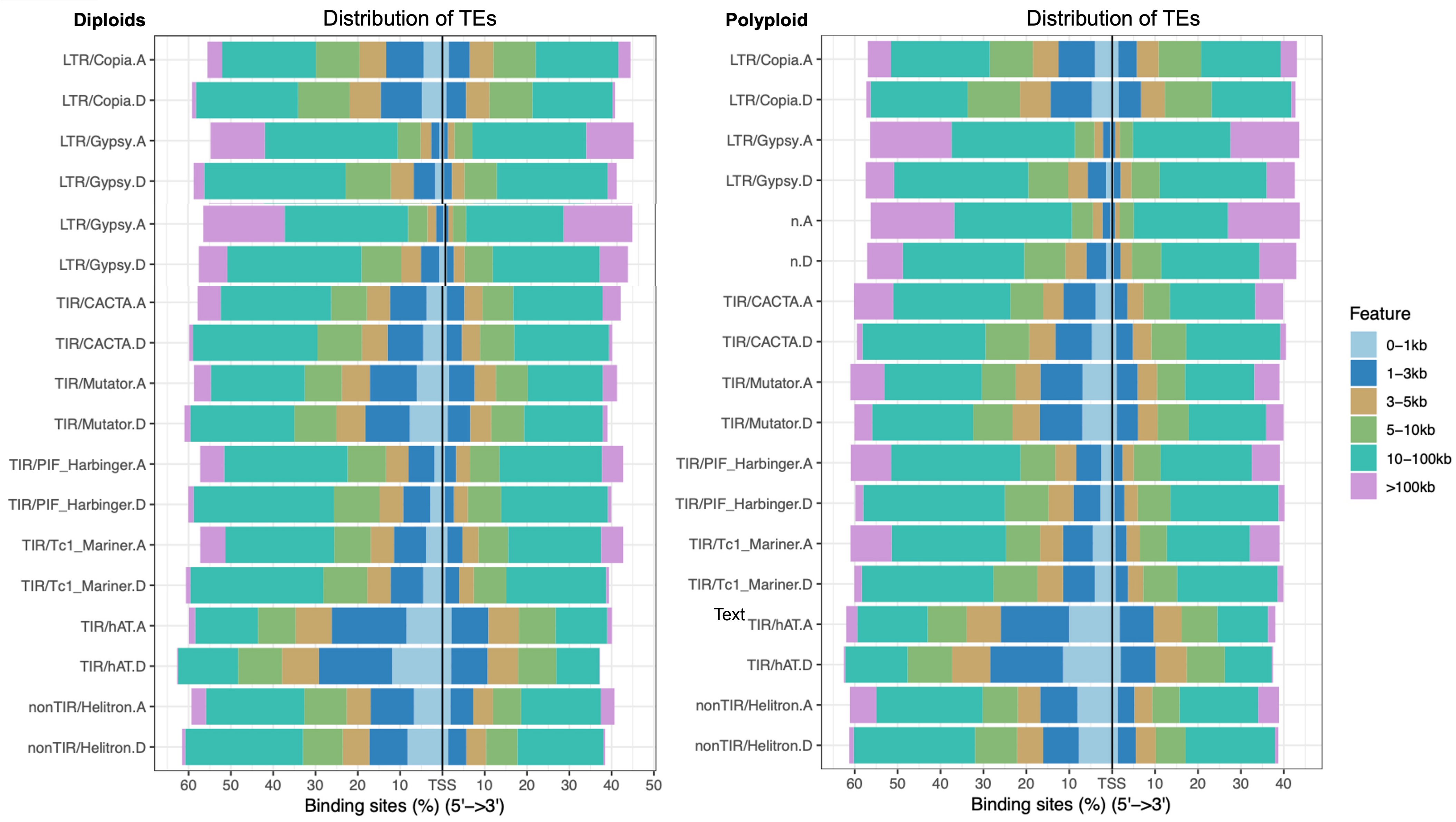

Figure S6. Distribution of TEs relative to transcription start sites in A- and D- cotton genomes.

Figure S7

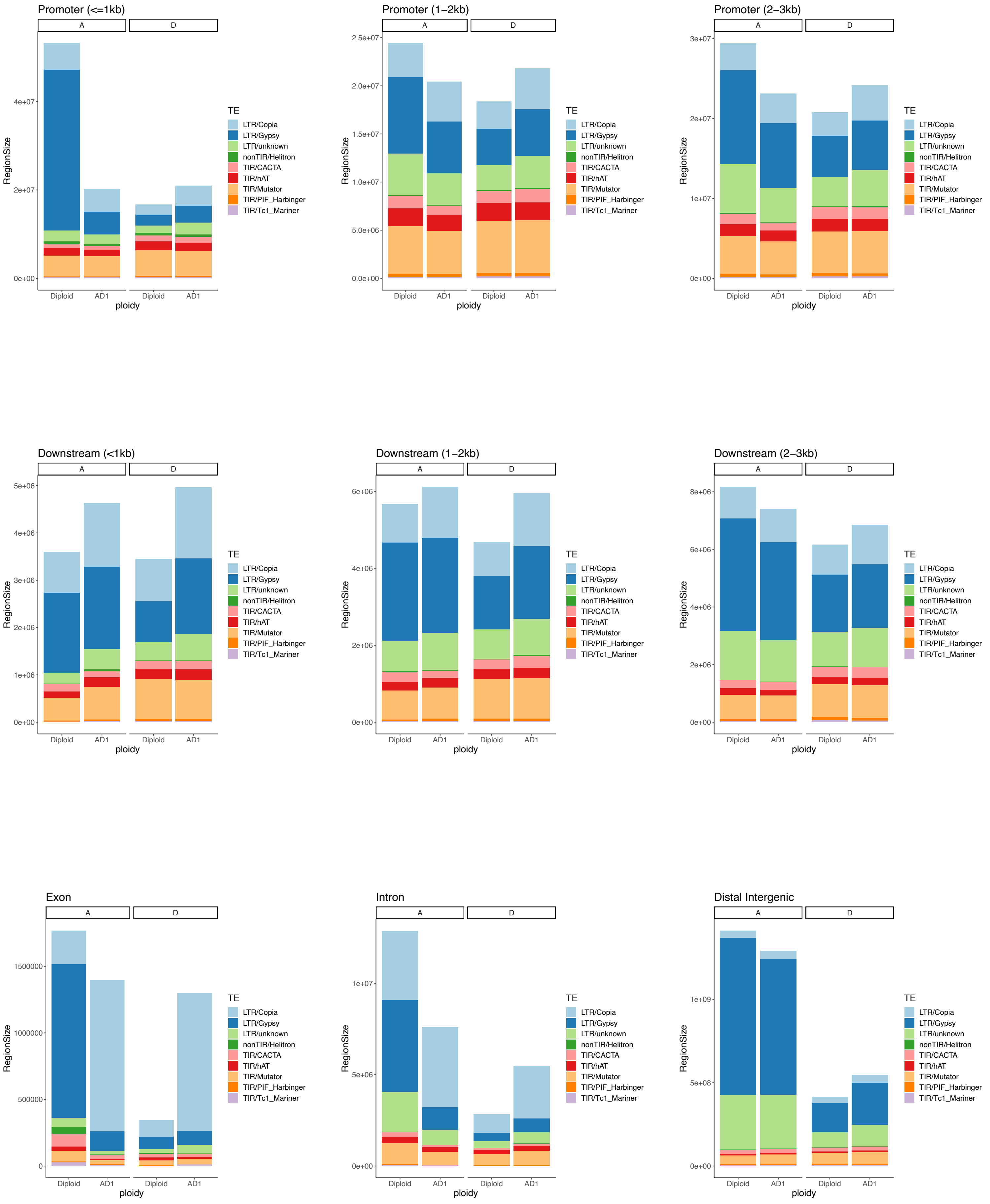

Figure S7. Sizes of TE superfamilies in different genomic regions.

Figure S8

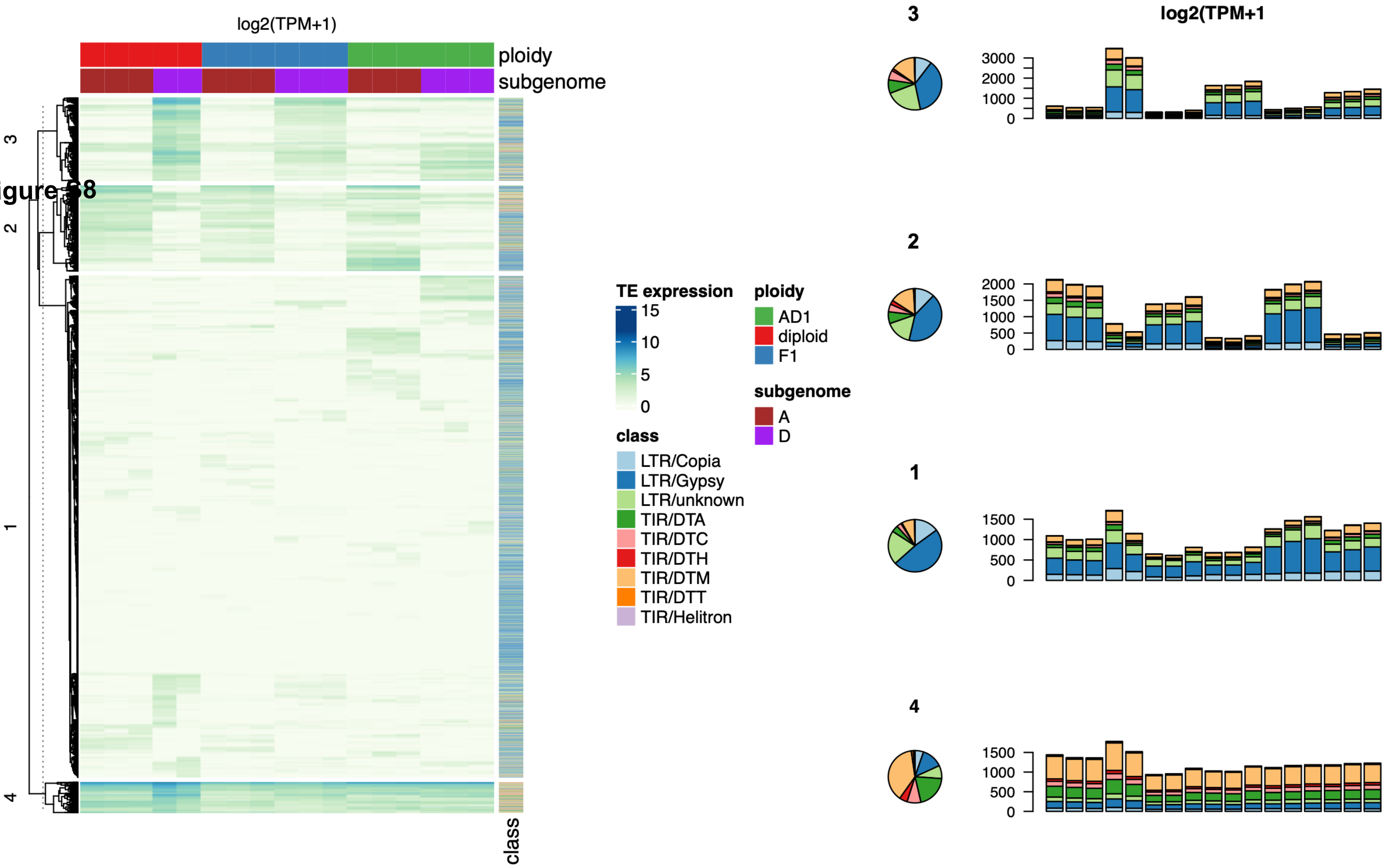
log2(TPM+1)
ploidy
subgenome
3
2
1
4
class
TE expression
15
10
5
0
ploidy
AD1
diploid
F1
subgenome
A
D
class
LTR/Copia
LTR/Gypsy
LTR/unknown
TIR/DTA
TIR/DTC
TIR/DTH
TIR/DTM
TIR/DTT
TIR/Helitron
log2(TPM+1
3000
2000
1000
1500
500
0

Figure S9

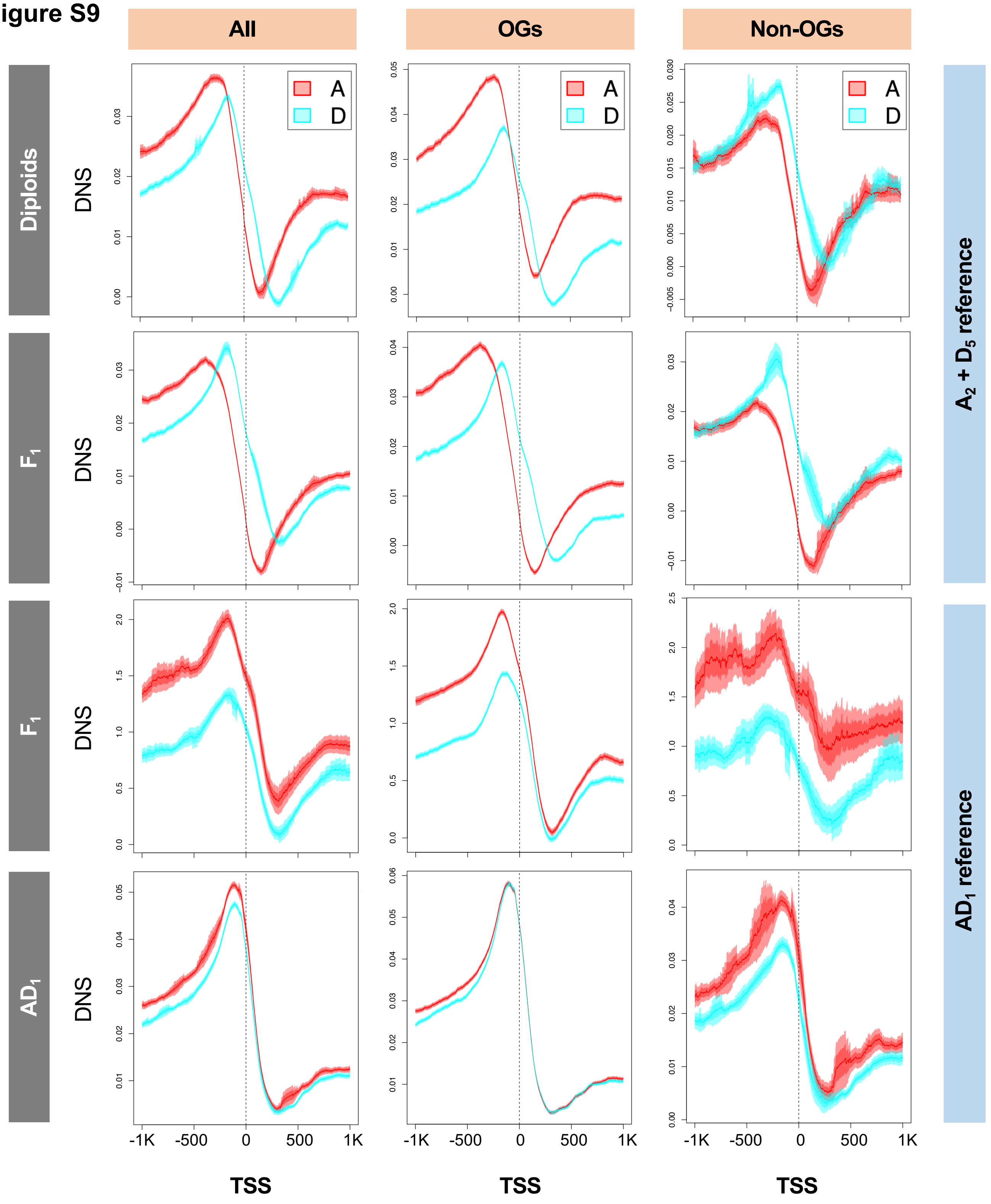

Figure S9. Promoter accessibility of all, OG, and nonOG genes in diploid and allopolyploid cottons. Aggregation plots of DNS signals around TSS were present in diploids (top row), F1 (middle two rows), and AD1 (bottom row).

Figure S10. p1/2

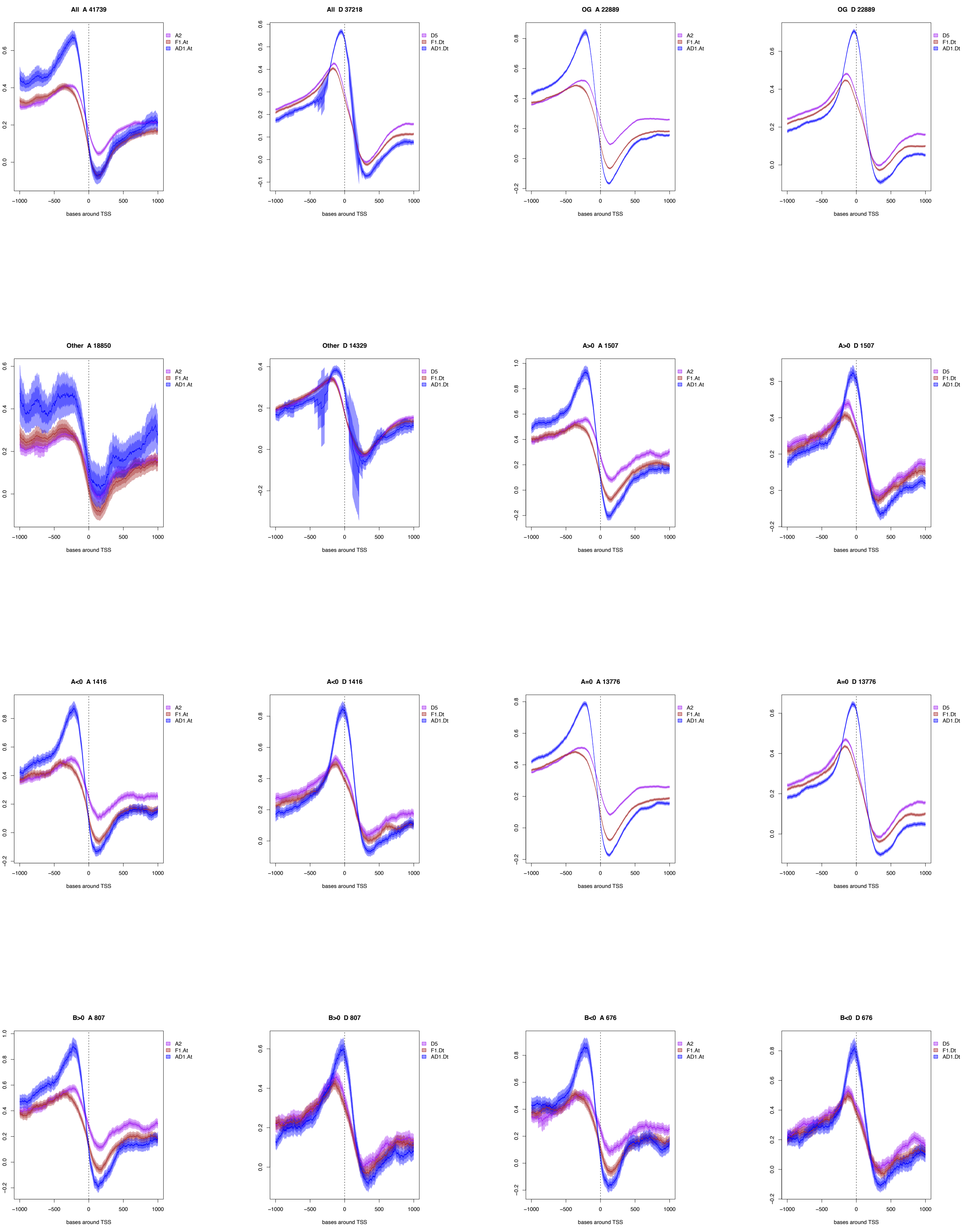

Figure S10. p2/2

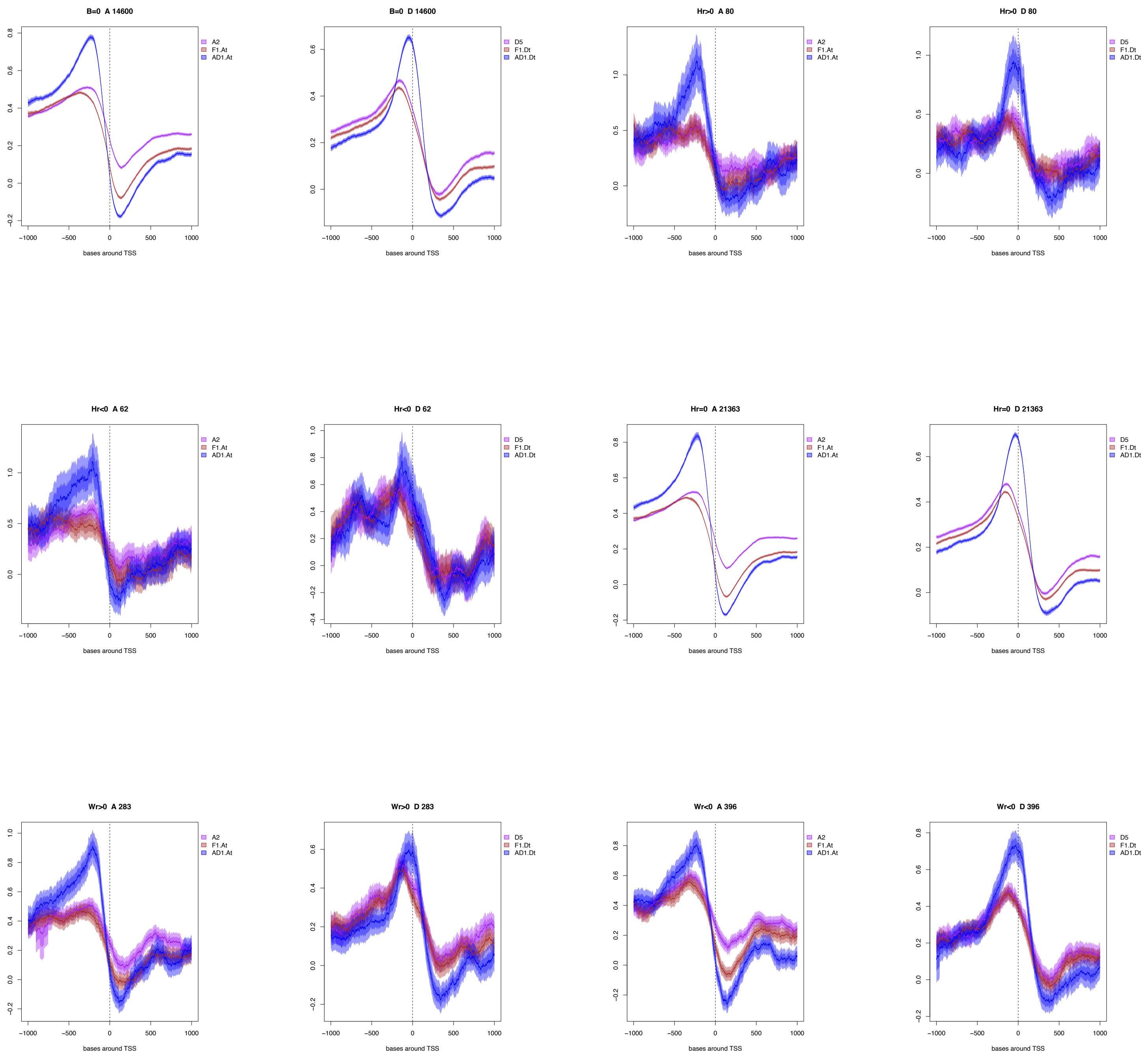

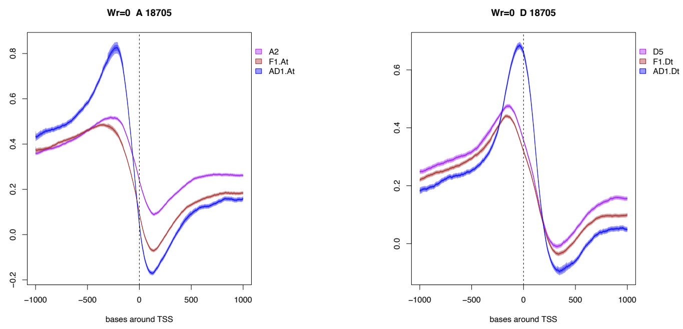

Figure S10. A- and D-genome promoter accessibility based on A2 and D5 references, respectively.

Figure S11A. p1/3

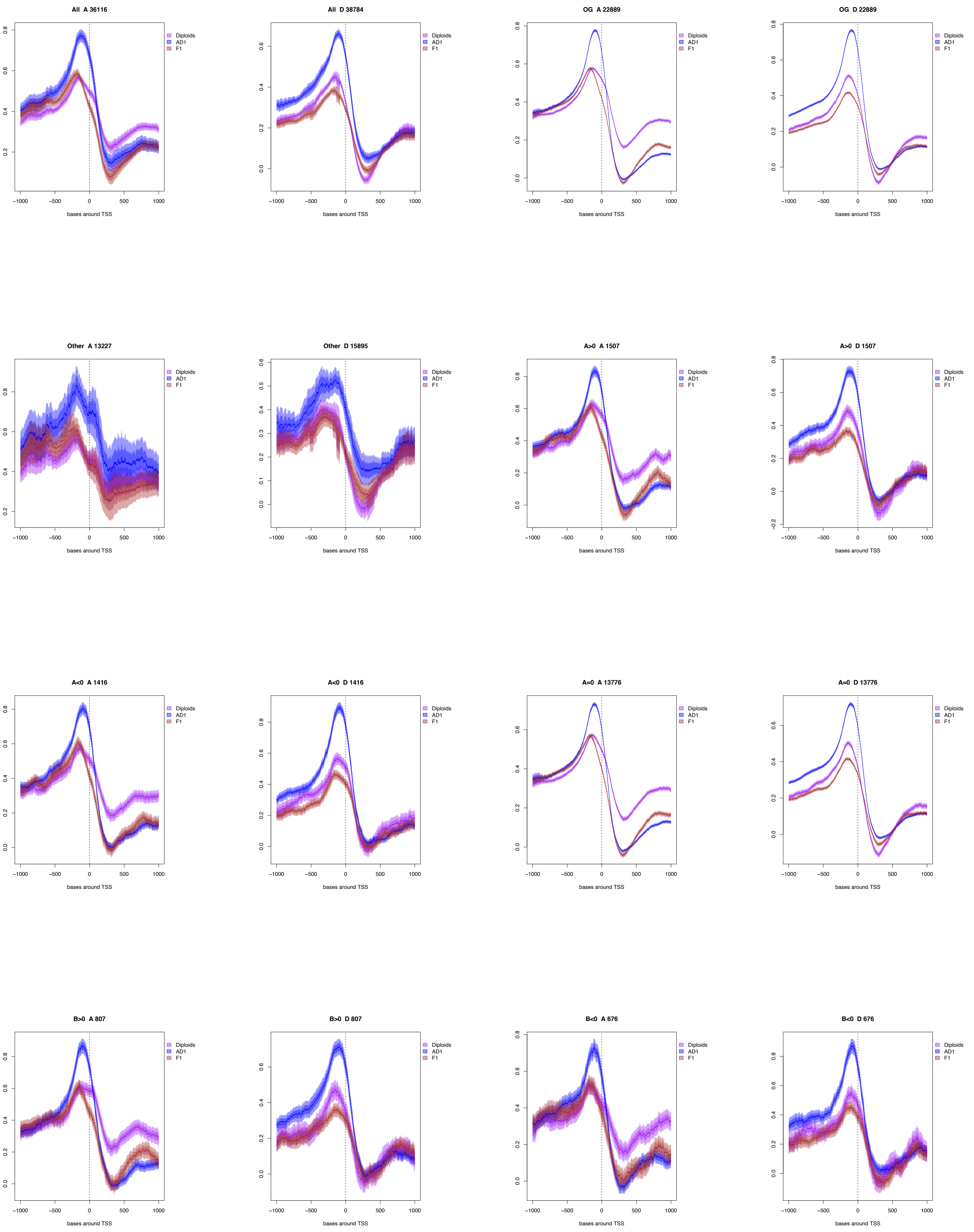

Figure S11A. p2/3

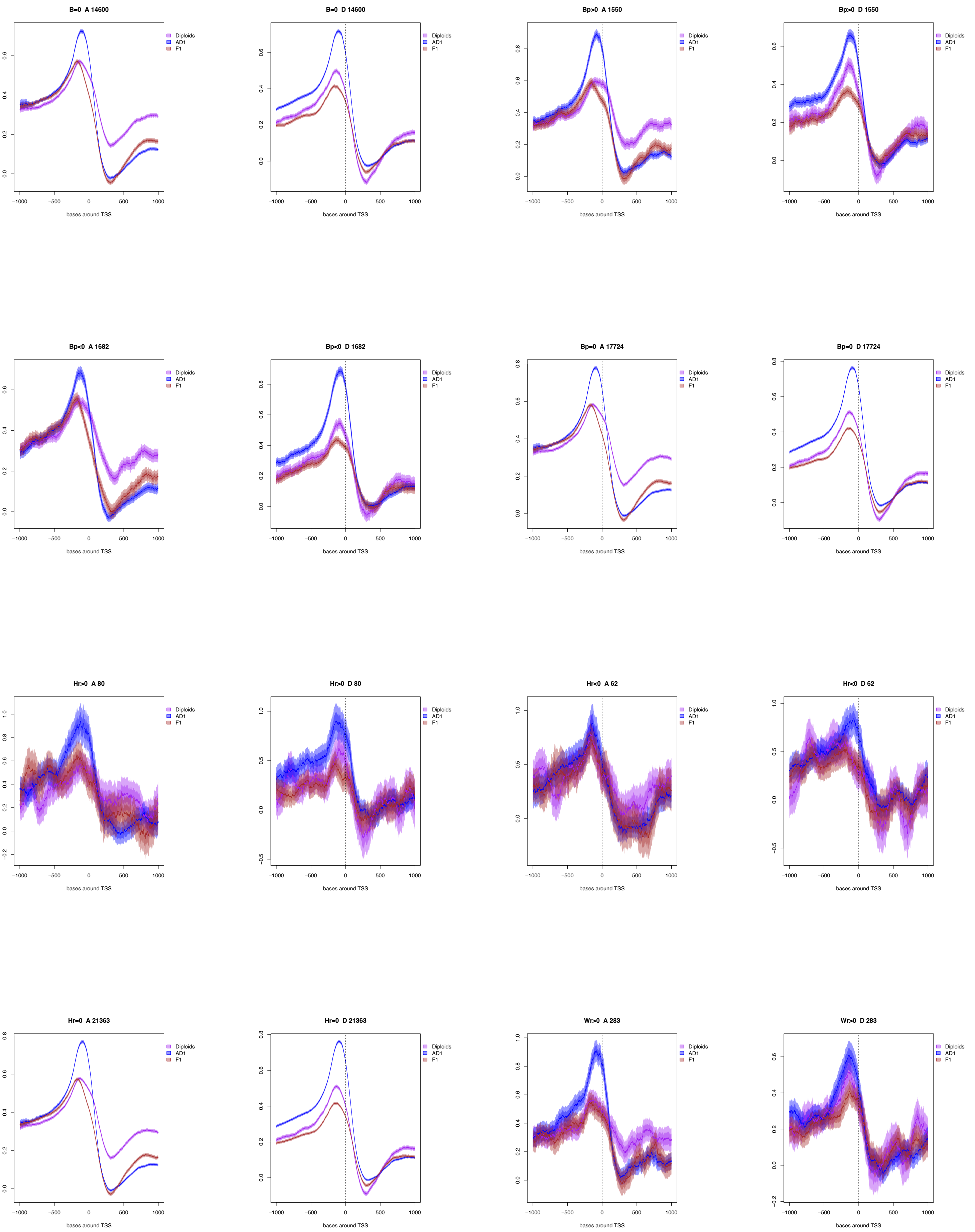

Figure S11A. p3/3

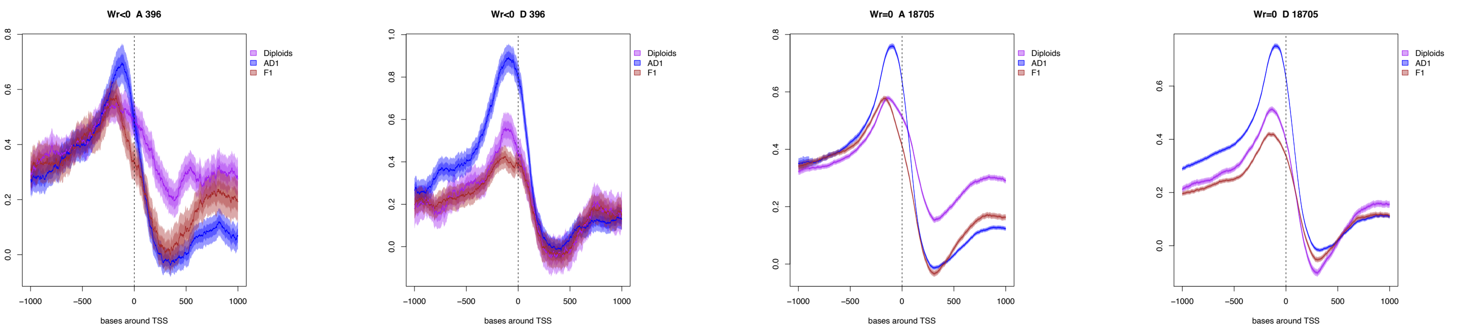

Figure S11. A- and D-genome promoter accessibility based on AD1 references, respectively.

A. HEB and impacts of genome evolution.

B. nonadditive expression in F1.

C. nonadditive expression in AD1.

Figure S11B

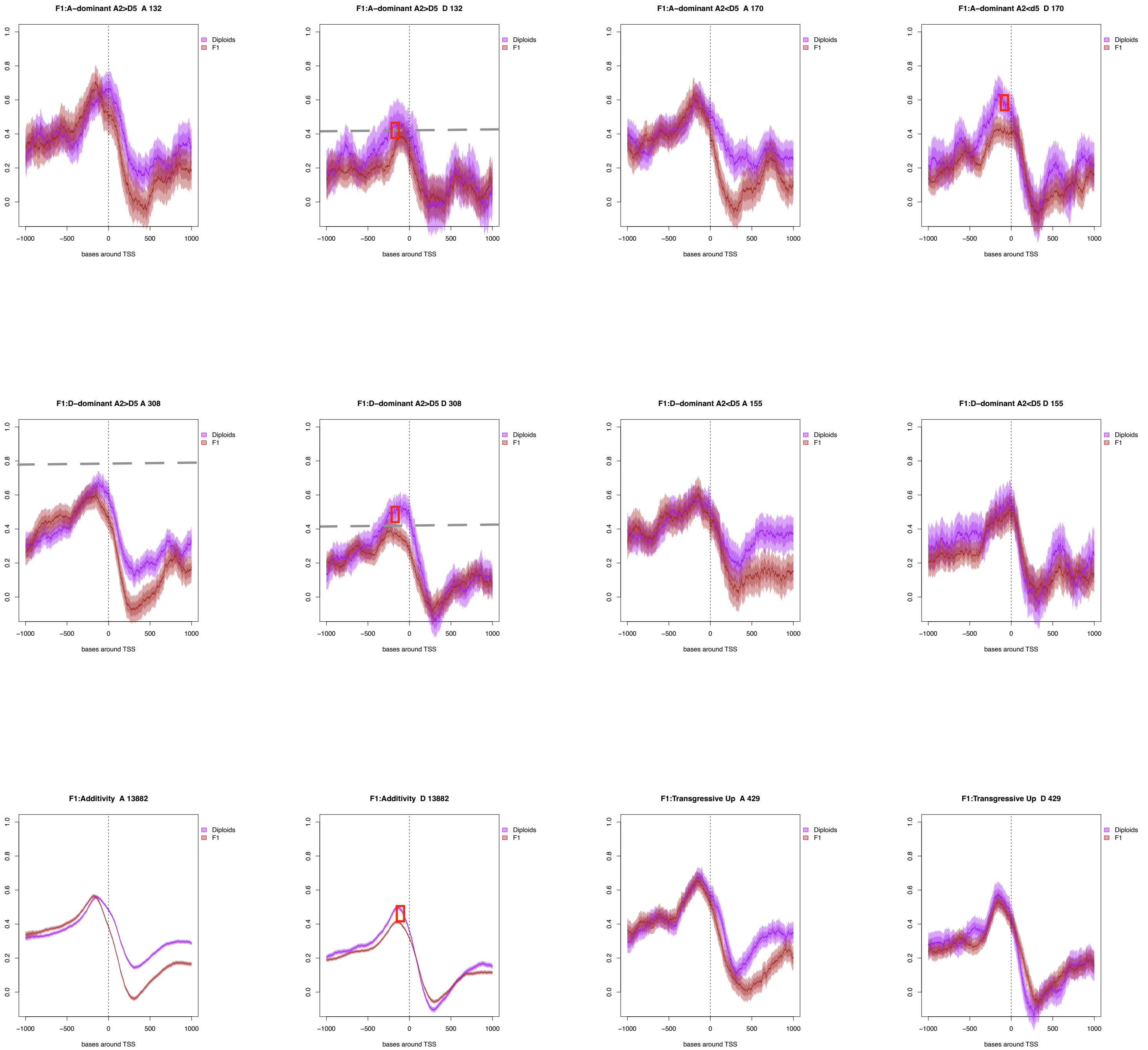

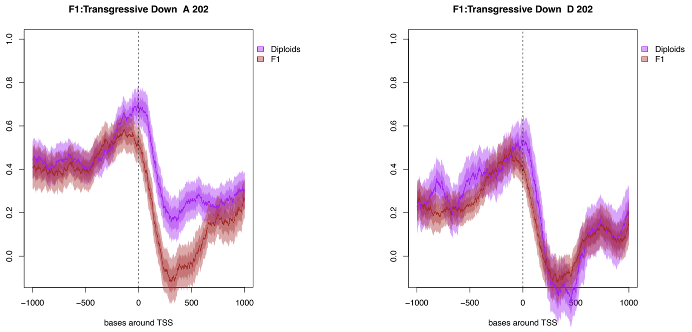

Figure S11. A- and D-genome promoter accessibility based on AD1 references, respectively.
A. HEB and impacts of genome evolution.
B. nonadditive expression in F1.
C. nonadditive expression in AD1.

Figure S11C

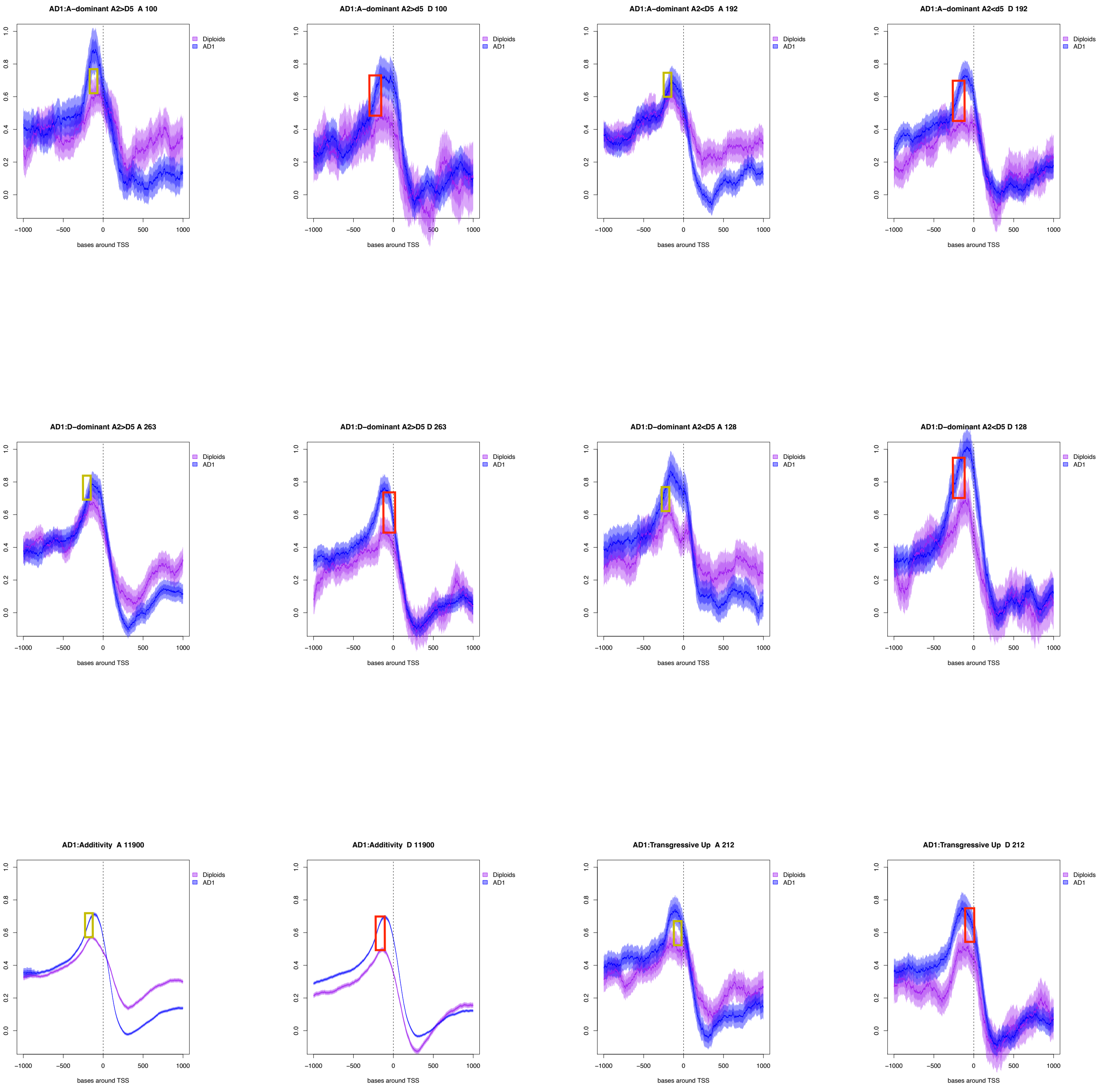

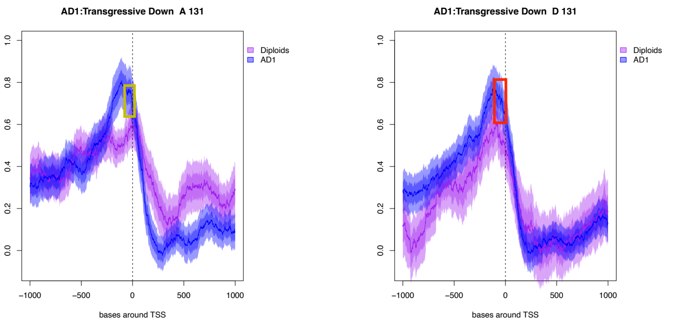

Figure S11. A- and D-genome promoter accessibility based on AD1 references, respectively.
A. HEB and impacts of genome evolution.
B. nonadditive expression in F1.
C. nonadditive expression in AD1.

Figure S12

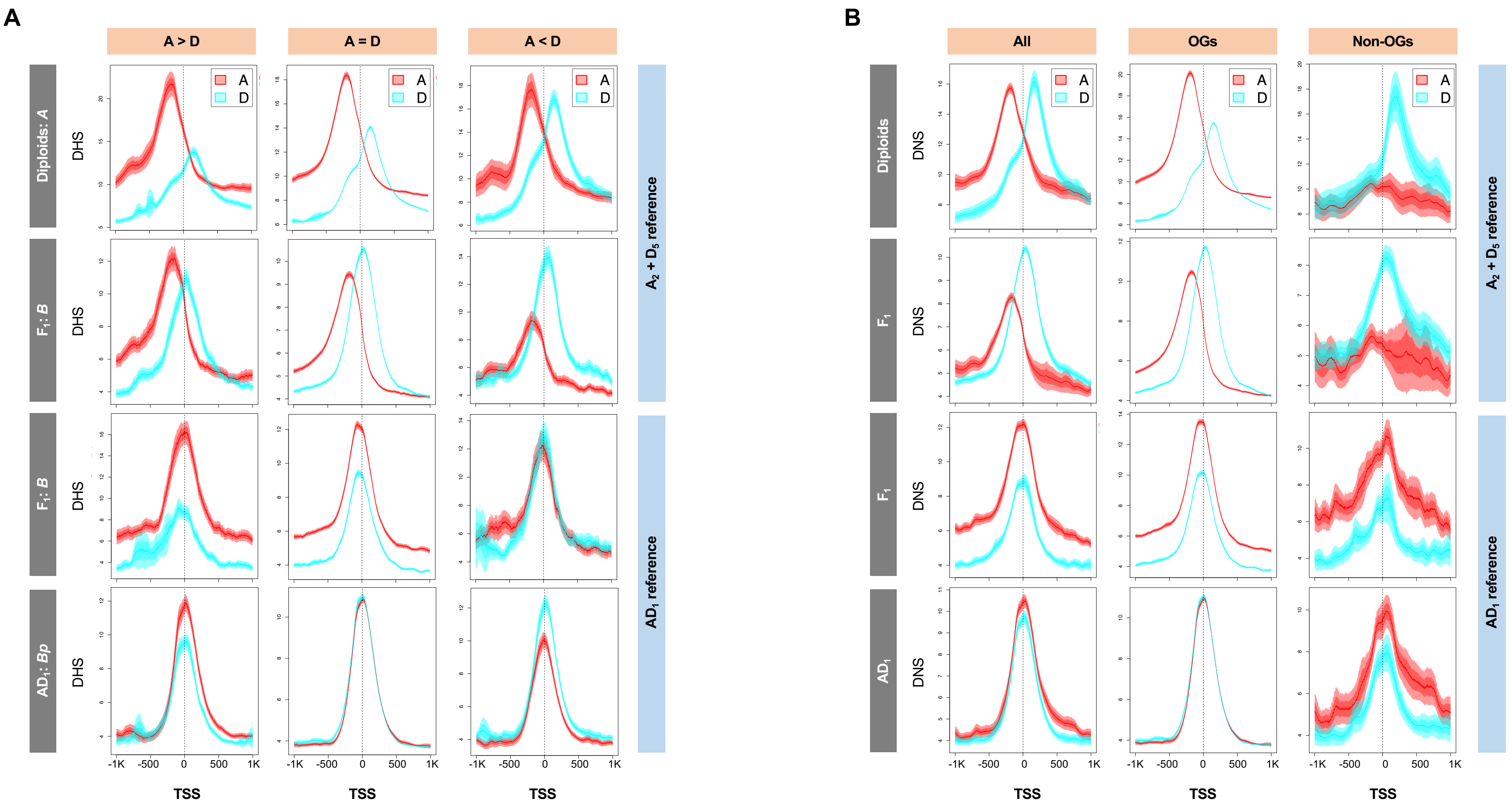

Figure S12. Aggregation plots of chromatin accessibility signals by analyzing a public DNase-seq data (Han et al. 2022) as Figure 7A (A) ad Figure S9 (B).

Figure S13. p1/2

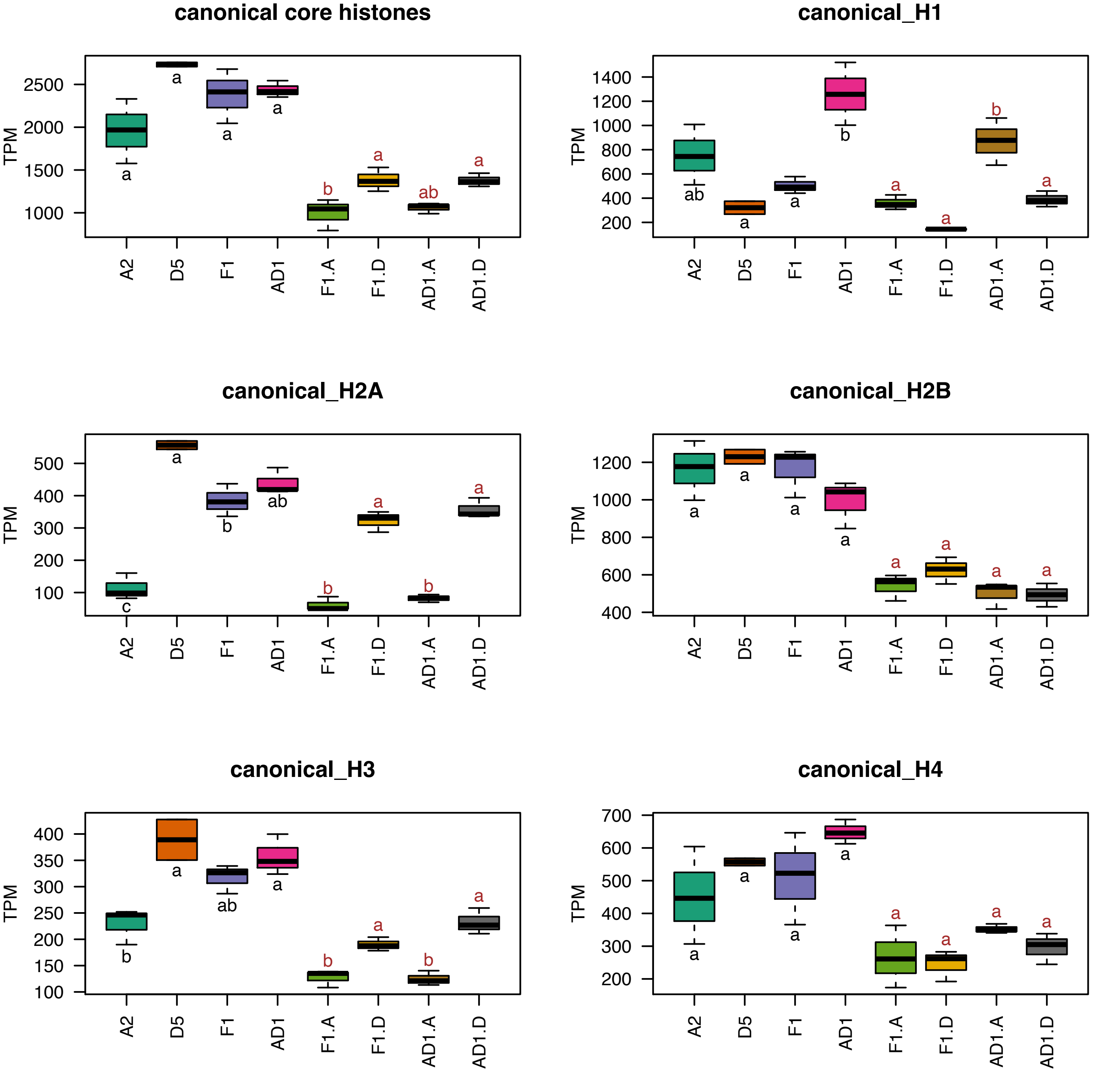

Figure S13. p2/2

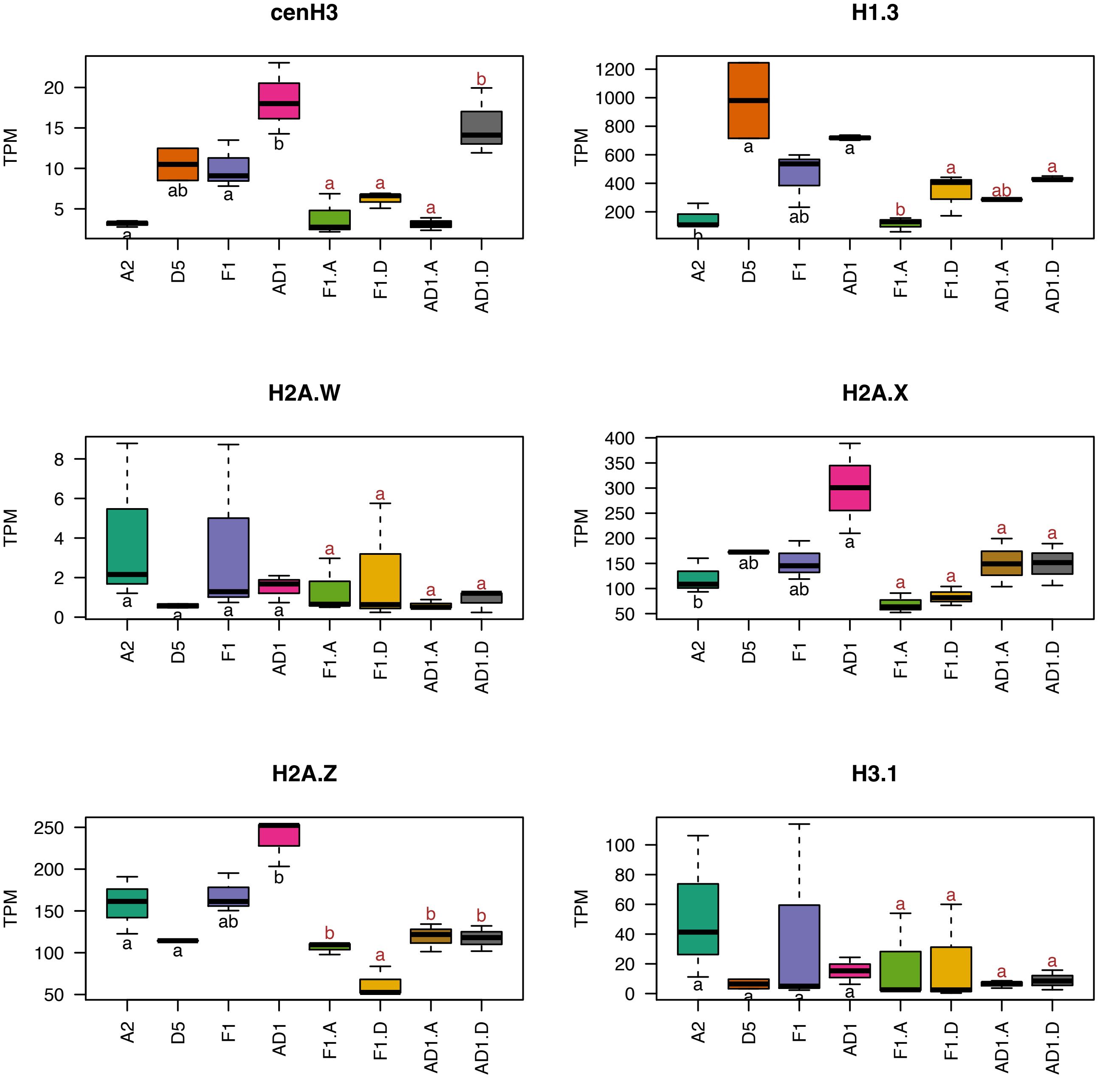

Figure S14

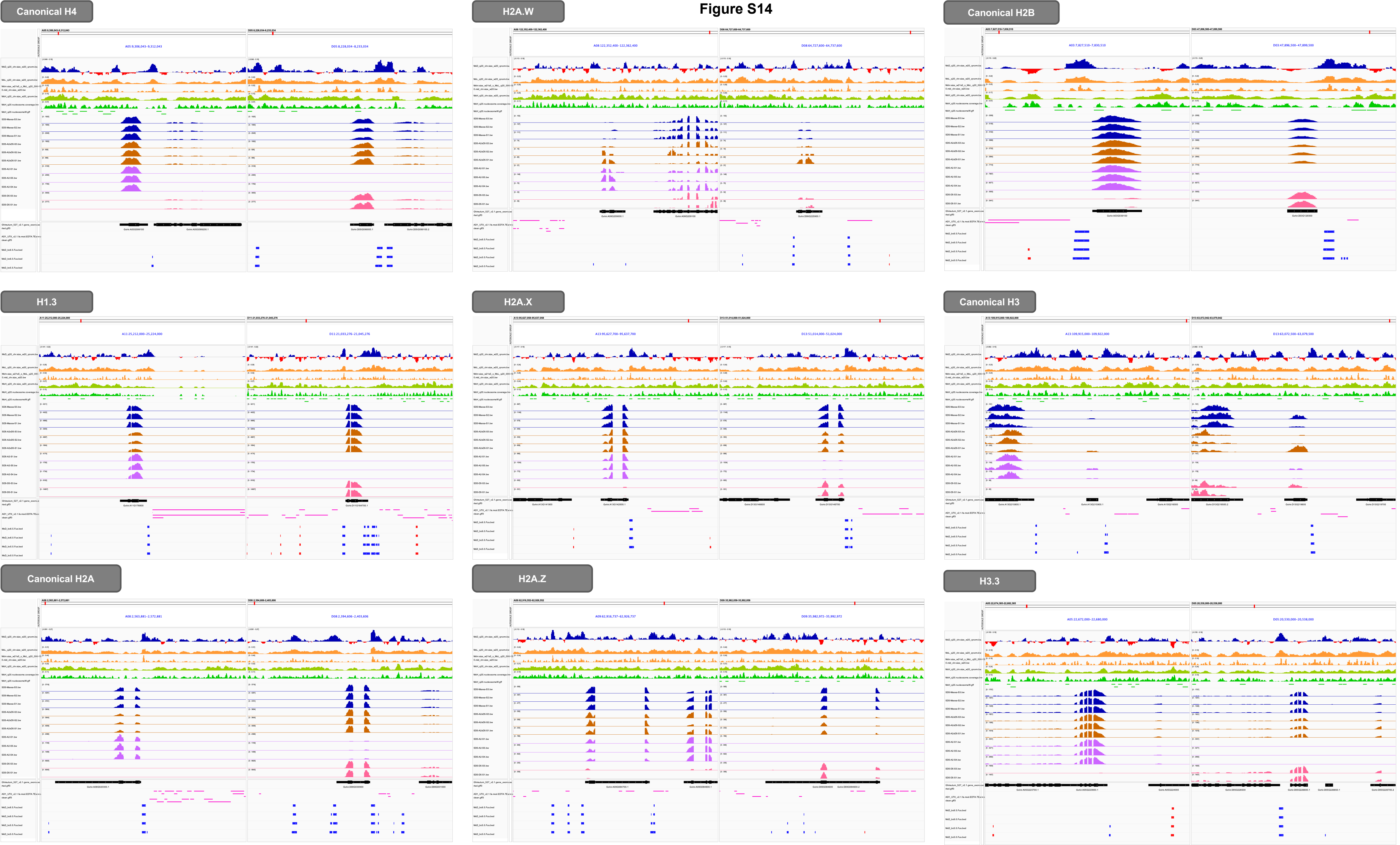

Canonical H4
H2A.W
Canonical H2B
H1.3
H2A.X
Canonical H3
Canonical H2A
H2A.Z
H3.3
